# Comprehensive whole metagenomics analysis uncovers microbial community and resistome variability across anthropogenically contaminated soils in urban and suburban areas of Tamil Nadu, India

**DOI:** 10.3389/fmicb.2025.1649872

**Published:** 2025-10-22

**Authors:** Ramavath Vasanthrao, Illathu Kandy Nidhin, Zarin Taj, Indranil Chattopadhyay

**Affiliations:** Department of Biotechnology, School of Integrative Biology, Central University of Tamil Nadu, Thiruvarur, India

**Keywords:** antibiotic resistance genes, heavy metal resistance genes, virulence genes, microbial diversity, bioremediation, metagenome

## Abstract

**Introduction:**

Environmental contamination by heavy metals and hydrocarbons significantly impacts microbial diversity and soil functionality.

**Methods:**

This study employs whole-genome metagenome sequencing to analyse microbial compositions, antibiotic resistance genes (ARGs), heavy metal resistance genes (HMRs), and virulence genes (VGs) in soil samples from diverse locations, including gardens, poultry farms, cattle sheds, markets, hospitals, thermal power plants, paper industries, and waste disposal sites.

**Results:**

The findings indicate that heavy metal concentrations (Pb, Cr, Cd, and Cu) and hydrocarbons (heptadecane, triacontane, docosane, and heneicosane) were positively correlated with several microbial phyla with relatively high abundances in these contaminated sites, such as Actinobacteria, Proteobacteria, Basidiomycota, Ascomycota, Euryarchaeota, and Apicomplexa. The prevalence of multidrug resistance genes, including *MexD, MexC, MexE, MexF, MexT, CmeB, MdtB, MdtC,* and *OprN,* was significant, facilitating antibiotic resistance primarily via efflux pump mechanisms (42%), followed by antibiotic inactivation (23%) and changes in antibiotic targets (18%). Virulence genes such as *espR, regX3, sigA/rpoV, bap,* and *sugB* were significantly prevalent in contaminated locations, indicating microbial pathogenic potential in polluted ecosystems. The functional gene analysis revealed significant metabolic pathways related to protein metabolism, carbohydrates, amino acids and their derivatives, metabolism, and DNA metabolism, highlighting the microbial adaptation processes engaged in pollution degradation and resource utilisation.

**Discussion:**

This study establishes a clear link between environmental pollution, microbial adaptations, and functional resilience, emphasizing the ecological significance of microbial bio-remediation in shaping targeted remediation strategies and long-term ecological recovery. Understanding these microbial interactions is essential for developing targeted remediation techniques and assessing long-term ecological recovery in contaminated regions.

## Graphical abstract

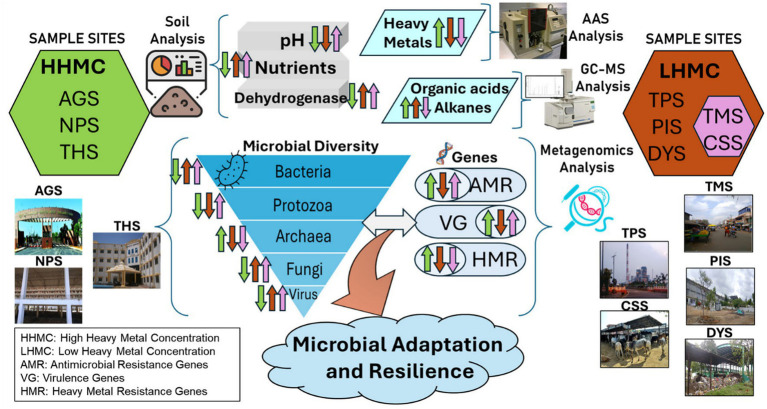

Comparative microbial adaptations in environments with varying levels of environmental niche: The left section presents HHMC sites (AGS, NPS, and THS), whereas the right highlights LHMC sites (TMS, PIS, DYS, TPS, CSS). Key microbial diversity (Bacteria, Protozoa, Archaea, Fungi, Virus) interact with environmental factors (pH, nutrients, heavy metals, organic acids, alkanes), pertaining to AMR, VG, and HMR genes.

## 1 Introduction

Microbes constitute approximately 70% of the planet’s biodiversity and are crucial for the preservation of the environment. The microbial exchange of genes is the fundamental source of diversity among microbes. Antimicrobial-resistant bacteria exist in humans, animals, food, and soil, indicating a transmission dynamic between these entities (Yadav and Kapley, 2021). The soil contains a diverse community of microbes having antibiotic resistance genes. As a result, these alterations to the environment promote horizontal genetic transfer in bacteria and increase tolerance to various stress responses (Cardenas Alegria et al., 2022). Excessive use of antibiotics causes pressure on selective genes, leading to the development, perseverance, and amplification of antibiotic-resistance genes (ARGs) (Lopatkin et al., 2016). Because of the wide range and abundance of microbes, soils serve as a major source of antibiotic-resistant bacteria (ARB) and ARGs (Nesme and Simonet, 2015). The ARGs discovered in the soil are aminoglycosides, erythromycin, fluoroquinolones, sulfonamides, tetracycline, and β-lactams (Cardenas Alegria et al., 2022). Bacteria play a significant role in propagating ARGs in many habitats (Ondon et al., 2021). The significant variability in environmental resistomes could be attributed to several biotic variables, such as secondary metabolites produced by bacteria, and abiotic variables, such as heavy metals, organic contaminants, pesticides, and disinfectants. Environmental variables significantly influence bacterial populations and resistance to environmental stresses. Hydrocarbon pollution has been linked to increased bacterial antibiotic resistance. Research has suggested that polluted places, such as activated sludge and metal-affected environments, can act as hubs for the transmission of resistance genes due to abiotic factors and selective pressure (Das et al., 2021). Several potential co-selective agents for antibiotic resistance include detergents, polyaromatic hydrocarbons, polychlorinated biphenyls, and nanoparticles. Heavy metals are able to reach the environment through natural causes such as geothermal activity, wildfires, corrosion, and erosion, although these are small sources compared with manmade contributors, including industry, agriculture, and hospitals (Gillieatt and Coleman, 2024). Microbes are commonly observed to be reactive and susceptible to heavy metals. When the levels of heavy metals exceed specific limits, these can negatively affect bacterial growth, shape, and important metabolic activities. Continuous contamination with heavy metals causes more pressure on responsive organisms, resulting in significant reductions in abundance and diversity, whereas some organisms can adjust or possibly grow due to the development of protective mechanisms influencing the community of bacteria and their functional characteristics. Soil physicochemical characteristics, for example, can have significant effects on native bacteria, either directly or indirectly, by regulating heavy metal reactivity. Thus, the interaction of soil physicochemical features and heavy metal stress complicates the comprehension of microbial assembly in damaged soils (Zou et al., 2024). Heavy metals cause the simultaneous development of ARGs and metal-resistance genes (MRGs) through co-selection in the environment. Cu contamination has a significant effect on the soil microbial resistome (Liu et al., 2022). Research has shown that ARGs and MRGs can coexist in different contexts. This occurrence can be attributed to two different variables: co-resistance, where ARGs and MRGs are contained inside the identical plasmid, transposon, or integron, and cross-resistance, where ARGs and MRGs have been picked by common pathways. Co-resistance and cross-resistance can result in comparable occurrence trends for ARGs and MRGs (Yan et al., 2024). Tetracyclines and quinolones, ligand-like antibiotics, are capable of producing strong complexes with heavy metal ions such Cu^2+^, Zn^2+^, and Cd^2+^ which may have hazardous effects. Current research suggests that antibiotic-metal compounds can influence ARG recruitment and bacterial community organization, thus impacting the mechanisms of biodegradation (Yan et al., 2024). Cu contamination leads to a significant increase in soil bacterial resistance. Heavy metals cause the simultaneous appearance of ARGs and MRGs through mutual selection (Liu et al., 2022). ARGs were shown to be more prevalent and more abundant in agricultural soils as Ni levels increased (Hu et al., 2017). Heavy metals are regularly added to livestock feed to prevent sickness and promote growth in farm animals (Zhou et al., 2016a). Xu et al. (2017) discovered a favorable link between heavy metal resistance genes such as Cu (pcoA) and Zn (czcD) and specific ARG subtypes in aquatic habitats. Zhou et al. (2016b) reported strong relationships between heavy metals, ARGs, and MRGs in cow dung. ARGs, including multidrug, aminoglycosides, β-lactamases, macrolide-lincosamide-streptogramin, sulphonamides, tetracycline, and vancomycin resistance gene, have also been linked to iron and nickel resistance genes as well (Thomas 4th et al., 2020). Farmhouse soil will absorb manure loaded with various microbiota and quantities of antibiotic-resistant bacteria on the basis of the life phase of the livestock (Liu et al., 2019). The proliferation of ARGs and antibiotic-resistant bacteria (ARB) in humans, animals, and the environment poses significant risks to human well-being and food sustainability (Larsson and Flach, 2022). Antibiotic selection pressure increases the occurrence of AMR in microbes, aggravating ARG variations between habitats. Conversely, habitats with high levels of antibiotics and bacterial burdens promote ARB and ARG release into various ecosystems. Virulence variables play a significant role in increasing pathogenic microbe growth within hosts (Bai et al., 2024). Management implementing a “One Health” approach, spanning the consumption of antibiotics and resistance levels within humans, animals, and the environment in which they interact, is necessary for successfully assessing the development of AMR. Humans may become exposed to AMR infections through the food chain, including the ingestion of infected meat, resulting in food-borne diseases. Furthermore, bacteria that infect both humans and animals can share mobile ARGs at an elevated rate. Additionally, soil bacteria and pathogenic organisms in humans share resistance genes, and it has been proposed that the soil microbiota serves as a repository of resistance gene sequences. Antimicrobial resistance in cattle production influences both the environment and human health (Lawther et al., 2022; Gao et al., 2023). Resistome studies of soil samples offer fresh insights into the environmental resistome, including the discovery of new ARGs (Forsberg et al., 2014). The application of shotgun metagenomics for understanding the structural and functional properties of the environment has grown dramatically in the past few years (Salam, 2024). Metagenomics provides a comprehensive view of the microbial populations and resistome in samples (Noyes et al., 2016). Our hypothesis was that soils with varied heavy metal concentrations and abundances of organic compounds would have distinct resistomes and microbial populations. In the present study, we analyzed the metagenome profile of soil samples from eight different heavy metal and hydrocarbon contaminated sites in Tamil Nadu, India, to investigate alterations in the microbiome and resistome. The primary goals of this research are to determine the total concentrations of heavy metals and the distributions of organic compounds in soils, reveal the microbial and resistome diversity in areas with different types of heavy metal and organic compoundcontamination, and investigate the relationships among the levels of heavy metals, hydrocarbons, and microbial diversity in different soil sites.

## 2 Materials and methods

### 2.1 Site description and soil sample collection

Bulk soil samples were collected from eight different locations with the possibility of becoming polluted from anthropogenic and livestock activities, namely, garden (AGS) (10°48′42.6″N 79°36′55.6″E), poultry farm (NPS) (11°22′51.5″N 78°09′56.7″E), cattle shed (CSS) (10°47′55.4”N 79°35′34.8″E), market (TMS) (10°45′57.0″N 79°38′10.7″E), hospital (THS) (10°46′34.9″N 79°36′11.9″E), dump yard (DYS) (11°30′24.7″N 77°15′27.5″E), paper mill (PIS)(11°29′51.1″N 77°09′26.2″E) and thermal power plant (TPS) (11°35′39.6″N 79°27′25.5″E) (Supplementary Figure 1A). Five replicate samples were collected from each site (at a depth of 10 cm from the topsoil) via alcohol-disinfected trowel, placed into sterile polyethylene bags (Ziploc), combined into a composite sample and subjected to cool conditions in the laboratory within twenty-four hours. During sampling, the climatic conditions were found to be moderate (Lacerda-Júnior et al., 2019). Environmental soil and environmental factors, such as pH and air temperature, can affect microbial community analysis results. To minimize sample bias, we identified locations with identical circumstances and used a five-point stacked sampling method (Zhou et al., 2016b). The soil samples are then air-dried and sieved via a 2 mm sieve. The sieved soil samples were split into two portions and stored at −80 °C and −20 °C for DNA extraction and physiochemical analysis, respectively.

### 2.2 Soil physiochemical and heavy metal analysis

Various techniques were used to examine the following parameters of the soil: pH; nitrogen, phosphorus, total organic carbon (TOC), potassium, magnesium, and calcium contents; and heavy metals such as lead (Pb), nickel (Ni), cadmium (Cd), chromium (Cr), zinc (Zn), copper (Cu), manganese (Mn), iron (Fe), and aluminum (Al). The soil pH was measured following the method of Mclean; soil slurry with 10 g of soil and 20 mL of distilled water was prepared. The slurry was left undisturbed for 30 min. The pH electrode was then inserted into the slurry, and the readings were recorded (Nistala and Kumar, 2023). Five grams of each soil samples was thoroughly mixed with one liter of 1 N NH_4_OAc (pH 7) to determine the contents of potassium, sodium, magnesium, and calcium. The filtrates were then collected using Whatman No. 1 filter paper. The EDTA titration method was utilized to estimate calcium and magnesium, whereas flame photometer was used to assess sodium and potassium (Ukabiala et al., 2021). An atomic absorption spectrophotometer (AAS) was used to analyze Pb, Ni, Cd, Cr, Zn, Cu, Mn, Fe, and Al. To estimate the quantity of heavy metals present, 5 grams of each soil samples were subjected to acid digestion, chilled, and then combined into a 100-milliliter solution. Negative controls or blank samples were also handled comparably. Standard solutions of the corresponding heavy metalswere made at six concentrations. The prepared solution was aspirated into the AAS apparatus in the following order: blank, standard, and sample. The following range of wavelengths, from 200 to 400 nm, was employed to measure the absorbance of various heavy metals: 283.3 nm (Pb), 394.5 nm (Ni), 228.8 nm (Cd), 324.7 nm (Cr), 213.8 nm (Zn), 357.9 nm (Cu), 279.5 nm (Mn), and 248.3 nm (Fe) (Tibebe et al., 2022). The nitrogen, potassium, sodium, and phosphorus contents were measured in kilograms per hectare (kg/ha). Calcium and magnesium were measured in milligrams per kilogram of soil (mg/kg), and total organic carbon was measured as apercentage (%). All heavy metals were measured in the parts per million units (ppm).

### 2.3 GC-mass analysis of the soil samples

The soil samples were analyzed for organic pollutants using GC-mass analysis with slight modifications (Siddiqui et al., 2024). Briefly, 5 g of fresh soil sample was added to a 250 mL Erlenmeyer flask with a 1:1 ratio of methanol: ethyl acetate mixture and shaken vigorously at 250 rpm for 6–8 h. Furthermore, the mixed samples were sonicated for 3 min, 30 s ON, and 30 s OFF mode conditions and centrifuged for 15 min at 10,000 rpm. All the organic pollutants were present in the supernatant; the upper organic phase was collected and dried at 40 °C. The dry residue was dissolved in a methanol: ethyl acetate mixture (1:1, v/v) and filtered through a 0.22 μm syringe filter; samples were analyzed via a GC-mass analyzer (Siddiqui et al., 2024). The organic pollutants were detected and quantified in the soil samples via gas chromatography–mass spectrometry (GC–MS) (Agilent CH-GCMSMS02, 8,890 GC System, 7,000 GC/TQ). Analyte separation was achieved with an HP5MS column (30 m x 250 μm x 0.25 μm) under the following conditions: the injection port temperature was set to 280 °C, and the column temperature was initially held at 50 °C for 1 min before being increased to 280 °C over the course of 38 min. The injector port, interface, and ion source temperatures were set to 120 °C, 210 °C, and 280 °C, respectively. The temperature program for the GC was as follows: start at 50 °C with a hold time of 1 min, then ramp to 120 °C at a rate of 5 °C/min, hold for 1 min, ramp to 210 °C at a rate of 10 °C/min with another 1-min hold, and finally increase to 280 °C at 10 °C/min and hold for 5 min. The total run time was 38 min. A sample injection volume of 2 μL was used with helium as the carrier gas at a 1.0 mL/min flow rate. The transfer line and ion source temperatures were maintained at 210 °C and 280 °C, respectively. The mass spectrometer was operated in full-scan mode, scanned across a mass-to-charge ratio (m/z) range of 30–900, and used the Mass Hunter software for qualitative analysis. (https://www.agilent.com/en/promotions/masshunter-mass-spec). For quantitative analysis, two characteristic ions were detected via single-ion monitoring. Importantly, since this is primarily a qualitative analysis, concentration quantification cannot be performed.

### 2.4 Soil dehydrogenase activity

After being weighed, 0.5 g of the air-dried soil samples were placed into 15 mL centrifuge tubes. Every single tube was meticulously filled with 2 mL of freshly made Tris–HCl buffer (pH 7.6) and 1 mL of 1% (w/v) triphenyl tetrazolium chloride (Sisco Research Laboratories Pvt. Ltd., India). After thoroughly vortexing the tubes to completely combine the reaction mixture, the tubes were incubated for 24 h at 37 °C in the dark. Ten milliliters of 96% ethanol were used to obtain the triphenyl tetrazolium formazan (TPF) that had developed after 24 h. The tubes were vortexed and centrifuged at 4,000 x g for 5 min. The red color intensity at 485 nm was used to measure the quantity of TPF in the supernatant via a UV/Vis spectrophotometer (Eppendorf AG 22331 Hamburg) (Seo and Cho, 2021).

### 2.5 DNA extraction from the soil samples

Air-dried and sieved soil samples were used for the DNA extraction via the DNeasy Power Soil Pro Kit (QIAZEN, Hilden, Germany), according to the manufacturer’s instructions. The quality and quantity of the isolated DNA were determined via a Nanodrop spectrophotometer (Genova Nano, Jenway, UK) with a 260/280 nm ratio. DNA integrity was confirmed via 1% agarose gel electrophoresis (Lacerda-Júnior et al., 2019).

### 2.6 Whole metagenome sequencing using the Illumina Hiseq platform

The extracted DNA samples were fragmented via the KAPPA fragmentation method (KAPA HyperPlus Kit) to fragment the DNA into 600 bp length. The fragmented samples were processed for end repair and A-tailing with Hypapeep plus ERAT enzyme mixture. Immediately after end repair and A-tailing the adapter was added and ligated to the end repaired DNA fragments via DNA ligase. Library amplification was performed on the adapter ligated samples with Illumina primers (Illumina, San Diego, USA). A total of 40 ng of extracted DNA was used for amplification, along with 10pM of each primer with initial denaturation at 98 °C, 4 Cycles of denaturation at 98 °C for 15 s, annealing at 60 °C for 30 s, extension at 72 °C for 30 s, and a final extension at 72 °C for 1 min and followed by holding at 4 °C. Libraries were purified via Ampure beads and quantitated using a Qubit dsDNA high sensitivity assay kit. Sequencing was performed via Illumina Hiseq 4,000 (Illumina, San Diego, USA) at Biokart India Pvt. Ltd. (Banglaore, India). The size of the product used for sequencing was >450 bp.

### 2.7 Bioinformatics analysis

Quality control (QC) was performed on the raw sequenced data via fastQC (https://www.bioinformatics.babraham.ac.uk/projects/fastqc/). The raw data were trimmed to remove the adapter sequences via the tool Trimgalore version 0.4.5 (https://www.bioinformatics.babraham.ac.uk/projects/trim_galore/). The trimmed raw data were used as the input for Kraken2 analysis. Kraken2 version 2.1.2 software was used for further analysis (Lu et al., 2022). The Kraken-build parameter was used to build databases for the analysis. The source used to construct the database was downloaded from NCBI (National Center for Biotechnology Information). The cluster of Orthologous Genes (COG) classifier version 1.0.5 (https://github.com/moshi4/COGclassifier/) was used to perform the COG functional annotation. The COG database is used for the functional annotation of genomes by providing a set of clusters of orthologous genes that can be used to predict the function of uncharacterized genes on the basis their similarity to known genes. COGs are defined as groups of proteins that are likely to be orthologs, which are genes in different organisms that evolved from a common ancestor by speciation, and thus retain the same function. COGs are identified by comparing the complete proteomes of multiple organisms and clustering the proteins into groups on the basis of sequence similarity and phylogenetic analysis. The Kyoto Encyclopedia of Genes and Genomes (KEGG) database annotation refers to the process of assigning functional information to genes or proteins via the KEGG database (https://www.genome.jp/kegg/pathway.html). A gene or protein sequence is compared to the sequences in the KEGG database using sequence alignment tool BLAST. The gene or protein is assigned to a KEGG pathway or function on the basis of the best match. The graphs have been made with the top 10 pathways and pathway functions. MEGAN6 software was used to construct the phylogenetic tree at the taxon level (Gautam et al., 2023). Virulence factors and antimicrobial resistance (AMR) genes were predicted by performing a BLAST search of protein files against the Virulence factor and CARD database (https://card.mcmaster.ca/) of the respective samples, with a gene identity of 95%. The Ghostkoala tool (https://www.kegg.jp/ghostkoala/) was used for pathway annotations. Metageneassist (http://www.metagenassist.ca/METAGENassist/) was used to predict metabolic genes. Microbiome analyst (https://www.microbiomeanalyst.ca/)was used to predict alpha diversity, beta diversity, rarefaction curve, dendogram, correlation plot, and core microbiome. Sankey plots are visualized with the Pavian R based tool (https://ccb.jhu.edu/software/pavian/). The SEED subsystems database has been used to predict the subsystem. STAMP 2.1.3 (https://beikolab.cs.dal.ca/software/STAMP) was used to generate extended error bar charts. The comparative analysis of metabolic pathways within SEED subsystems was conducted using Fisher’s exact test by STAMP software. A *p* ≤ 0.05 filter was used. Protein sequences are compared to sequences in the BacMetdatabase (http://bacmet.biomedicine.gu.se/) using sequence alignment tools such as BLAST. The graphs have been made with the top 20 heavy metals with at least 60% identity. Unit variance scaling is applied to sequence abundance. Both taxon rows and sample columns were clustered using correlation distance and average linkage. The top 50 organisms were used for heatmap construction by clustiviz web tool (https://biit.cs.ut.ee/clustvis/). Unit variance scaling is applied to rows; SVD with imputation is used to calculate principal components. The X and Y axes show principal component 1 and principal component 2 which explain the total variance by clustviz web tool.

## 3 Results and discussion

### 3.1 Soil physiochemical and heavy metal analysis

**Figure 1:**
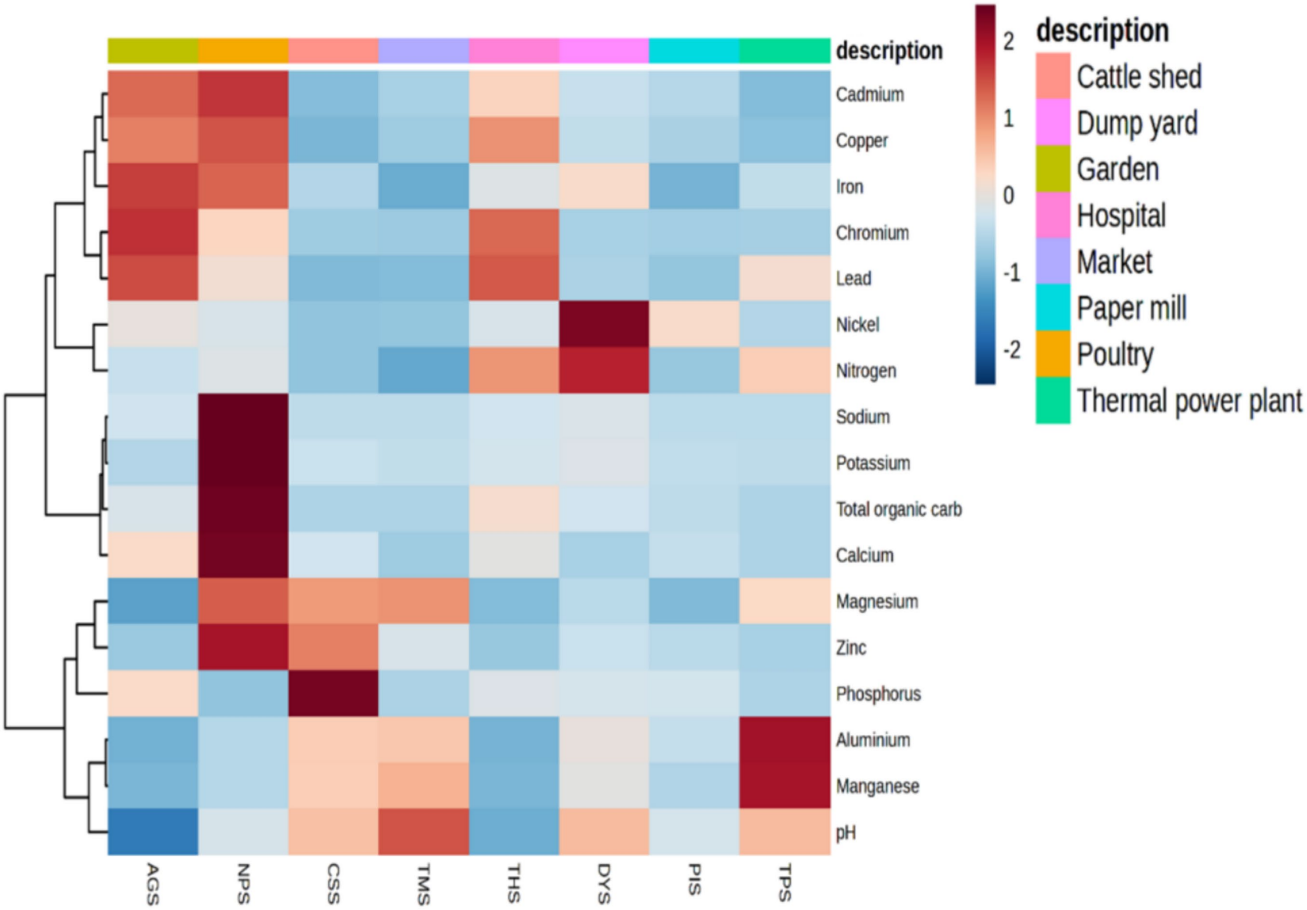
Heat map illustrating the areas where heavy metals and soil physiochemical properties cluster by using wards clustering methods and the Euclidean distance matrix. The scale displays the Z score measures, with blue signifying low concentration and dark red representing high concentration. A heatmap is a two-dimensional illustration of data that uses colors to represent values and provide instant visual information.

All the samples had pH values ranging from neutral to alkaline. Our results revealed that poultry farm soil (NPS) had markedly higher concentrations of K (1,299 kg/ha), Na (3,963 kg/ha), Ca (mg/kg), Mg (833 mg/kg), and total organic carbon (TOC) (6.71%) than the other samples. The highest concentrations of metals such as Cu (4.872 ppm), and Cd (0.316 ppm) were reported in the NPS sample. N (478.93 kg/ha) and Ni (0.79 ppm) were the highest in the DYS sample, and Pb (4.08 ppm), Fe (169 ppm), and Cr (2.41 ppm) were found to have the highest concentration in the AGS sample. The highest concentrations of Al (628.15 mg/kg) and Mn (1889.32 mg/kg) were reported in the TPS sample. The CSS sample had the highest concentration of P (219.4 mg/kg) and the lowest concentrations of Pb (0.007 ppm), Ni (0.01 ppm), Cr (0.042 ppm), Cu (0.347 ppm), and TOC (0.114%). The lowest concentrations of calcium (111.6 mg/kg) and iron (60.3 ppm) were reported in the TMS sample. Moreover, the lowest quantities of Na (71.6 kg/ha) and Cd (0.061 ppm) were detected in the PIS and TPS samples, respectively, and Na (85.86 kg/ha) and Cd (0.014 ppm) were detected in the TPS samples (Supplementary Table 1). Principal components analysis (PCA) revealed that the NPS sample was clearly distinct from the remaining samples. The THS sample showed close similarity to the AGS sample. TPS, TMS and CSS were grouped together, at the same time; AGS, THS, PIS and DYS were grouped together (Supplementary Figure 1B). The heatmap generated concerning the Figure 1 displayed the soil properties vertically and the location of the study site horizontally. A heatmap is a two-dimensional illustration of data that uses colors to represent values and provide instant visual information. The scale displays the Z score measures, with blue signifying low concentration and dark red representing high concentration. By using cluster analysis, objects (cases) can be grouped into classes (clusters) based on similarities within each class and dissimilarities between different classes. The results of cluster analysis help in interpreting the data and indicating patterns. Lead, cadmium, chromium and copper were higher in three samples, namely, AGS, THS and NPS; these samples were grouped together as high heavy metal concentration (HHMC) samples and the other five samples with lower concentrations of these heavy metals, namely, CSS, TMS, TPS, DYS and PIS were grouped as low heavy metal concentration (LHMC) samples. Cadmium, lead, and chromium are the most hazardous compounds that can harm the environment (Chen et al., 2018). The soil pH was positively correlated with Al, Mn, and Mg. Moreover, soil pH had significant negative correlations with Ca, Cr, Fe, Cu, and Pb. Cu was negatively correlated with soil pH, Al, and Mn. Moreover, Cu was positively correlated with Pb, Fe, Cd, Cr, Ca, and TOC. Cd had significant positive correlations with Cr, Fe, TOC, Ca, Cu, and Na. Na and K were positively correlated with Zn, Ca, and TOC. Ca and TOC were positively correlated with potassium, sodium, copper, and zinc (Supplementary Figure 1C). Although zinc, copper, iron, and chromium are critical minerals for soil microorganisms, excessive deposits can cause oxidative damage and disrupt protein assembly and activity (Salam, 2022). Copper is commonly used in pesticide and antibacterial manufacturing, and is integrated into food for livestock to increase development by affecting microbes in the gut (Pal et al., 2017).

### 3.2 Distribution of various hydrocarbons and other organic compounds across the different soil sites

**Figure 2:**
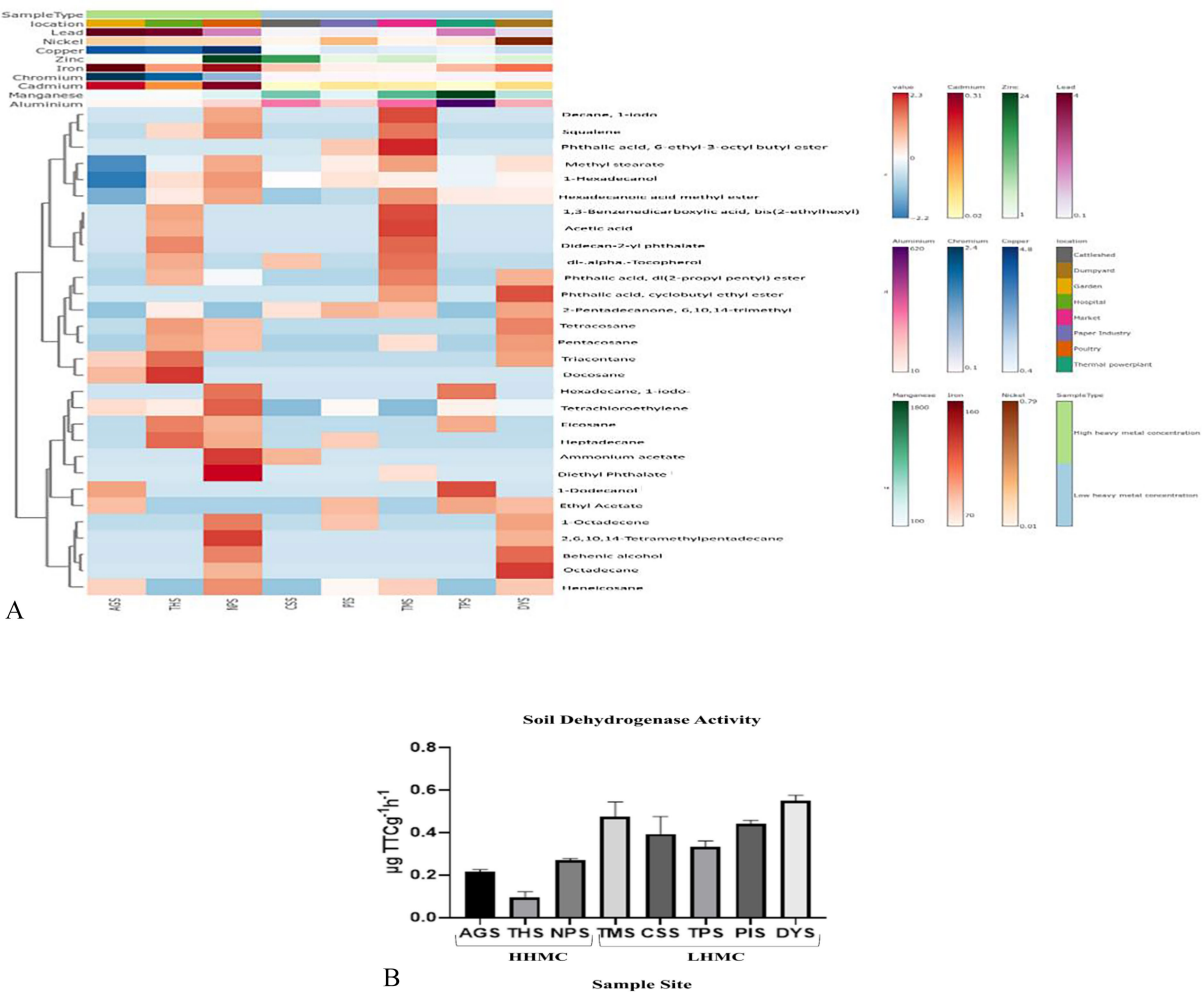
**(A)** A heat map depicting the distribution of top thirty metabolites having highest peak areas across all samples. **(B)** Soil dehydrogenase activity of eight samples, expressed in μg TTC g^−1^ h^−1^.

The heatmap (Figure 2A and Supplementary Table 2) revealed that among the HHMC samples, AGS samples presented a greater peak area of 1-dodecanol and docosane followed by the THS sample had a higher peak area of docosane, triacontane, heptadecane, eicosane, and didecan-2-yl phthalate. NPS had a greater peak area fordiethyl phthalate, ammonium acetate, squalene, 2, 6, 10, 14-tetramethylpentadecane, tetrachloroethylene, hexadecane, decane, 1-iodo. Moreover, among the LHMC samples, the peak area of ammonium acetate was greater in the CSS samples. PIS had a greater peak area for 2-pentadecanone, 6, 10, 14-trimethyl. TMS had a greater peak area of decane, 1-iodo, phthalic acid, 6-ethyl-3-octyl butyl ester, 1, 3-benzenedicarboxylic acid, bis (2-ethylhexyl), and dl-alpha–tocopherol. The TPS had a greater peak area of 1-dodecanol, hexadecane, 1-iodo. DYS had a greater peak area of octadecane, phthalic acid, cyclobutyl ethyl ester, and behenic alcohol. Using the ProTox-II web server, we analyzed the toxicity and adverse effects of the metabolites found in the soil samples, including their LD50 values and severe hepatotoxicity, cytotoxicity, carcinogenicity, immunotoxicity, and mutagenicity characteristics. Carcinogenicity was exhibited by the compounds such as, tetrachloroethylene, hexadecane, 1-iodo-, hexadecanoic acid, ethyl ester, phthalic acid, di (2-propylpentyl) ester, ethyl acetate, phthalic acid, di (2-propyl pentyl) ester, (E)-9-octadecenoic acid ethyl ester, 1,3-benzenedicarboxylic acid, bis (2-ethylhexyl), pentadecanoic acid, ethyl ester, phthalic acid, and 6-ethyl-3-octyl butyl ester (Supplementary Table 3). Most of the compounds represent the predominant classes of hydrocarbons, alkanes, acyclic alkanes, and fatty acids (Supplementary Figure 2).

N-docosane is usually present in petroleum pollution. *P. aeruginosa* DQ8 mostly degrades n-docosane by terminal oxidation (Sui et al., 2023). Diethyl phthalate (DEP), a frequently employed plasticizer, has sparked widespread public outrage because of its ease of identification, ecological stability, and possible adverse health effects (Yu et al., 2021). Squalene is frequently employed in the culinary, cosmetic, and pharmaceutical sectors for several purposes (Lu et al., 2018). Petroleum hydrocarbons are eventually discharged into soils as a result of container spills and leaks at gas pumps and facilities for industry. The extensive and permanent existence of pollutants derived from petroleum hydrocarbons represents major dangers to human well-being, including carcinogenesis, toxicity, and gene alterations that can be transferred through the food chain. Because of the enhanced electrostatic repulsion between n-hexadecane and soil particles, the ability of the compound to penetrate cadmium-contaminated soil was far greater (Huang et al., 2023).

### 3.3 Soil dehydrogenase activity

Among various soil enzymes, dehydrogenase activity (DHA) has been recognized as an important biochemical indicator in soil introducing the concept of determining the metabolic activity of microorganisms in soil and other habitats by measuring DHA. DHA activity is a sign of oxidative metabolism and microbiological activity in soils because it is related to viable cells (Velmourougane et al., 2013). The AGS sample was reported to have a soil dehydrogenase activity value of 0.217 ± 0.01 μg TTC g^−1^ h^−1^. The soil dehydrogenase activity of the CSS, TPS, TMS, and PIS samples rangedfrom 0.32 to 0.48 μg TTC g^−1^ h^−1^. A markedly higher dehydrogenase activity was observed in the DYS sample (0.549 ± 0.03 μg TTC g^−1^ h^−1^), whereas, a much lower soil dehydrogenase activity was reported in the THS sample (0.094 ± 0.03 μg TTC g^−1^ h^−1^). The ANOVA-test revealed that the soil dehydrogenase activity was significantly differed across all the samples (*p* value 0.0037) (Figure 2B). Low organic content in hospital soil can reduce microbial diversity and dehydrogenase activity (Basiry et al., 2022). Poultry litter can contain high levels of ammonia that can inhibit microbial activity (Crippen et al., 2019). Cattle manure with decreased ammonia content might hinder soil microbes (Black et al., 2021). The waste from livestock nourishes soil bacteria with organic matter, facilitating the production of dehydrogenase via metabolism (Zhu et al., 2023). Composted food remnants in market soils serve as a substantial nutrient reservoir for microbes, leading to an increased and dynamic microbial community. Fertilizers or composts enrich soil with essential nutrients, enhancing dehydrogenase activity (Medo et al., 2021; Carpio et al., 2020). Gardens with increased organic matter content, such as compost and leaves, provide a food source for microbes, resulting in an active microbial population and higher DHA (Woźniak et al., 2024). Organic substances found in dump yards, such as food leftovers, yard trash, and paper products, might attract soil microbes, leading to enhanced microbial and dehydrogenase activity (Rastogi et al., 2020). Paper mills emit harmful substances such as chlorines, dyes, and heavy metals into the surroundings, resulting in contamination of soil and alterations of microbial communities (Mohan and Shukla, 2022; Turner et al., 2022).

### 3.4 Overview of the metagenomic sequencing results

Metagenomic sequencing yielded 23.78 ± 18.62 million reads per soil sample, with a mean GC content of 62% ± 0.02, and a mean quality Phred score of 35.69. Overall, the average percentage of each Kingdom was3.11% Archaea, 95.47% Bacteria, 0.56% Eukaryota fungi, 0.40% Eukaryota protozoa, and 0.30% Viruses.

### 3.5 Bacterial diversity across the soil samples

**Figure 3:**
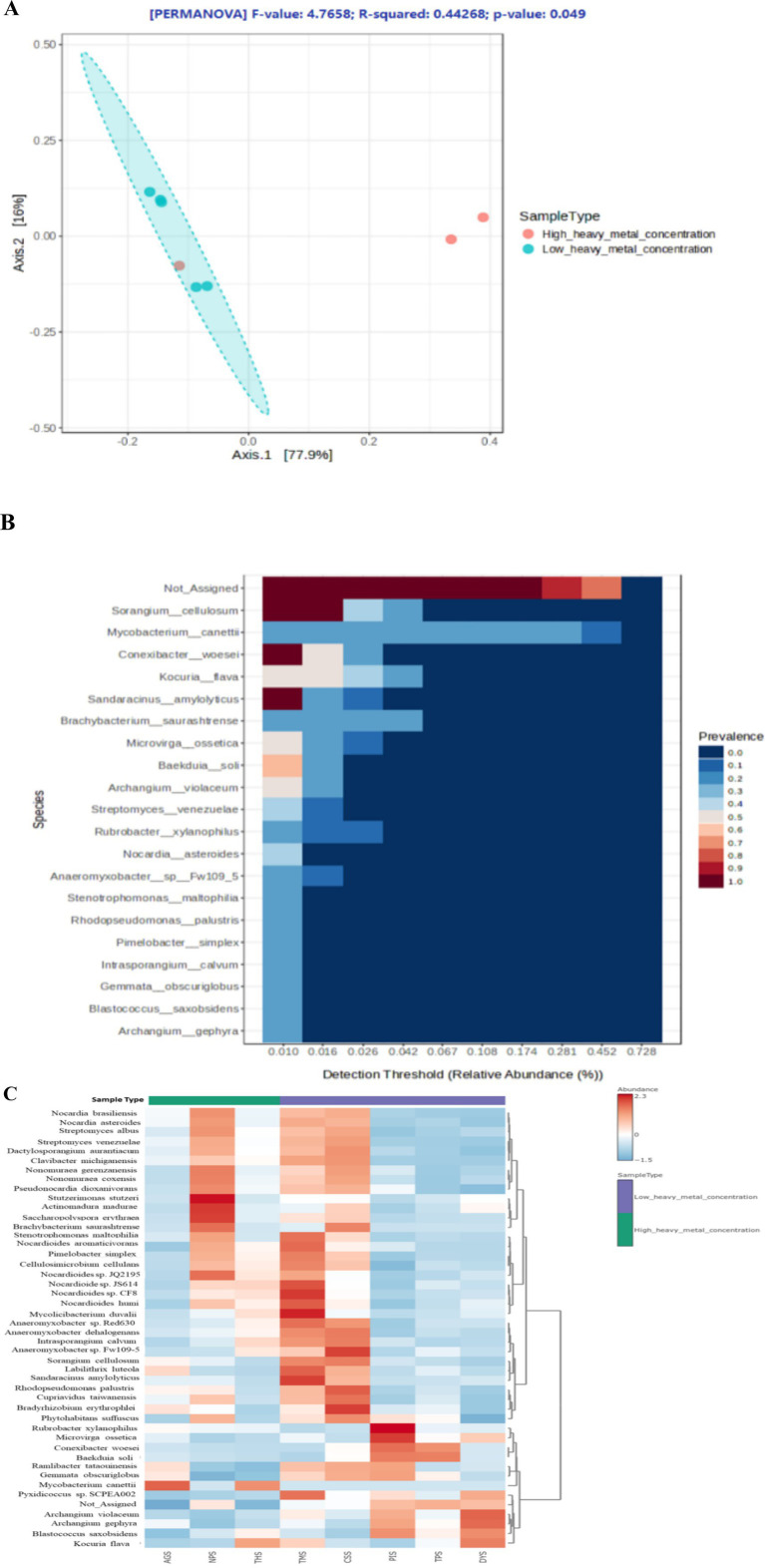
**(A)** PCoA plot showing Beta diversity of bacteria across the samples by using ordination, Bray-Curtis index distance, and PERMANOVA statistical method; **(B)** Heat map illustrating the core microbiome abundance across all the samples. The core microbiome refers to the set of taxa that are detected in a high fraction of the population above a given abundance threshold. The count data is transformed to compositional (relative) abundance in order to perform such analysis. **(C)** Heat map illustrating the bacterial species level abundance across all the samples.

The rarefaction curves approached saturation, suggesting full bacterial abundance (Supplementary Figure 3). Supplementary Figure 4 depicts the representation of alpha diversity indices across the samples. The two HHMC samples, AGS and THS, had lower Shannon and Simpson indices, indicating reduced bacterial diversity. The LHMC samples CSS and TMS presented the highest Shannon and Simpson indices, indicating a diverse bacteriome. Interestingly, the NPS samples presented greater diversity, unlike the other HHMC samples. The non-significant *p*-values (≥ 0.05) of Simpson (0.29) and Shannon (0.40) alpha diversity indices indicate that the bacterial ecological niche is the same among the ‘high heavy metal concentration’ (HHMC) and ‘low heavy metal concentration’ (LHMC) samples. The beta diversity plot depicts statistically significant variations (*p*-value = 0.049) between the two groups of samples, which had an *F*-value of 4.77 and an R-squared value of 0.44. Significant inter-group variations in the bacteriome existed between the HHMC and LHMC sample groups. Among the HHMC samples, the AGS and THS samples were separated from the LHMC samples, whereas the NPS samples were closely associated with the LHMC samples (Figure 3A). The PCA score plot (Supplementary Figure 5A) of the first two main components clearly demonstrates that high and low heavy metal concentrations constitute the fundamental foundation for separation, with PC1 and PC2 accounting for 97.1 and 2.2% of the variation, respectively. High-heavy metal concentration samples, such as AGS and THS, were well separated from low-heavy metal concentration samples, except for NPS, where the NPS samples overlapped with low-heavy metal concentration samples (CSS, DYS, TMS, TPS, and PIS). The 3D plot with three principal components (Supplementary Figure 5B) revealed that the samples from the cattle shed (CSS), dump yard (DYS), market (TMS), poultry (NPS), and thermal power plant (TPS) were grouped together. In contrast, hospital (THS), paper Industry (PIS), and garden (AGS) samples were separated from all the other samples. The 3D PCA plot showing three principal components (Supplementary Figure 5B) reveals that the first principal component (PC1) explains 77.9% of the total variance, whereas PC2 and PC3 account for 16 and 5.4%, respectively. These results indicate a clear separation between the HHMC and LHMC groups (Supplementary Figure 5B). Atthe phylum level of overall taxonomic profiling, Actinobacteria was found to be predominant with highest diversity followed by Proteobacteria, and Planctomycetota (Supplementary Figure 5C). The stacked bar plot (Supplementary Figure 5C) shows that the HHMC samples (AGS, THS, and NPS) have a higher abundance of bacteria than the LHMC samples. Actinobacteria was the most abundant phylumin all the samples, accounting for 75.65, 53.96, 70.91, 65.19, 66.67, 81.06, 58.39, and 66.79% of the effective bacterial sequences from AGS, CSS, DYS, NPS, PIS, THS, TMS, and TPS, respectively. The nextmost dominant phylum in the AGS, CSS, DYS, NPS, PIS, THS, TMS, and TPS samples was Proteobacteria, with a prevalence of 23.52, 44.96, 28.58, 34.51, 32.20, 18.58, 40.70, and 32.41%, respectively. Actinobacteria was the most abundant phylum, followed by Proteobacteria and Planctomycetota between the two groups. *Actinomycetia* was the most abundant class, followed by *Deltaproteobacteria* and *Alphaproteobacteria*. *Corynebacteriales* was the most abundant order, followed by *Streptomycetales* and *Myxococcales*. *Mycobacteriaceae* was the most abundant family, followed by *Streptomycetaceae*, *Nocardioidaceae*, and *Nitrobacteriaceae*. At the species level, *Mycobacterium canettii* was the most abundant, followed by *Sorangium cellulosum,* and *Conexibacter woesei*. *Mycobacterium canettii* was predominant in the AGS (49.76%) and THS (40.54%) samples. *Sorangium cellulosum* was predominant in the AGS, CSS, DYS, NPS, PIS, THS, TMS, and TPS samples, with prevalence rates of 2.91, 4.50, 1.68, 2.42, 2.18, 1.92, 4.38, and 1.97%, respectively. *Kocuria flava* was predominant in DYS (5.43%), THS (4.66%), and TMS (3.37%) samples (Supplementary Figure 5D). The core microbiome refers to the set of taxa that are detected in a high fraction of the population above a given abundance threshold. *Sorangium cellulosum* was the most dominant species in the core microbiome, followed by *Mycobacterium canettii, Conexibacter woesei, Kocuria flava, Sandracinus amylolyticus, Brachybacterium saurashtrense, Microvirga ossetica, Baekduia soli, Archangium violaceum, Streptomyces venezuelae, Rubrobacter xylanophilus*, and *Nocardia asteroids* (Figure 3B). The heat map (Figure 3C) depicts the distribution of the selected bacterial species across the samples. Among the HHMC samples, *Mycobacterium canettii* was abundant in the AGS samples, whereas *Stutzerimonas stutzeri*, *Actinomadura madurae*, and *Saccharopolyspora erythraea* were abundant in the NPS samples. *Kocuria flava* and *Mycobacterium canettii* were predominant in the THS. Among the LHMC samples, the CSS samplespresented high abundances of *Brachybacterium saurashtrense* and *Bradyrhizobium erythrophlei* species, the PIS samplespresented high levels of *Rubrobacterxylanophilus* and *Microvirgaossetica* species; and the TMS samplespresented high levels of *Mycolicibacterium duvalii*, *Nocardioides* sp. CF8, and *Sandaracinus amylolyticus* species. *Mycobacterium cannettii* was abundant in the HHMC samples. S*treptomyces* sp., *Bradyrhizobium and Nocardioides* were comparatively less abundant in HHMC than in LHMC. The correlation plot (Supplementary Figure 6) shows that most of these bacterial genera were positively correlated with one another, with only a few negative correlations. *Azospirillum, Dactylosporangium, Rhizobium, Burkholderia, Gordonia, Pseudomonas, Xanthomonas,* and *Achromobacter* were highly positively correlated with each other. *Nocardioides* was negatively correlated with many bacteria, mainly *Azospirillum, Dactylosporangium, Rhizobium, Burkholderia, Gordonia, Pseudomonas, Xanthomonas,* and *Achromobacter*.

Actinobacteria, including *Streptomyces*, *Nocardia*, and *Kocuria* can aerobically breakdown organic molecules (Behera and Das, 2023). Guo et al. (2017) reported that in heavy metal-contaminated locations, Proteobacteria and Actinobacteria dominated the microbial population (Guo et al., 2017). Actinobacteria are known for producing spores that are resistant to external stressful circumstances, secreting proteins that breakdown soil macromolecules. These resistance mechanisms include resistance to heavy metals and antibiotics, and the biosynthesis secondary compounds and antibiotics, providing unique benefitsunder soil conditions. Proteobacteria have unique structural, biological, and metabolic characteristics that enable them to thrive in oligotrophic settings and handle different kinds of stress (Salam, 2022). Proteobacteria are copiotrophs that are capable of utilizing carbon obtained from trees. The prevalence of Proteobacteria tends to increase when organic matter levels in the soil increase. An increase in Proteobacteria richness is usually associated with improved microbial tolerance to harmful chemicals and the condition of the soil (Zou et al., 2024). Proteobacteria are the main active phyla in sediments polluted with high quantities of heavy metals (e.g., As, Cu, Cd, Hg, Pb, and Zn) (Song et al., 2021). Proteobacteria are the predominant bacteria in polluted environments because of their high tolerance to heavy metals (Qiao et al., 2021). Alphaproteobacteria include primary producers, plant mutualists, and pathogenic microorganisms (Williams et al., 2007). *Nocardioides* are involved in the degradation of hydrocarbon and haloalkane pollutants through the activation of genes such as *phdA, phdB, phdC,* and *phdD* (Ma et al., 2023). The abundance of heavy metals in the soil could cause competition for resources among soil microorganisms, altering the variety of microbial life in the soil. The presence of heavy metals affects microbial diversity and growth because bacteria are susceptible to heavy metals. Most investigations have shown that heavy metal exposure can drastically reduce the soil bacterial population. *A*reas with intermediate heavy metal contamination have greater bacterial diversity than sites with low or high levels of heavy metal contamination (Ma et al., 2022). Other studies have shown that heavy metal contamination has no substantial effect on the diversity of bacteria (Dell’Anno et al., 2021). *S. cellulosum* strains are an invaluable resource for discovering new and potential commercial lipolytic enzymes. The cellulolytic myxobacterium *S. cellulosum* not only is very appealing in drug discovery, but also has significant breakdown capabilities for a wide spectrum of macromolecules, including lipids and polysaccharides (Yuan et al., 2023). *B. saurashtrense* JG06, a new diazotrophic plant growth boosting bacterium, increases peanut plant tolerance by modifying physio-biochemical activity and host-gene transcription under nitrogen deprivation circumstances (Alexander et al., 2021). The wild-type, soil-dwelling bacterium *S. venezuelae*, which creates aerial hyphae and spores as a component of its regular growth process, also produces the alkaline volatile chemical trimethylamine (TMA) under various circumstances for growth (Zhu et al., 2024). *Stutzerimonas stutzeri* strains reduce N₂O release in soils with different appearances, likely due to changes in the makeup of soil microbial communities and gene expression associated with nitrification and denitrification (Gao et al., 2024). Saccharopolyspora bacteria such as *Sac. erythraea* generate essential polyketide antibiotics, such as erythromycin A (Lü et al., 2020). *S. amylolyticus* typically exhibits myxobacterial properties such as gliding motility, secondary metabolite synthesis, and spore development under adverse environmental circumstances. *S. amylolyticus* degrades agar, chitin, cellulose, and starch (Sharma et al., 2016).

*Mycobacterium canettii* is a smooth bacillus that belongs to the *Mycobacterium tuberculosis* complex. *M. canettii* exposure primarily manifests as lymph node and pulmonary tuberculosis. It also has the ability for intra-species transfer of genes horizontally (Bouzid et al., 2017). *Kocuria* spp. constitutes part of the typical human flora and can be isolated from a variety of ecological habitats. In particular, these bacteria live on human skin and mucus membranes, such as the mouth cavity, and are generally regarded as non-pathogenic. Infections are most typically observed in patients with immunodeficiency, deformities, intensive care individuals, and even newborns; nevertheless, an increase in *Kocuria* infections has recently been reported in individuals who are immunocompetent (Ziogou et al., 2023). Relative abundance analysis showed that several *Streptomyces* sp. were detected in higher abundance in sediment samples from the Ganga than the Yamuna of India (Behera et al., 2020). Pseudomonadota and Actinomycetota were found in the polluted sediment samples from the river Ganga, contaminated with high concentrations of heavy metals such as Mn, Cr, and Fe (Rout et al., 2024a).

### 3.6 Archaeal diversity across the soil samples

**Figure 4:**
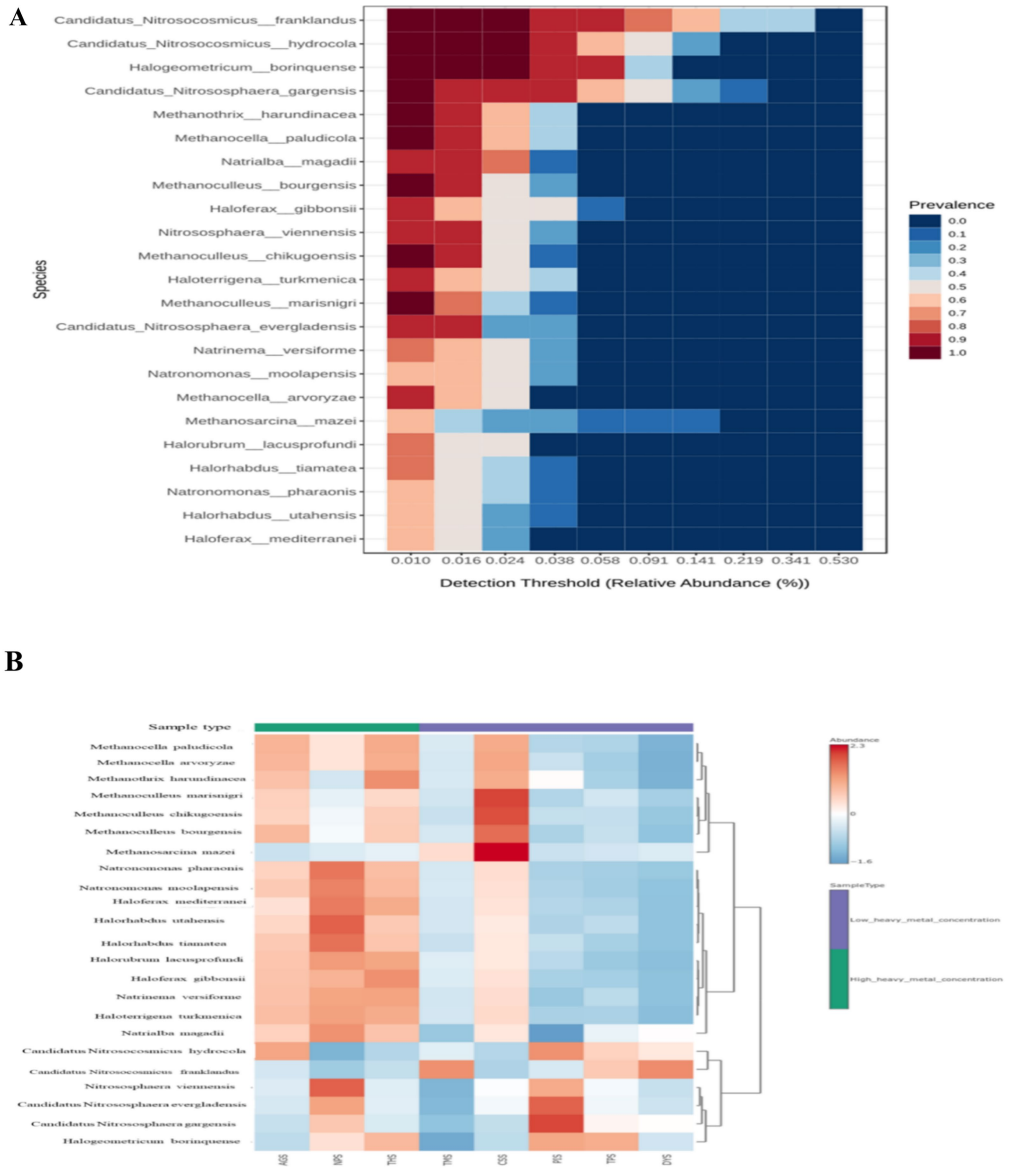
**(A)** Heat map illustrating the core archaeal microbiome abundance; **(B)** Heat map illustrating the abundance of selected archaeal species across all the samples by using Euclidean distance measure and Ward clustering algorithm. According to the z-score scale, red indicates a high abundance, whereas blue indicates a low abundance.

The representations of alpha diversity indices across the samples can be seen in Supplementary Figure 7A. HHMC samples were having higher alpha diversity than the LHMC samples. Among the LHMC samples CSS had the highest alpha diversity values similar as the HHMC samples. DYS sample had the lowest alpha diversity. The significant *p*-values (≤ 0.05) for Simpson (0.04) and Shannon (0.02) alpha diversity indices indicate that bacterial ecological niches differ between ‘high heavy metal concentration’ (HHMC) and ‘low heavy metal concentration’ (LHMC) samples. The PCA score plot of the first two principal components clearly shows that high and low concentrations of heavy metals are the primary basis for separation, where 99.1 and 0.6% of the variations were explained by PC1 and PC2, respectively (Supplementary Figure 7B). No significant inter-group variations existed between the HHMC and LHMC sample groups on the basis of archaeal beta diversity (Supplementary Figure 7C). High-heavy metal concentration samples, such as AGS and THS, were well separated from low-heavy metal concentration samples, except NPS, where the NPS sample overlapped with Low-heavy metal concentration samples (CSS, DYS, TMS, TPS, and PIS) (Supplementary Figure 7B). Euryarchaeota dominated the HHMC and LHMC samples at the phylum level, followed by Thaumarchaeota. Euryarchaeota was more abundant in HHMC (82%) than in LHMC (70.4%). Moreover, Thaumarchaeota was more abundant in LHMC (26.1%) than in HHMC (15.4%). Thaumarchaeota was found to be more abundant in DYS, PIS, TMS, and TPS among the LHMC samples. Compared with the LHMC samples, the HHMC samples (especially the AGS and THS) presented a greater abundance of archaea (Supplementary Figure 8A). *Candidatus Nitrosocosmicus* was the most abundant taxon, followed by *Nitrososphaera*, *Methanoculleus, Halogeometricum and Methanocella* at the genus level (Supplementary Figure 8B). *Candidatus Nitrosocosmicus franklandus* was the most abundant taxon, followed by *Candidatus Nitrosocosmicus hydrocola*, *Candidatus Nitrososphaera gargensis, Halogeometricum borinquense,* and *Haloferax gibbonsii* at the species level (Supplementary Figure 8C). *Candidatus Nitrosocosmicus gargensis* and *Candidatus Nitrosocosmicus evergladensis* were abundant in PIS, and *Candidatus Nitrosocosmicus franklandus* was abundant in DYS, with a prevalence of 53.03% (Supplementary Figure 8C). Twenty-three species were identified as the core microbiome. *Candidatus Nitrosocosmicus franklandus* was the most dominant species in the core microbiome, followed by *Candidatus Nitrosocosmicus hydrocola, Halogeometricum borinquense and Candidatus Nitrososphaera gargensis* (Figure 4A). AGS had relatively higher prevalence of *Candidatus Nitrosocosmicus hydrocola, Methanocella paludicola,* and *Methanocella arvoryzae*. NPS had high abundances of *Nitrososphaera viennensis, Halorhabdus utahensis,* and *Natronomonas pharaonis*. THS had high abundances of *Haloferax gibbonsii* and *Methanothrix harundinaceae*. Among the LHMC samples, CSS had a relatively high prevalence of *Methanosarcina mazei, Methanoculleus marisnigri,* and *Methanoculleus chikugoensis* (Figure 4B). Most of the archaeal genera were positively correlated with each other, mainly *Methanopyrus, Haloferax, Halosimplex, Halomicrobium, Halobaculum, Natrinema, Haloterrigena,* and *Halorhabdus*. Moreover, *Methanobacterium* was negatively correlated with many bacteria, such as *Methanopyrus, Haloferax, Halosimplex, Halomicrobium, Halobaculum, Natrinema, Haloterrigena,* and *Halorabdus* (Supplementary Figure 9).

The ammonia-oxidizing archaea (AOA) of the phylum Thaumarchaeota are a diverse, ubiquitous, and crucial functional group of microbes found in a variety of environments. *Candidatus Nitrososphaera gargensis* has adjusts to its niche in a heavy metal-containing thermal spring through an abundance of heavy metal resistance genes, chaperones, and mannosylglycerate as an appropriate substance. It has the genetic capacity to adapt to ecological alterations by communicating via a significant number of two-component systems, by chemotaxis and flagellum-mediated movement, and even by gas vacuole development (Spang et al., 2012). *Nitrosocosmicus franklandus* is an environmentally significant strain of Thaumarchaeota that has the capacity to interact well with ammonium oxidizing bacteria in enriched soils that have elevated ammonium levels (Lehtovirta-Morley et al., 2016). The *Haloferax gibbonsii* strain contains essential enzymes for the manufacture of the bioplastic polyhydroxyalanoate (Pinto et al., 2015).

### 3.7 Fungal diversityacross the soil samples

**Figure 5:**
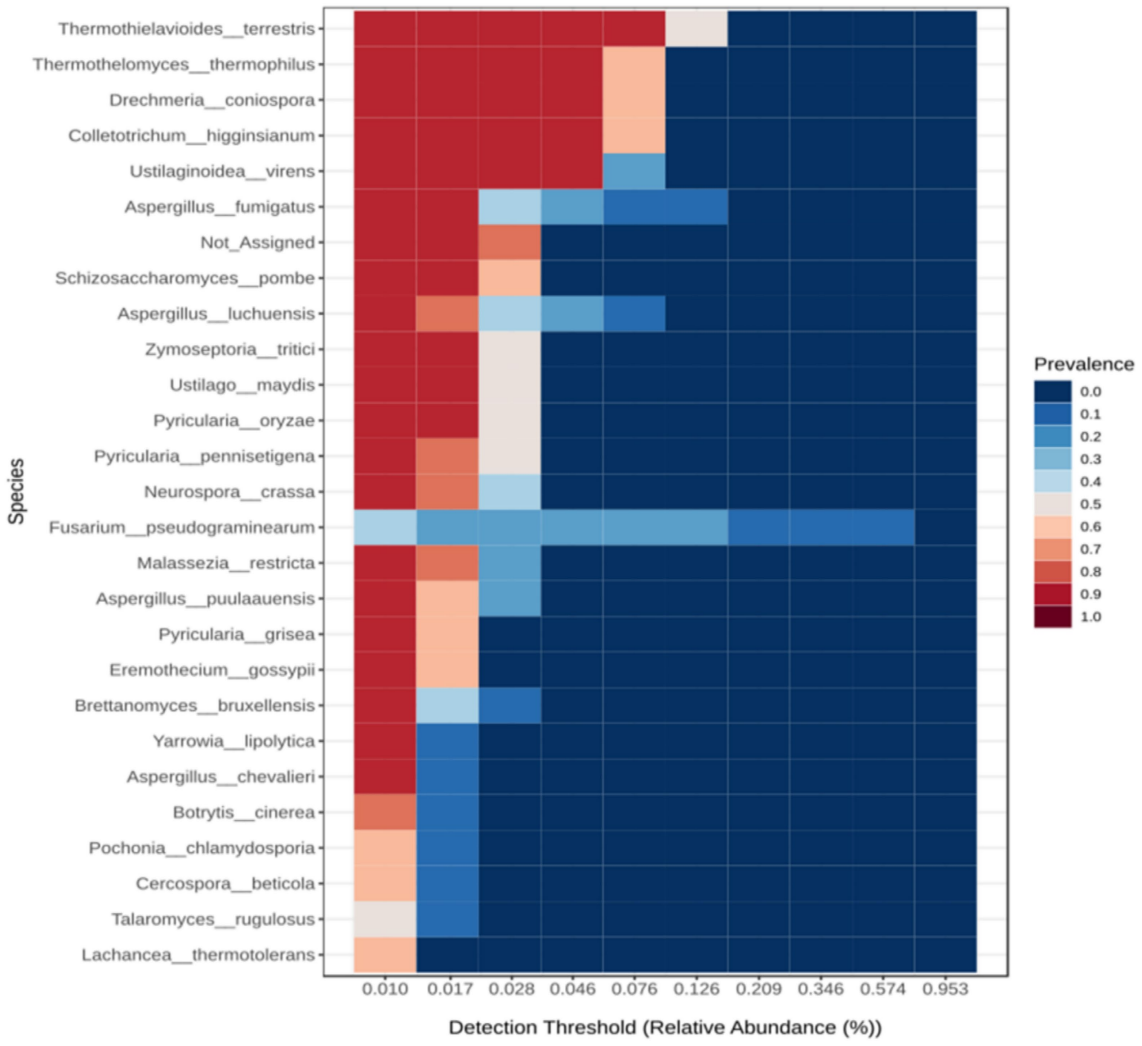
Heat map illustrating the core fungal microbiome abundance using Microbiome Analyst 2.0. The core microbiome refers to the set of taxa that are detected in a high fraction of the population above a given abundance threshold. A sample prevalence of 20% and a relative abundance of 0.01% were set as the cut-off values.

The stacked bar plot shows that the HHMC samples have a greater abundance of fungi than the LHMC samples, especially the AGS samples. The stacked bar plot (Supplementary Figure 10A) shows that Ascomycota was highly dominant across the samples, followed by Basidiomycota. Ascomycota was most abundant in the AGS sample. *Fusarium pseudograminearum* was the most abundant taxon, followed by *Thermothielavioides terrestris*, *Colletotrichum higginsianum,* and *Drechmeria coniospora*. *Fusarium pseudograminearum* was the most abundant species in the AGS samples with a prevalence of 95.26% (Supplementary Figure 10B). The Heatmap (Supplementary Figure 10C) depicts the species-level fungal abundance across the samples. Among the HHMC samples, the AGS samples presenteda relatively high abundance of *Fusarium pseudograminearum*. *Aspergillus chevalieri, Talaromyces rugulosus, Marasmium ordeaes,* and *Aspergillus puulaauensis* were prevalent in the NPS samples. In THS, Pyricularia *oryzae* was abundant. TMS revealed high Levelsof*cryptococcus neoformans, Cercospora beticola,* and *Malassezia restricta*in LHMC samples. O*gataea parapolymorpha, Pyricularia pennisetigena,* and *Pyricularia oryzae* were abundant in the CSS sample. The PIS sample presented greater abundances of *Ustilaginoidea virens, Brettanomyces bruxellensis, Ustilago maydis,* and *Fusarium venenatum*. TheTPS sample presented higher abundances of *Pochonia chlamydosporia* and *Botrytis cinere*. The DYS sample presented high abundances of *Aspergillus fumigatus, Aspergillus luchuensis, and Aspergillus puulaauensis*. Twenty-six species were identified in the core microbiome (Figure 5), among which *Thermothielavioides terrestris* was the predominant species, followed by *Thermothelomyces thermophilus, Drechmeria coniospora and Colletotrichum higginsianum*.

Aside from a considerable drop in wheat production, *F. pseudograminearum* produces a wide range of harmful secondary metabolites, particularly mycotoxins. Cereals infected with mycotoxins are unfit for consumption or feeding. *Fusarium* produces a variety of mycotoxins, including trichothecenes and fumonisins. The most prevalent Fusarium mycotoxin is deoxynivalenol (DON), which leads to vomiting, oral infections, skin irritation, and bleeding in humans and livestock (Li et al., 2022). *T. terrestris* contains enzymes including cellulases and hemicellulases that can degrade polysaccharides from biomass, contributing to the global carbon cycle (Camargo et al., 2020). *C. higginsianum* is a hemibiotrophic ascomycete fungus that leads to commercially significant anthracnose infections in a variety of monocot and dicot plants around the world (Yan et al., 2018). *Drechmeria coniospora* is an obligate parasitic fungus from the Clavicipitaceae family. It affects a wide spectrum of nematode species by producing spores that cling to their cuticles (Wan et al., 2021). *C. neoformans* is a dimorphic fungus leading to fatal meningoencephalitis primarily in immunocompromised people (Zhao and Lin, 2021). Cercospora leaf spot, resulting from the fungal pathogen *C. beticola*, is a potentially devastating foliar disease of sugar beets globally (Rangel et al., 2020). *M. restricta* is an opportunistic fungal pathogen associated with skin disorders such as atopic dermatitis, seborrheic dermatitis, and psoriasis (Peng et al., 2024). *P. pennisetigena* is pathogenic to Poaceae plants in Brazil, China, Japan, Philippines, and United States (Yi et al., 2022). *U. virens* is responsible for the development ofdevastating Rice false smut disease across the globe (Chen et al., 2024). *B. bruxellensis* is found not only in wine but also in several different kinds of alcohol. These yeasts can withstand the extreme circumstances encountered throughout the fermenting process, such as increasing ethanol levels and increasing the amount ofsulphur dioxide administered (Smith and Divol, 2016). *U. maydis* is a biotrophic fungal pathogen that produces tumours throughout all airborne maize tissues (Ferris and Walbot, 2020). *B. cinerea*, a common plant pathogen with a necrotrophic way of life, produces grey mould infection on numerous plants (Bi et al., 2023). *Aspergillus fumigatus* is an ecological filamentous fungus that can cause serious illnesses in immunocompromised people (van de Veerdonk et al., 2017). *Thermothelomyces thermophilus* is a thermophilic ascomycete that produces several glycoside hydrolases and oxidative enzymes that aid in the degradation of lignocellulosic materials (Contato et al., 2024).

### 3.8 Protozoan diversity across the soil samples

**Figure 6:**
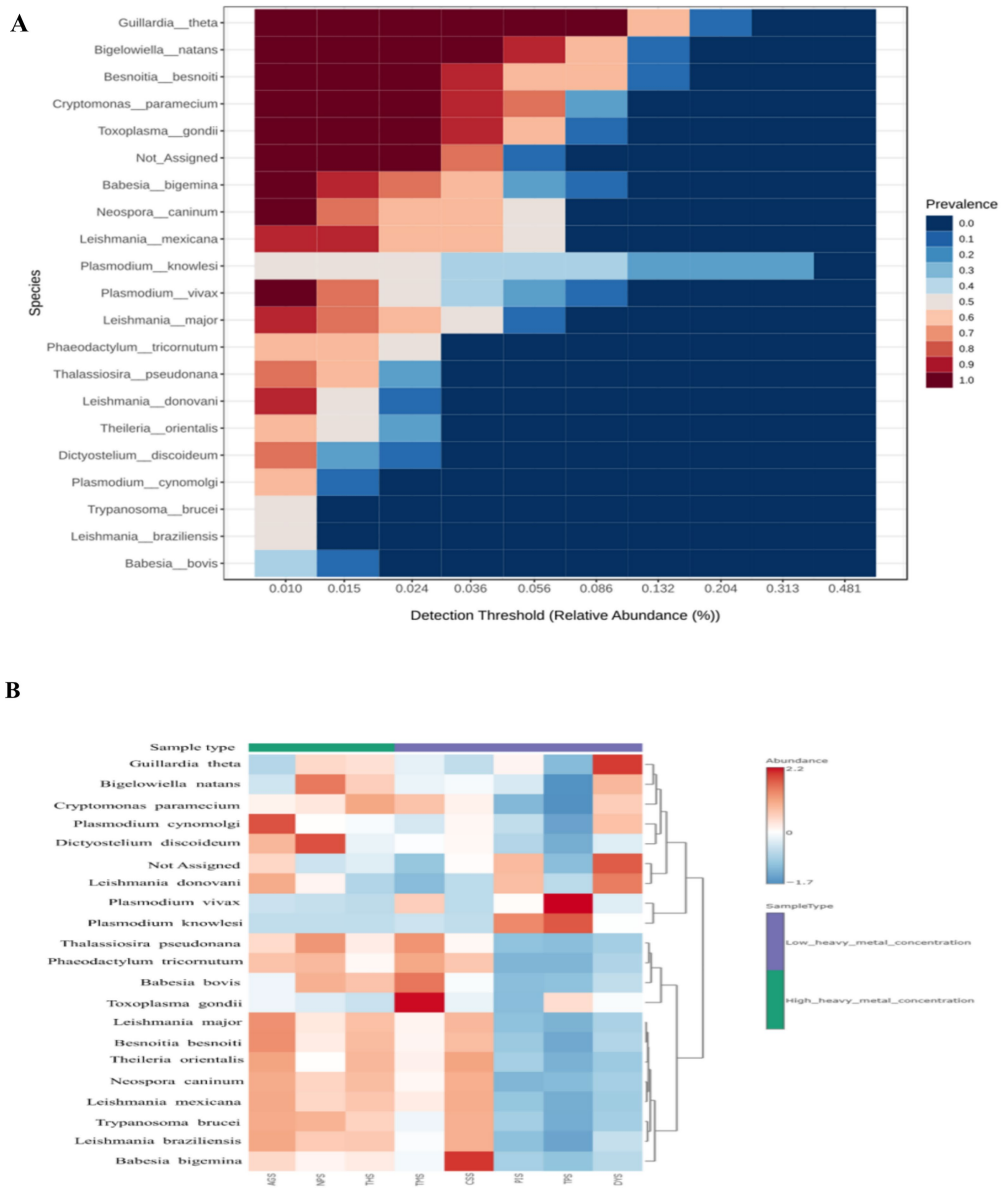
**(A)** The core microbiome of the protozoan taxa across the soil samples. A sample prevalence of 20% and a relative abundance of 0.01% were set as the cut-off values at the Species level. **(B)** Heat map illustrating species-level protozoan abundances across the samples.

The stacked bar plot shows that the HHMC samples had greater protozoa diversity, followed by the LHMC samples (Supplementary Figure 11A). According to the stack bar plot (Supplementary Figure 11A), Apicomplexa was the most abundant species in the HHMC and LHMC samples, followed by Unclassified Protozoa and Euglenozoa. Among the genera, *Leishmania* was the most abundant followed the *Gaillardia, Besnoitia, Bigelowiella* and *Plasmodium*. Moreover, at the species level, *Guillardia theta* was predominant, followed by *Besnoitia besnoiti, Bigelowiella natans, Cryptomonas paramecium and Toxoplasma gondi* (Supplementary Figure 11B). Figure 6A shows that at the protozoan species level, *Gaillardia theta, Bigelowiella natans, Besnoitia besnoiti, Cryptomonas paramecium* and *Toxoplasma gondi* were the prevalent core microbiome taxa. Among the HHMC samples, the AGS sample presented greater abundances of *Plasmodium cynomolgi, Leishmania donovani, Leishmania major,* and *Besnoitia besnoiti*. NPS had a high prevalence of *Bigelowiella natans, Dictyostelium discoideum,* and *Thalassiosira pseudonana*. THS had a high prevalence of *Cryptomonas paramecium*. Among the LHMC samples, TMS had a greater prevalence of *Toxoplasma gondi*, *Babesia bovis*, and *Thalassiosira pseudonana*. In contrast, CSS had a greater abundance of *Babesia bigemina,* and PIS had a higher abundance of *Plasmodium knowlesi*. *Plasmodium vivax, and Plasmodium knowlesi* were predominant in the TPS samples. DYS had a relatively high abundance of *Gaillardia theta and Leishmania donovani* (Figure 6B). Most protozoan species were positively correlated with each other with the exception of*Plasmodium knowlesi*. *Plasmodium knowlesi*was negatively correlated with many protozoa, mainly *Besnoitia besnoiti, Theileria orientalis, Leishmania major, Trypanosoma brucei, Neospora caninum, Leishmania panamensis, Phaeodactylum tricomutum, and Dictyostelium discoideum*. *Plasmodium knowlesi* was positively correlated with only *Plasmodium vivax* (Supplementary Figure 12).

Bovine besnoitiosis can be caused by the parasite *B. besnoiti*, a cyst-forming apicomplexan parasite that is related to *Toxoplasma gondii* and *Neospora caninum* (Zhou et al., 2020). *Toxoplasma gondii* infections are widespread in humans and animals globally. Rodents are among the most significant secondary hosts for *T. gondii* because they are preyed upon by cats, which then deposit environmentally tolerant oocysts in their stool, transmitting the infection (Dubey et al., 2021). *Neospora caninum*, a parasite that forms tissue cysts, is the cause of bovine neosporosis. It is considered as one of the major causesof reproductive dysfunction in cattle; abortion and death of newborns result in considerable economic losses in the global cattle sector (Marugan-Hernandez, 2017). *P. knowlesi*are transferred between humans and wild macaques via mosquito vectors (Naik, 2020). *P. vivax* causes the vast majority of outbreaks of malaria in Asia and the Americas (Adams and Mueller, 2017). *T. orientalis* is the cause of benign or non-transforming theileriosis, and has a principal impact via erythrocyte degradation. *T. orientalis* is an economically significant pathogen of cattle in Australia, New Zealand, and Japan, particularly when young animals are transplanted into a native environment (Watts et al., 2016). *L. donovani*, a kinetoplastid protozoan, is the second most significant parasite and the root cause of life-threatening visceral leishmaniasis (Paul et al., 2023).

### 3.9 Correlations between microbial compositions, soil physiochemical properties, and contaminants

**Figure 7:**
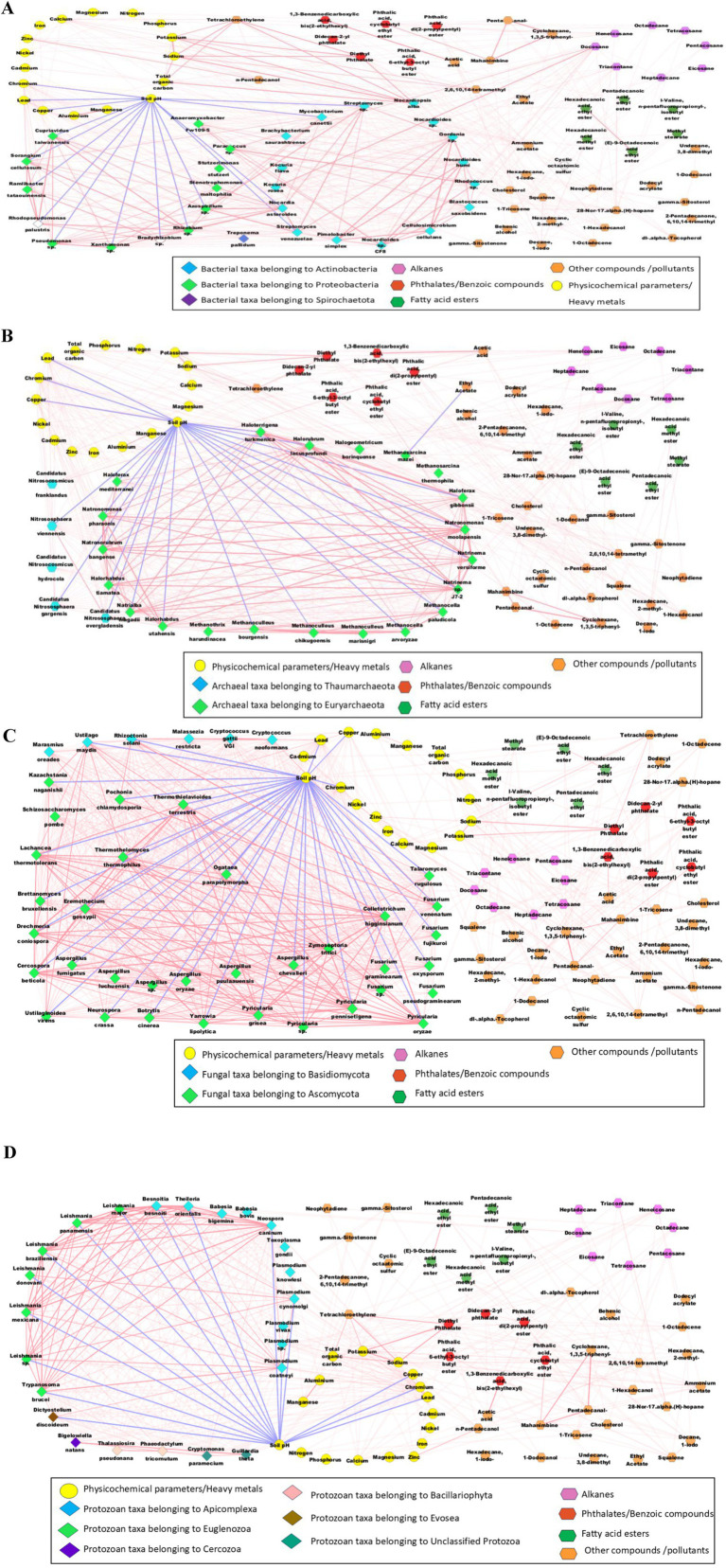
Network analysis depicting the Pearson correlations between the bacterial taxa **(A)**, archaeal taxa **(B)**, fungal taxa **(C)**, protozoan taxa **(D)**, soil physicochemical characteristics, heavy metal concentrations, pollutants and other metabolites/compounds. All the blue edges represent negative correlations, and pink edges represent positive correlations (Pearson correlation coefficient values higher than 0.75 and lower than −0.75 were selectedto generate the correlation network in Cytoscape v3.10.2.with the Metscape plugin).

The correlation plots (Figure 7) were constructed using the statistically significant interactions between the microbial taxa, soil physiochemical characteristics, heavy metal concentrations, pollutants, metabolites, and other compounds using the Metscape plugin in Cytoscape v3.10.2 software (based on the Pearson correlation coefficient (r) values >0.75 and <−0.75; *p*-value <0.05). Positive inter- and intra-phyla correlations were observed across bacteria, fungi, archaea, and protozoan taxa. Na, K, TOC, diethyl phthalate and Tetrachloroethylene were positively correlated with one another. The soil pH was negatively correlated with most microbial taxa. Soil pH plays a significant role in determining the microbiome compositions of forest soils (Bowd et al., 2022) and the fungal compositions of wastewater treatment plants (Assress et al., 2019). Soil pH was also negatively correlated with Proteobacteria in sugarcane rhizosphere soils with high Mn concentrations (Li et al., 2023). Additionally, both the Proteobacteria and Actinobacteria detected in the red soils were negatively affected by soil pH (Muneer et al., 2022). Similarly, soil pH is a crucial factor in shaping the archaeal community compositionin the black soils of China, where the Thaumarchaeota and Euryarchaeota taxa are significantly affected by soil pH (Liu et al., 2019). The archaeal species *Methanosarcina mazei* and *Methanoculleus marisnigri*, as well as the fungal species *Cryptococcus neoformans* were also abundant in the sediment samples from the polluted stretches of the river Ganga in India (Rout et al., 2022). Pb, Cr, Cd, Ni, Cu, Zn and Fe were positively correlated with the bacteria, archaea, fungi, protozoa, and virus taxa. However, several edges with strong correlations (Pearson correlation coefficient values >0.75 and <−0.75) were detected for Pb, Cr, Cd, and Cu. Higher concentrations of Mn reduce the abundance of soil bacteria, particularly Proteobacteria and Actinobacteria, which are negatively correlated with Mn concentrations.

Research suggests that high concentrations of heavy metals can reduce sediment bacterial biomass, whereas low amounts can increase the biomass of bacteria and promote growth (Chen et al., 2018). Proteobacteria have been found to be the dominant bacteria in soil over time after exposure to heavy metals, since they are more resistant to the presence of heavy metals. According to Schneider et al. (2017) and Zhao et al. (2019a), there is a positive association between Proteobacteria and heavy metal levels.

Among the bacterial taxa (Figure 7A), Actinobacteria and Proteobacteria showed significant positive correlations within their respective intra-phyla taxa. Moreover, Proteobacteria taxa, such as *Azospirillum* sp., *Rhizobium* sp., and *Bradyrhizobium* sp., had strong positive correlations with several Actinobacteria taxa such as *Nocardia, Nocardiodes, Streptomyces* and *Rhodococcus* species. These results are in agreement with the observations of Yan et al. (2021), where Actinobacteria taxa were found to exhibit the least competitive interactions with Proteobacteria taxa (especially under oligotrophic conditions), probably due to fewer niche overlap effects between these phyla. Within the Actinobacteria phylum, *Nocardioides humi, Nocardioides* sp. CF8, *Cellulosimicrobium cellulans, Gordonia* sp., and *Pimelobacter simplex* were strongly positively correlated with each other. Moreover, *Nocardia asteroids* and *Gordonia* sp. had positive correlations with *Streptomyces* sp. and *Streptomyces venezuelae*. Similarly, among the Proteobacteria taxa, *Azospirillum* sp., *Ramlibactertataouinensis, Rhizobium* sp., *Xanthomonas* sp., and *Sorangium cellulosum* were positively correlated with one another. *Nocardiopsis alba* had strong positive correlations with Na (*r* = 0.99, *p*-value = 0.000000096), K (*r* = 0.98, *p*-value = 0.00048), TOC (*r* = 0.98, *p*-value = 0.000005), diethyl phthalate (*r* = 0.99, *p*-value = 0.000002) and tetrachloroethylene (*r* = 0.99, *p*-value = 0.000001). *Nocardiopsis alba* can solubilise potassium (Boubekri et al., 2021). The K solubilising ability of *Nocardiopsis alba* may have led to a positive correlation with K. Additionally, *Nocardiopsis* plays a crucial role in organic decomposition and lignocellulose degradation (Wang et al., 2025), probably leading to a positive correlation with TOC. The alkane compound docosane was strongly positively correlated with *Paracoccus* sp. (*r* = 0.97, *p*-value = 0.00006) and *Kocuria rosea* (*r* = 0.99, *p*-value = 0.000001). A *Kocuria rosea* strain with biosurfactant properties was identified from an oil refinery (Akbari et al., 2021), and the oil-degrading capabilities of *Paracoccus* strains were reported (Lyu et al., 2022). Moreover, docosane is a significant petroleum pollutant (Alsharyani and Muruganandam, 2024). Hence, the availability of docosane may have increased the abundance of *Kocuria rosea* and *Paracoccus* sp., which maybe indicated by these positive correlations. *Nocardiopsis alba* had strong positive correlations with diethyl phthalate (*r* = 0.99, *p*-value = 0.000002) and tetrachloroethylene (*r* = 0.99, *p*-value = 0.000001). *Nocardiopsis alba* strains are known to degrade several complex pollutants, including naphthalene compounds, into their derivatives, such as phthalic acids and acetic acids (Doley et al., 2017). These compounds (diethyl phthalate and tetrachloroethylene) may be the byproducts of complex substances degraded by *Nocardiopsis alba*. *Z*n (*r* = 0.76, *p*-value = 0.029) was also positively correlated with *Nocardiopsis alba*. Heavy metals such as Pb, Cr, Cd, and Cu had significant positive correlations with most bacterial taxa. Pb had the strongest positive correlation with *Streptomyces* sp. (*r* = 0.91, *p*-value = 0.0014), followed by *Mycobacterium canettii* (*r* = 0.89, *p*-value = 0.003) and *Pseudomonas* sp. (*r* = 0.86, *p*-value = 0.0063). Moreover, Cu had the strongest positive correlation with *Stutzerimonas stutzeri* (*r* = 0.79, *p*-value = 0.017), followed by *Xanthomonas* sp. (*r* = 0.79, *p*-value = 0.017), and *Pseudomonas* sp. (*r* = 0.79, *p*-value = 0.0208). Similarly, Cr was correlated with *Xanthomonas* sp. (*r* = 0.96, *p*-value = 0.00012), and *Pseudomonas* sp. (*r* = 0.96, *p*-value = 0.000094). Moreover, Cd had significant positive correlations with *Stutzerimonas stutzeri* (*r* = 0.91, *p*-value = 0.0017) and *Nocardiopsis alba* (*r* = 0.75, *p*-value = 0.03). Earlier studies reported that heavy metal-tolerant bacterial species, namely, *Streptomyces* (Elnahas et al., 2021), *Pseudomonas* (Vélez et al., 2021), and *Xanthomonas* (Ramnarine et al., 2024) can remove or sequester toxic heavy metals in contaminated environments. The heavy metal tolerance of these bacteria may have led to positive correlations with Pb, Cr, Cd and Cu. Heptadecane, Triacontane, Docosane and Heneicosane were the alkanes that had significant positive correlations with bacterial taxa. Henicosane had a significant positive correlation with *Nocardiopsis alba* (*r* = 0.86, *p*-value = 0.006). Moreover, heptadecane had strong positive correlations with *Kocuria flava* (*r* = 0.84, *p*-value = 0.0075) and *Kocuria rosea* (*r* = 0.85, *p*-value = 0.007). However, docosane and triacontane had high numbers of significant correlations with bacterial taxa, especially with *Nocardioides* sp., *Nocardioides* sp. CF*8, Nocardioides humi, Kocuria flava, Kocuria rosea, Rhodococcus* sp., *Paracoccus* sp., *Blastococcussaxobsidens,* and *Anaeromyxobacter* sp. *Fw109-5*. Docosane had the strongest positive correlation with *Kocuria rosea* (*r* = 0.99, *p*-value = 0.000001), followed by *Paracoccus* sp. (*r* = 0.97, *p*-value = 0.00006), *Kocuria flava* (*r* = 0.96, *p*-value = 0.00013) and *Rhodococcus* sp. (*r* = 0.97, *p*-value = 0.0001). Moreover, *Rhodococcus* species are known for their ability to biodegrade and adhereto hydrocarbon compounds (Ivshina et al., 2022), and are reported to be present in various bacterial consortia, which are capable of biodegrading of various environmental pollutants, including pyrene, butane, and phthalates (Zhang and Zhang, 2022). Additionally, *Kocuria flava* and *Rhodococcus* consortia are effective at biodegrading various polycyclic aromatic hydrocarbons (Sakshi Singh et al., 2022). Moreover, *Nocardioides* species can degrade pollutants, including hydrocarbons and aromatic compounds (Ma et al., 2023) and *Nocardioides* sp. *CF8* was reported to be alkane utilizing bacterial taxon (Hamamura and Arp, 2000). Hence, the alkane-degrading and alkane-utilizing capabilities of these bacterial species may have led to the significant positive correlations between these bacteria and the above-mentioned alkane compounds.

The positive correlations between the taxa imply the co-occurrence of the taxa due to cooperative interactions or similar niche preferences. In contrast, their negative correlations imply the co-exclusion of the taxa probably due to competition (Ju and Zhang, 2015). The study explores a variety of positive interactions among different species, including cooperative interactions like mutualism and synergism that support involved taxa. Symbiosis, cross-feeding, and consortia formation are the primary mechanisms through which bacteria form these positive associations (Weiland-Bräuer, 2021), and these microbe-microbe interactions can contribute to soil-health promotion activities, including biodegradation and pollution alleviation (Wu et al., 2023). For example, *Mycobacterium, Rhodococcus*, Rhizobiales and Proteobacteria taxa are reported to be present in various bacterial consortia, and are capable of biodegrading of various environmental pollutants, including phthalic acid esters, pyrene, butane, and other phthalates (Zhang and Zhang, 2022). Additionally, the *Kocuria flava* and *Rhodococcus* consortium were effective in biodegradation of various polycyclic aromatic hydrocarbons (Sakshi Singh et al., 2022). Hence, the intra-phyla positive correlations between the taxa detected in the present study may have arisen because positive associations between microbes and the removal of various pollutants from soils and improve soil health.

The archaeal phyla (Figure 7B) had no significant inter-phyla correlations between Thaumarchaeota and Euryarchaeota. On the other hand, strong positive intra-phyla correlations were observed, especially among the Euryarchaeota taxa, namely, *Haloterrigena turkmenica, Halorubrum lacusprofundi, Haloferax gibbonsii, Natronomonas moolapensis, Natronomonas pharaonis, Natrinema* sp. *J7-2, Natrinema versiforme, Halorhabdus tiamatea*, and *Halorhabdus utahensis*. Thaumarchaeota and Euryarchaeota were the major archaeal phyla detected across various Amazonian soil samples. They had greater positive correlations (50–80%) between the taxa, probably due to syntrophic interactions between the archaeal taxa (de Chaves et al., 2022).

*Methanosarcina mazei* had a significant negative correlation with ethyl acetate (*r* = −0.76, *p*-value = 0.0263). *Methanosarcina mazei* is an aceticlastic methanogen that can convert acetate into CO_2_ and CH_4_ (Welte et al., 2010). Hence, the negative correlation may have occurred due to the increased conversion of ethyl acetate into CO_2_ and CH_4_ in the soils with a relatively higher abundance of *Methanosarcina mazei*. Pb, Cr, Cd, and Cu had significant positive correlations with most archaeal taxa. *Candidatus Nitrososphaera evergladensis, Halorhabdus utahensis, Natronomonas pharaonis,* and *Nitrososphaera viennensis* presented strong positive correlations with Pb, Cr, Cd, and Cu. In contrast, *Candidatus Nitrososphaera gargensis, Halogeometricum borinquense, Haloferax gibbonsii, Haloterrigena turkmenica, Natrialba magadii, Natronomonas moolapensis, Natrinema versiforme, Halorubrum lacusprofundi, Halorhabdus tiamatea, Natronorubrum bangense, Natrinema* sp. *J7-2,* and *Haloferax mediterranei* were positively correlated with Pb, Cr, and Cu, but not with Cd. Docosane was the only alkane to possess significant positive correlations with the archaeal taxa, especially with *Halogeometricum borinquense* (*r* = 0.092, *p*-value = 0.001)*, Haloferax gibbonsii* (*r* = 0.84, *p*-value = 0.009)*, Methanocella paludicola* (*r* = 0.78, *p*-value = 0.02)*, Methanothrix harundinacea* (*r* = 0.84, *p*-value = 0.009)*, Natrinema versiforme* (*r* = 0.8, *p*-value = 0.0167)*, Halorubrum lacusprofundi* (*r* = 0.8, *p*-value = 0.017) and *Haloferax mediterranei* (*r* = 0.82, *p*-value = 0.013). Halophilic archaea, including *Haloferax* species, were previously reported to exhibit hydrocarbon-degrading properties (Park and Park, 2018).

Significant positive correlations were detected among the Ascomycota and Basidiomycota taxa (Figure 7C). Among the Ascomycota taxa, *Drechmeria coniospora, Thermothielavioides terrestris, Thermothelomyces thermophilus, Cercospora beticola, Zymoseptoria tritici, Fusarium and Pyricularia species* presented strong positive correlations. *Ustilago maydis, Rhizoctonia solani,* and *Cryptococcus neoformans* were the major Basidiomycota taxa that were positively correlated with the Ascomycota species (especially, the *Fusarium* and *Pyricularia* species). Ascomycota and Basidiomycota were the most dominant phyla detected, with many interconnections in the co-occurrence networks of the mycobiome of wastewater treatment plants, indicating the importance and adaptive capabilities of these phyla (Assress et al., 2019). The co-occurrence of some members of Ascomycota and Basidiomycota may be due to their co-evolutionary relationships, as in the case of *Wynnea* (Ascomycota) and *Armillaria* (Basidiomycota) (Xu et al., 2019). Pb and Cr had significant positive correlations with all the selected fungal taxa, except for *Fusarium pseudograminearum*. Moreover, *Marasmius oreades, Aspergillus* sp., and *Aspergillusoryzae* had strong positive correlations with Pb, Cr, Cd and Cu. *Aspergillus* and *Fusarium* were reported to possess heavy metal tolerance properties (especially to Cr and Cu), and were considered potential bioremediation agents (Amin et al., 2024). Four fungal taxa namely, *Pyricularia oryzae* (*r* = 0.75, *p*-value = 0.03), *Pyricularia* sp. (*r* = 0.76, *p*-value = 0.026), *Schizosaccharomyces pombe* (*r* = 0.84, *p*-value = 0.0089), and *Brettanomyces bruxellensis* (*r* = 0.81, *p*-value = 0.016) were significantly positively correlated with docosane.

Apicomplexa and Euglenozoa were the protozoan phyla with the most positive inter- and intra-phyla correlations (Figure 7D). The Apicomplexa taxa, such as *Plasmodium cynomolgi, Plasmodium coatneyi species, Neospora caninum, Theileria orientalis,* and *Besnoitia besnoiti,* had significant positive correlations with the Euglenozoa taxa, such as *Trypanosoma brucei* and the *Leishmania* species. The greater positive correlations among the protozoan taxa may indicate enhanced cooperative interactions, which may be an adaptive measure used by the protozoans through expanding the niche to ensure survival under stressful conditions (Hu et al., 2024). *Bigelowiella natans* had significant positive correlations with *Guillardia theta* and *Cryptomonas paramecium*. *Dictyostelium discoideum* was significantly positively correlated with Pb, Cr, Cu and Cd. Several protozoan taxa, such as *Guillardia theta, Bigelowiella natans, Thalassiosira pseudonana, Leishmania donovani, Leishmania braziliensis,* and *Plasmodium coatneyi* were significant positively correlated with Pb, Cr, and Cu, but not with Cd. Moreover, docosane was positively correlated with *Guillardia theta* (*r* = 0.87, *p*-value = 0.0046), *Bigelowiella natans* (*r* = 0.82, *p*-value = 0.012)*, Cryptomonas paramecium* (*r* = 0.84, *p*-value = 0.009), and *Babesia bovis* (*r* = 0.78, *p*-value = 0.02). Previous studies suggest that protozoan taxa and hydrocarbon correlations may occur due to protozoan predation. i.e., protozoa may indirectly affect hydrocarbon degradation through the differential predation of bacteria or fungi (Du et al., 2024). Hence, the positive correlation with docosane may be due tothe indirect influence of protozoans. A significant negative correlation was observed between neophytidine and *Plasmodium vivax* (*r* = −0.72, *p*-value = 0.0425). Neophytidine is a plant metabolite reported to possess antimicrobial activity (Ngobeni et al., 2020), which may be a reason for its negative correlation with *P. vivax*. However, why the negative correlation only exists with *P. vivax* is unclear, and whether actual inhibition exists or whether a negative correlation occurrs through random chance can be answered only through a detailed study.

The study indicated positive relationships between bacteria, archaea, fungi, and protozoa implying that these interactions may promote pollutant degradation and improve soil health. The strong associations between particular bacteria and the presence of heavy metals and alkanes indicate their tolerance to these contaminants and highlight their potential for bioremediation; hence, facilitating soil restoration initiatives.

### 3.10 Determination and assembly of AMR genes

**Figure 8:**
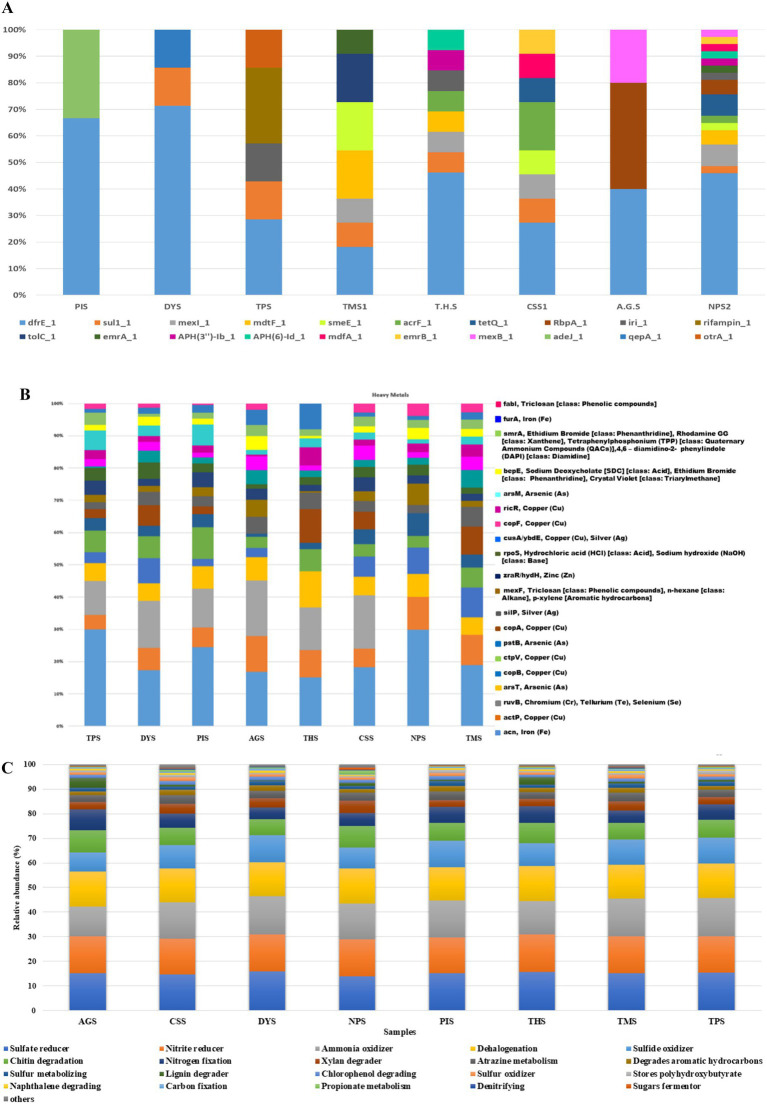
Comparison of relative abundance of antibiotic resistance genes **(A)** and heavy metal resistance genes **(B)** in soil samples of different sites. **(C)** Relative abundance of top 20 metabolic genes identified in soil samples of eight different areas.

A total of 320 resistance genes related to 30 antibiotics were identified by the comprehensive antibiotic resistance database annotation, and resistance genes related to diaminopyrimidine, sulfonamide, tetracycline, fluoroquinolone, rifamycin, and aminoglycoside were present in higher abundances than the other resistance genes. The bar plot (Figure 8A) depicts the major AMR genes detected across the samples. AMR genes were more common in NPS2, CSS1, T. H. S, TMS1, and TPS. The presence of the *dfrE_1* gene was reported in all the soil samples (Figure 8A). The *dfrE_1* and *sul_1*genes were the most common AMR genes. The *dfrE_1* gene was abundant in all the samples, and the *sul_1* gene was present in six samples, excluding PIS and AGS. The greatest number of AMR genes was detected in the NPS2 sample, followed by CSS and THS, and the lowest number of genes was detected in PIS and DYS. The HHMC samples presented more AMR genes, except the AGS samples. *dfrE_1, RbpA_1* and *mexB_1* were the AMR genes found in the AGS sample. The metagenomic study identified antibiotic resistance genes from eight polluted sites, each of which imparts resistance to various antibiotics. The highest percentage of multidrug-resistance genes were found in CSS (71%), followed by TMS (63%) and AGS (53%). Tetracycline, glycopeptides, rifampin and aminoglycoside were the other major ARGs. Tetracycline and glycopeptide-resistance genes were abundant in the TPS, PIS, and DYS soil samples. Rifampin-resistance genes were abundant in AGS (10%), THS (9%), CSS (8%), and TMS (7%) samples. Moreover, Aminoglycoside resistance genes were dominant in the THS and AGS samples (Supplementary Figure 13). A significant number of multidrug-resistance genes such as *MexD, MexC, MexE, MexF, MexT, CmeB*, MdtB, MdtC, and *OprN,* confer antibiotic resistance via efflux pumps in our study. The prevalence of drug-resistance genes differs among different sample resources. Antibiotic efflux, antibiotic inactivation, antibiotic target alteration, antibiotic target protection, and antibiotic target replacement are the main resistance mechanisms of ARGs identified in the present study. Among them, antibiotic efflux (42%) was the predominant mechanism, followed by antibiotic inactivation (23%) and antibiotic target alteration (18%) (Supplementary Figure 14). *Kocuria flava* and *Kocuria rosea* had significant positive correlations with the AMR genes *catB3_1, catQ_1,* and *aadA11_1,* and these three AMR genes were also positively correlated with each other. At the same time, *macB_1* was positively correlated with *Treponema pallidum* (*r* = 1, *p*-value = 7.1581e-24) (Supplementary Figure 15).

This study has limitations. First, this study did not include ongoing surveillance to assess ARG persistence with their bacterial hosts. Second, the study lacked antibiotic residue detection. As a result, more research is needed to determine the relationship between antibiotic residues and ARG diversity. Third, the environmental samples were insufficiently broad, with no samples from human beings, drinking water, dust, flies, or aerosols being collected. As a result, more research is needed into the potential exposure dangers of ARGs in duck farms.

ARGs associated with influential antibiotic efflux include mexC, and mexE which target fluoroquinolones, whereas those engaged in antibiotic target substitution and protection include RbpA, which target rifamycins. The ARG RbpA alters the association between rifampin and the *σ* factor of bacterial RNAP, causing an allosteric change in the rifampin binding pocket within the β component. The RbpA protein interacts with rifampicin at the β subunit attachment location of RNAP. This reduces the sensitivity of RNAP torifampicin and prevents its traditional suppression of the β subunit. Alterations in the β subunit conformation alter rifampin’s ability to attach to the enzyme, resulting in resistance (Jalal and Sonbol, 2024). ARGs and ARBs from livestock can spread to people via food, water, or air (Bai et al., 2024).

Li et al. (2015b) used metagenomic analysis to identify the presence of antibiotic genes in various soil samples. This study revealed a robust correlation between anthropogenic impacts and the abundance of total ARGs in ecosystems. In high-impact settings, ARG abundances increased by as much as three orders of magnitude compared with those in less-impacted environments. A link between ARGs and MGEs has been discovered in conserved urban and suburban environments (Zhao et al., 2019b). Hemala et al. (2014) identified a link between antimicrobial resistance and petroleum exposure. Hydrocarbon-degrading Gammaproteobacteria and Actinobacteria carry several antibiotic resistance genes. Antibiotics can impact the microbial population structure, selection of resistant microbes, and bacterial physiology through agricultural operations such as livestock manure usage, aquaculture, and the use of untreated effluent, which can contaminate soil and water resources (Kaviani Rad et al., 2022). Chen et al. (2017) reported resistance genes for aminoglycosides, cefoxitin, cefazolin, ceftriaxone, chloramphenicol, fluoroquinolones, penicillin, macrolides, tetracyclines, and polypeptides in a PAH-contaminated environment. The soil microbes isolated from urban garden plots are frequently resistant to ampicillin, cefoxitin, ceftriaxone, chloramphenicol, gentamicin, kanamycin, and penicillin (Mafiz et al., 2018). The resistome of the surroundings encompasses both innate and acquired resistance processes by bacteria. Fundamental resistance mechanisms include cellular responses to harmful compounds, such as wide-spectrum efflux pumps, chromosomally expressed resistant enzymes such as β-lactamases, and constraints that prevent entry such as porins and the outer membrane of gram-negative bacteria. Horizontal gene transfer (HGT) can result in acquired resistance mechanisms such as compound-specific efflux pumps, the expression of insensitive targets, and enzymes that alter structure and target of antibiotics (Das et al., 2021). Additionally, elevated levels of Cd in the areas nearest the industrial area resulted inincreasedlevelsof soil aminoglycoside, betalactam, fluoroquinolone, multidrug, and vancomycin resistance genes, but no similar stimulating influences were observed on different kinds of ARGs (Cheng et al., 2021).

Human-animal combined mobile ARGs have been found in human, chicken, pig, and cattle intestines, imparting resistance to six important antibiotic classes: tetracyclines, aminoglycosides, macrolide-lincosamide-streptogramin B (MLSB), chloramphenicols, β-lactams, and sulphonamides (Lawther et al., 2022). The prevalence of resistance genes to tetracycline (34.24%), aminoglycoside (19.37%), and macrolide (19.37%) is relatively high in chickens (Yang et al., 2022). Tetracycline resistance genes, including tet(Q)_1 and tet(W)_1, are present in all cattle microbiomes (Lawther et al., 2022). Proteobacteria is the major bacterial host and contains the greatest number of various ARGs in poultry birds (Liu et al., 2024). Tetracycline resistance genes such as *tet(Q)_1* and *tet(W)_1* were found in all the cattle microbiomes. Both of these tetracycline resistance genes have been found in key pathogenic bacteria such as *Prevotella* spp. and *Clostridium difficile* (Lawther et al., 2022).

Trimethoprim (TMP) resistance is caused by dihydrofolate reductases which transform dihydrofolate to tetrahydrofolate, which is necessary for the generation of nucleic acid precursors. *dfrE* is already present in several organisms from diverse geographical locations. *dfrE* has been widely distributed in Asia, where it is found in at least three staphylococcal species (*S. sciuri, S. aureus*, and *S. arlettae*) of human and animal origin (including clinical samples), as well as the livestock-associated *M. caseolyticus* strain JCSC5402 (65) from various nations (Gómez-Sanz et al., 2021). *Sul1* genes provide resistance to sulfonamides. The average number of copies of the *sul1* gene appeared to be lower in soils modified with manure than in the unaltered sample (Han et al., 2021). The application of aminoglycosides against bacteria that are resistant to beta-lactams and fluoroquinolones indicates the prevalence of MDR bacteria in healthcare facilities. Proteobacteria harboured resistance genes for aminoglycosides [*APH (6)-Id*], beta-lactamases, macrolides, and sulfonamides (Talat et al., 2023). Actinobacteria and Proteobacteria make up the majority of antibiotic-resistant bacteria (Qiao et al., 2021). The antibiotic resistance genes, known to be resistant against aminoglycoside [*aadA5, APH(6)-Id,* and *APH (6)-Ia*], tetracycline [*tet(G), tet(C), tet(M), tetW, tetQ, and tet(39)*] and sulphonamide (*sul1* and *sul2*) were also detected in the sediment samples of the river Yamuna in India (Das et al., 2020). Aminoglycoside resistance genes such as *aac(6′)-Ib, aadS, acrD,* and *ANT(2′′)-Ia* were most commonly reported in sediments of Rasulabad Ghat, with aadS also prevalent in Bagwan and Triveni Sangam in river Ganga of India (Rout et al., 2023).

### 3.11 Identification and composition of heavy metal resistance genes

A total of 350 heavy metal resistance genes related to 29 metals were identified by BacMet2 database annotation across all the samples. The resistance genes for Cu, As, Cr, Zn, Ag, and Fe were abundant. Multiple metal resistance genes were also an important part of the MRGs, accounting for 33 to 53% of the total MRGs. Most of the multi-metal resistance genes were linked to Cd, Zn, Co, Ni, Cr, Te, Se, V, Mo, Mn, Fe, Bi, Zn, Pb, and Mg. Supplementary Figure 16 shows the pie charts depicting the percentage of heavy metal-resistance genes (HMRGs) detected across the samples. These HMRGs were resistant to 29 different metals, among whichcopper (Cu), arsenic (As), iron (Fe), nickel (Ni), zinc (Zn), mercury (Hg), and chromium (Cr) resistance genes were abundant (Supplementary Table 4). Many of these genes are resistant to multiple heavy metals, andare classified asmulti-metal resistance genes. In all the samples, the highest percentage of the HMRGs belonged to multi-metal genes, with AGS (53%) having the highest percentage, followed by THS (51%), NPS (49%), DYS (48%), TMS (47%), CSS (45%), PIS (39%), and TPS (33%). The Cu resistance genes included *ricR, copF, cusA, ybdE, copA, ctpV, copB,* and *actP;* the arsenic resistance genes *arsM, pstB,* and *arsT;* the Fe resistance genes *furA* and *can*; and the Ag resistance genes *cusA/ybdE* and *silP;* which were the top 15 MRGs in terms of abundance. The *ruvB* gene is commonly involved in resistance to chromium (Cr), tellurium (Te), and selenium (Se) (Figure 8B). The HMR gene *arsM* had significant negative correlations with *Mycobacterium canettii* (*r* = −0.77, *p*-value = 0.025)*, Rhodopseudomonas palustris* (*r* = −0.78, *p*-value = 0.021)*, Bradyrhizobium* sp. (*r* = −0.77, *p*-value = 0.024)*, Rhizobium* sp. (*r* = −0.77, *p*-value = 0.025), *Cupriavidus taiwanensis* (*r* = −0.77, *p*-value = 0.026)*, Sorangium cellulosum* (*r* = −0.78, *p*-value = 0.02), and *Ramlibacter tataouinensis* (*r* = −0.78, *p*-value = 0.02). The AMR gene *mex_1* had positive correlations with HMR genes such as *aioE* (*r* = 0.98, *p*-value = 0.00002) and *arsT* (*r* = 0.98, *p*-value = 0.00001). Meanwhile, *emrA_1* and *iri_1* were negatively correlated with *ruvB* (*r* = −0.8, *p*-value = 0.018) and *chrA* (*r* = −0.92, *p*-value = 0.001), respectively (Supplementary Figure 15).

Soil microorganisms have the natural ability to withstand and resist heavy metals. Resistance is the capacity to develop successfully in an environment of continual prohibitive quantities of a chemical, whereas tolerance is the ability to remain latent and be sustained in surroundings containing the compound without considerable growth (Muñoz-García et al., 2022). Tolerance and resistance are primarily based on the permanent formation of complexes with biosurfactants and active metal drainage via efflux transporters (Herrera-Calderon et al., 2024). The *CopA* genes, which encode the multi-copper oxidase, convert Cu (I) to the less hazardous chemical form Cu (II). The existence of the *copA* gene in bacterial populations indicates that the cop system associated with Cu-resistance may be common in soil, most likely because of horizontal gene transfer between soil bacteria. The copA gene was found solely in metagenomic DNA from Cu-polluted soils, indicating that the *copA* gene is widespread in polluted habitats. It is extensively prevalent in Cu-resistant bacterial strains and may serve as a useful marker for studying Cu-resistance in bacteria (Altimira et al., 2012). CopA consists of eight transmembrane domains and two cytoplasmic heavy metal interaction motifs with cooper-coordinating (CXXC) domains and has been demonstrated to efflux copper ions from the cytoplasm (Sitthisak et al., 2007). The *copA* gene produces copper-transporting P-type ATPases that restrict Cu from infiltrating the cytosol and aid in intracellular detoxification (Li et al., 2015a). *CopA* needs ATP to eliminate heavy metals from the outer layer of the cell; however; *CusA* is an energy-free process that essentially requires an antiporter component (Besaury et al., 2013). *M. tuberculosis* has been found to require *CtpV* to maintain its resistance to copper toxicity (Ward et al., 2010). *M. tuberculosis* possesses multiple Cu-responsive pathways, including the RicR regulon, which is specific to infectious mycobacteria. Although host-supplied Cu may prevent the proliferation of bacteria, *M. tuberculosis* possesses a distinct defense mechanism, RicRregulon, which prevents Cu cytotoxicity (Shi et al., 2014). *CtpC* enhances *M. tuberculosis* tolerance to zinc toxicity andincreases the intracellular viability in macrophages (Botella et al., 2011).

### 3.12 Metabolic genes diversity

The top 20 metabolic genes identified from soils and are presented in Figure 8C. Sulfate reducers, nitrite reducers, ammonia oxidizers, dehalogenations, sulfide oxidizers, chitin degradation, nitrogen fixation, xylan degraders, atrazine metabolism, aromatic hydrocarbon degraders, lignin degraders, chlorophenol degrading, naphthalene degrading, and carbon fixation were identified as major metabolic genes in all the samples.

### 3.13 Functional characterization of the soil bacterial population

**Figure 9:**
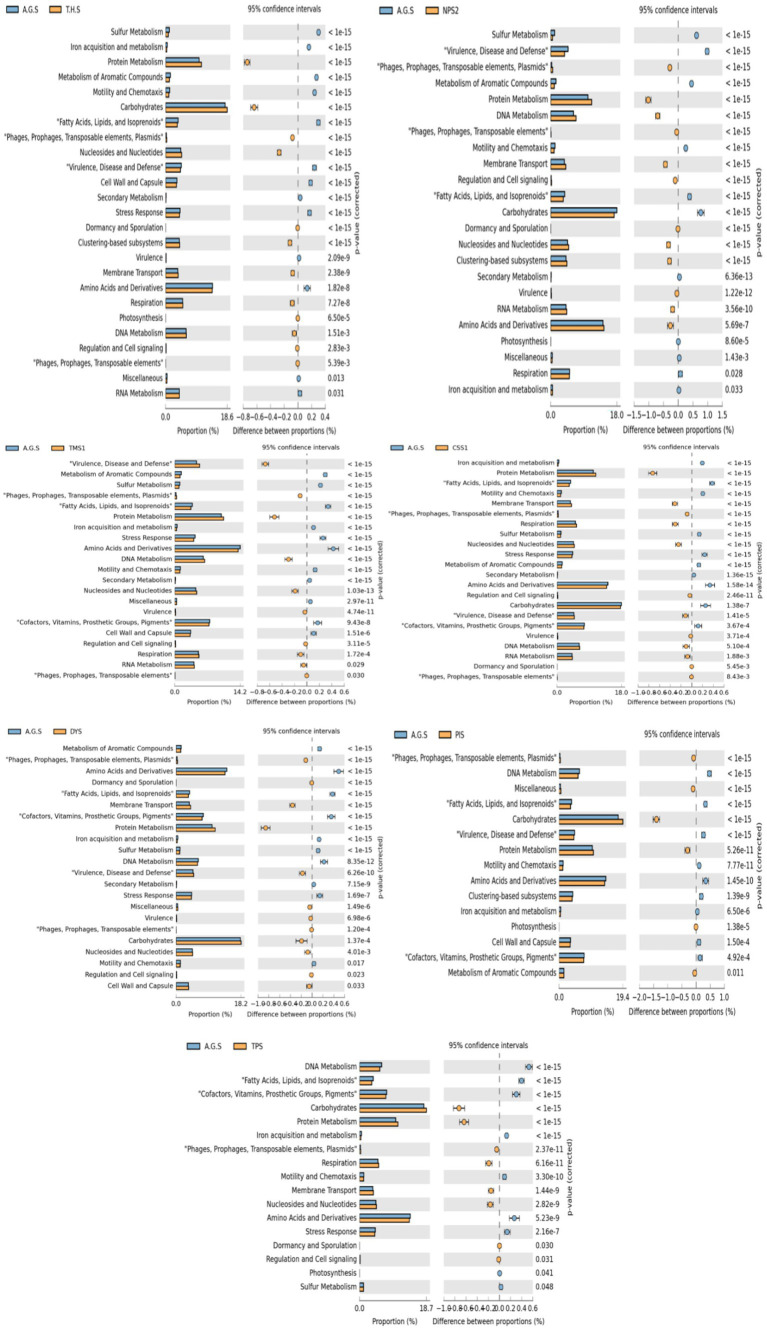
Difference of functional genes categorized into different subsystem between different soil samples. The comparative analysis of metabolic pathways was conducted using Fisher’s exact test by STAMP software. *p* ≤ 0.05 filter was used.

The Figure 9 depict the comparative analysis of the functional profiles of microbial communities across the HHMC and LHMC samples with the AGS, analyzed using PICRUSt, which is based on the KEGG pathways. Even though the AGS sample was detected to have higher concentrations of heavy metals, the AGS sample was a garden sample with the least livestock and human exposure (least disturbance) compared with all other samples. Hence, we compared all the samples with AGS here. Pb, Cr, Fe, Cu, Cd, 1-dodecanol, ethyl acetate, undecane, 3, 8-dimethyl, docosane, tricontane, henicosane, and tetrachloroethylene were detected to be higher in the AGS sample. Moreover, the AGS sample had the lowest soil pH among the soil samples collected in the present study. The functional predictions of the microbial community revealed that protein metabolism, carbohydrates, amino acids and derivatives, and DNA metabolism were the major functional categories across all three HHMC samples (Figure 9). Compared with the AGS sample, the THS and NPS samples presented agreater proportion of genes for protein metabolism, DNA metabolism, ‘dormancy and sporulation,’ and ‘phages and transposable elements.’ Meanwhile, the AGS samplespresented a greater proportion of ‘virulence disease and defence,’ sulphur metabolism, ‘Iron acquisition and metabolism’, and ‘motility and chemotaxis’ genes. Comparedwith the AGS samples, the THS samplespresented greater ‘stress response’ and ‘virulence’ genes. Protein metabolism, amino acids and derivatives, and DNA metabolism were the major functional gene categories across all the LHMC samples (Figure 9). Comparied with AGS samples, all five LHMC samples presented greater proportions of ‘phages, prophages, transposable elements, plasmids’ and ‘iron acquisition and metabolism’ genes. Moreover, ‘virulence’ and ‘virulence, disease and defense’ were higher in TMS, CSS and DYS. Moreover, compared with those of the LHMC samples, ‘motility and chemotaxis’ and ‘stress response’ were higher in the AGS samples. A higher proportion of ‘phages, prophages, and transposable elements in LHMC samples revealed a relationship between hydrocarbon pollution and the emergence of antibiotic resistance in the microbiome, which is not limited to therapeutic drug use. Increased sulphur metabolism levels in AGS may explain the larger predicted prevalence of genes associated with the manufacture of the amino acids cysteine and methionine (Chung et al., 2019). Pollution affects the functional variety and number of soil microbiomes, as stated earlier (Das et al., 2021). The KEGG orthology analysis found that the samples had a wide range of metabolic categories; including carbohydrate metabolism, membrane transport, and amino acid metabolism in the Ganga silt (Rout et al., 2024a). These locations contain abundant material which encourages diverse microbes (Rout et al., 2024b). Antibiotic-resistant bacteria could become more prevalent in polluted soil because of coselection forces from pollutants such as metals and hydrocarbons (Cunningham et al., 2020).

### 3.14 Virulence genes diversity

Virulence factors facilitate the spread of AMR genes across environments by forming biofilms and increasing disease transmission (Talat et al., 2023). The major virulence genes detected in the AGS samples were*espR* and *regX3*. However, the *sugB, whiB3, mmPL3, chpA, pks, sigA/rpoV, fliA, fadD26, clpC* and *algC*genes were predominant in the THS sample. *sigA/rpoV, prrA, eccC5, groEL2, narG, sugB, aftD, eccA3* and *eccB5* were the major genes detected in NPS. Moreover, the CSS sample presentedincreased abundances of the *entB, ybtE, acpXL, cheY, entF* and *fimF* genes. *Bap, regX3*, and *fbpC*were predominant in DYS. The major genes detected in PIS were *sigA/rpoV* and *Bap*. Moreover, the *irp1, entC, espR, fepA,* and *fimB* genes were predominant in TMS. Moreover, the *cap8E, cap8D* and *sigA/rpoV* genes are abundant in the TPS (Supplementary Figure 17). Following a thorough screening of the protein–protein interaction (PPI) network, 14 genes were identified as potentially important, and their respective string network diagrams were generated. The end result is a PPI relationship network with fourteen nodes and 65 edges. The *p*-value for PPI enrichment was calculated to be 0.00012. The Mixed, incl. Adenovirus iva2 protein, eccd-transmembrane domain, cellular response to decreased oxygen levels, and phosphorelay response regulator activity were significantly enriched (Supplementary Figure 18). In this study, we identified multiple virulence factors predominantly associated with the pathogenic genomes of *Mycobacterium*, *Klebsiella*, *Pseudomonas*, *Acinetobacter*, and *Staphylococcus*. These virulence determinants play a crucial role in the pathogenicity (Supplementary Table 5).

*Mycobacterium tuberculosis* needs the ESX-1 secretion system to be fully virulent. EspR regulates ESX-1 by immediate association and transcriptional stimulation of the espACD operon (Blasco et al., 2011). Reduced potassium ion levels cause *M. tuberculosis* to become latent. *RegX3* plays a role in *M. tuberculosis* viability during potassium deficiency (Bagchi et al., 2024). The trehalose-recycling ABC transporter LpqY-SugA-SugB-SugC is required for the pathogenicity of *M. tuberculosis* (Kalscheuer et al., 2010). *WhiB3* regulates mycobacterial pathogenicity by inhibiting phagosomal development and controlling the cell cycle. *WhiB3* prevents acidic pH within cells by maintaining the mycothiol redox pathway. *WhiB3* has been linked to the mechanism of lipid metabolism under dormancy, which regulates lipid breakdown and the manufacture of reserve lipids such as triacylglycerols (TAG) that produce inclusion complexes. *WhiB3* promotes the synthesis of lipids which trigger inflammatory processes in pathogenic strains (Barrientos et al., 2022). The pks gene encodes a cyclomodulin called colibactin, which causes DNA damage, disturbs the eukaryotic cell cycle, and leads to the onset and development of colorectal cancer (Sadeghi et al., 2024). AlgC is required for the biosynthesis of UDP-glucose, a substrate for the production of lipopolysaccharide (LPS) in *H. pylori* (Feng et al., 2023). PrrA acts as a transcription factor, regulating the intensity of the response of Mtb to pH, Cl-, NO, and hypoxia (Giacalone et al., 2022). The *M. tuberculosis* protein GroEL2, a chaperone-like immunomodulatory protein, regulates proinflammatory responses from macrophages and dendritic cells (DCs), induces maturation of DCs, and facilitates antigen presentation to T cells (Georgieva et al., 2018). MmpL3 is a member of the RND protein superfamily, which includes inner membrane transporters. The over expression of MmpL3 is required for the survival of *M. tuberculosis* (Bolla, 2020). Arabinofuranosyltransferase D (AftD) is a key enzyme in the glycolipid assembly process. AftD is a crucial enzyme for cell wall biosynthesis routes in mycobacteria and other mycolic acid-producing bacteria in the order*Corynebacteriales* (Tan et al., 2020). *Cap8D* encodes a dehydratase that is required for the production of the capsule precursor implicated in adhesion (Sinha et al., 2021). The *entC* is a key virulence gene associated with iron absorption in *K. pneumoniae* (Zhang et al., 2022). *M. smegmatis* requires the phosphorus recognizing gene *RegX3* for survival on propionate and persistance in macrophages. *RegX3* aids *M. smegmatis* growth and persistence by altering the shape of thestrain in macrophages (Pei et al., 2021). Biofilm-associated protein (Bap) and its homologues have been demonstrated to be involved in multicellular assembly and biofilm development in a variety of species, including staphylococci and enterococci. Calcium ions in the environment regulate Bap-mediated biofilm formation by preventing the amyloid assembly of Bap-derived peptides and resulting in intercellular adhesion (Schiffer et al., 2021). The *entB* gene increases the virulence of hypervirulent *K. pneumoniae* (hvKP) by producing abundant enterobactin, which aids in iron intake and biofilm development (Han et al., 2022). The EccB5 protein in the ESX-5 system could be a key membrane protein associated with the transmission mechanisms of the type VII secretion system, which is required for the proliferation and pathogenicity of pathogenic bacteria (Kurniawati et al., 2020). *AlgC* is required for the biosynthesis of UDP-glucose, which is the basis for the production of lipopolysaccharide (LPS) in *H. pylori* (Feng et al., 2023). The deactivation of clpC in *S. aureus* increases permanent intracellular persistence in phagocytes (Gunaratnam et al., 2019).

## 4 Conclusion

This study highlights the substantial impacts of heavy metal and hydrocarbon contamination on microbial diversity, resistance mechanisms, and soil functionality. The high Heavy Metal Concentration (HHMC) zones (AGS, THS, and NPS) exhibited diminished pH levels and lowered dehydrogenase activity, reflecting microbial alterations that suggest a detrimental impact on soil health and metabolic activities. The HHMC regions exhibited elevated levels of antibiotic resistance genes (ARGs), heavy metal resistance genes (HMRs), and virulence genes (VGs), indicating that the microbial community is affected by environmental stresses. Despite a reduction in overall microbial diversity, the prevalence of Actinobacteria in certain soils indicates that these organisms have adapted ecologically to thrive under adverse conditions.

The Low Heavy Metal Concentration (LHMC) zones (CSS, TMS, TPS, DYS, and PIS) had greater microbial diversity than did the High Heavy Metal Concentration (HHMC) regions. CSS and TMS are distinguished by the lowest concentrations of heavy metals, hydrocarbon contaminants, and HMR genes among the LHMC regions. The lower dehydrogenase activity observed in the CSS and TMS locations than in the other LHMC areas signifies a compromised microbial response to ecological stresses. Concurrently, these samples exhibited substantially increased proportions of AMR and VGs, which may be attributed to the presence of augmented pathogenic microorganisms such as *Mycobacterium cannetti, Stenotrophomonas maltophilia, Cryptococcus neoformans, Leishmania major, Leishmania mexicana, Leishmania brazilensis, Babesia bovis, Babesia bigemina, Neospora caninum,* and *Besnoitia besnoiti*. In contrast, TPS, DYS, and PIS, which possessed a diminished quantity of AMR and VR genes, demonstrated a lower ability for microbial adaptation to environmental conditions.

Heavy metal and hydrocarbon contamination profoundly influences microbial diversity, metabolic adaptations, and the prevalence of resistance genes in samples. The intraphylum relationships within the microbial communities and the positive correlations of heavy metals and alkanes with microbial taxa such as *Nocardiopsis alba, Streptomyces, Pseudomonas, Xanthomonas, Rhodococcus, Kocuria rosea, Paracoccus, Nocardioides* sp. CF8, *Haloferax, Aspergillus* and *Fusarium* reveal the role of microbial adaptability in the bioremediation of environmental pollutants. HHMC locations exhibit minimal microbial diversity but more robust survival mechanisms, whereas LHMC areas maintain microbial richness alongside increased antibiotic resistance, highlighting the ecological effects of environmental contaminants on microbial composition and soil activity. The results demonstrate that there is a distinct correlation between environmental contaminants and the emergence of microbial adaptation with respect to the soil habitat. This highlights the resilience of microorganisms and the possible bioremediation activities that they can perform in contaminated environments. A comprehensive understanding of these interactions is essential for the development of efficient bioremediation solutions and the evaluation of long-term ecological alterations in compromised ecosystems.

## Data Availability

All data confirming the study’s conclusions are included in the main article text and supplementary file. Sequence data supporting the outcomes of this work have been deposited in the NCBI-SRA database under the accession number PRJNA1276251.
