# Supplementary material for: Comprehensive whole metagenomics analysis uncovers microbial community and resistome variability across anthropogenically contaminated soils in urban and suburban areas of Tamil Nadu, India
Source: Front Microbiol. 2025 Oct 22;16:1649872. doi: 10.3389/fmicb.2025.1649872 (PMC12586116; doi:10.3389/fmicb.2025.1649872)
Supplement: Supplementary file 1 [file Data_Sheet_1.docx]

**Supplementary Table 1: Soil Physio-chemical properties and heavy metal characteristics across all the soil samples**

| Samples | AGS | NPS | CSS | TMS | THS | DYS | PIS | TPS |
| --- | --- | --- | --- | --- | --- | --- | --- | --- |
| sample type | Garden (Control) | Poultry | Cattle | Market | Hospital | Dump yard | Paper industry | Thermal power station |
| Soil pH | 6.9 | 8.17 | 8.82 | 9.63 | 7.41 | 8.86 | 8.15 | 8.87 |
| Total organic carbon  (%) | 0.97 | 6.71 | 0.114 | 0.123 | 1.8 | 0.79 | 0.44 | 0.131 |
| Nitrogen (kg/ha) | 244 | 269 | 194 | 162 | 381 | 478.93 | 201 | 324.5 |
| Phosphorus (mg/kg) | 73.6 | 1.25 | 219.4 | 16.4 | 48.1 | 41.8 | 40.3 | 18.69 |
| Potassium (kg/ha) | 70.5 | 1299 | 148 | 126 | 189 | 230 | 112.7 | 111 |
| Sodium (kg/ha) | 309 | 3963 | 103.6 | 112 | 322 | 471 | 71.6 | 85.86 |
| Calcium (mg/kg) | 872 | 2548 | 465 | 111.6 | 617 | 179 | 375 | 219.3 |
| Magnesium (mg/kg) | 9.14 | 833 | 677.9 | 700.6 | 101 | 242 | 96.2 | 483.8 |
| Iron  (ppm) | 169 | 157 | 82.7 | 60.3 | 98.6 | 112.6 | 63.12 | 87.81 |
| Zinc (ppm) | 0.27 | 24.6 | 16.7 | 5.32 | 0.19 | 3.98 | 2.74 | 1.41 |
| Copper (ppm) | 4.21 | 4.872 | 0.347 | 0.845 | 3.98 | 1.421 | 1.037 | 0.584 |
| Manganese (mg/kg) | 19.6 | 314.92 | 885.62 | 1059.7 | 17.5 | 587.32 | 285.23 | 1889.32 |
| Aluminium(mg/kg) | 1.96 | 106.39 | 296.38 | 308.3 | 3.41 | 213.88 | 136.88 | 628.15 |
| Lead (ppm) | 4.08 | 1.768 | 0.007 | 0.026 | 3.91 | 0.59 | 0.22 | 1.83 |
| Nickel (ppm) | 0.21 | 0.166 | 0.01 | 0.012 | 0.17 | 0.79 | 0.27 | 0.08 |
| Chromium(ppm) | 2.41 | 1.034 | 0.042 | 0.0088 | 1.98 | 0.091 | 0.053 | 0.088 |
| Cadmium(ppm) | 0.27 | 0.316 | 0.017 | 0.047 | 0.16 | 0.079 | 0.061 | 0.014 |

**Supplementary Table 2: List of major metabolites/pollutants identified across the samples by GC–MS analysis**

| **Compound Name** | **Average Retention**  **Time (min)** | **Component Area** | **Chemical classification on the basis of IUPAC name by ClassyFire*** |
| --- | --- | --- | --- |
| Decane, 1-iodo | 24.65±2.13 | 276262.2 (THS)  233843.5 (CSS)  207328.6 (TMS)  321746.8 (NPS) | Organoiodides |
| Squalene | 33.55±0.01 | 579209.1 (THS)  2865420.4 (TMS)  1837875.0 (NPS)  2021009.8 (PIS)  2889677.5 (DYS) | Triterpenoids |
| Phthalic acid, 6-ethyl-3-octyl butyl ester | 25.17±0.58 | 1006553.9 (TMS)  123181.9 (PIS)  421271.3 (DYS) | Benzoic acid esters |
| Methyl stearate | 27.36±0.01 | 389105.1 (THS)  340848.5 (CSS)  385846.4 (TMS)  1653528.4 (NPS)  662057.5 (PIS)  795396.9 (DYS)  409438.0 (TPS) | Fatty acid methyl esters |
| 1-Hexadecanol | 24.09±2.92 | 849975.2 (THS)  714973.5 (CSS)  839415.1 (TMS)  2038047.0 (NPS)  947292.6 (PIS)  794819.5 (DYS)  589735.4 (TPS) | Long-chain fatty alcohols |
| Hexadecanoic acid methyl ester | 24.9±0.37 | 956628.4 (AGS)  2106315.3 (THS)  32471.4 (CSS)  3415097.4 (TMS)  3182228.3 (NPS)  1360458.3 (PIS)  1842273.8 (DYS)  2108710.3 (TPS) | Fatty acid methyl esters |
| 1,3-Benzenedicarboxylic acid, bis(2-ethylhexyl) | 32.93±0.003 | 638541.7 (THS)  1424033.3 (TMS) | m-Phthalate esters |
| Acetic acid | 3.23±0.13 | 4818174.3 (THS)  13279334.6 (TMS)  232173.1 (NPS) | Carboxylic acids |
| Didecan-2-yl phthalate | 33.63±0.001 | 878913.0 (THS)  1131238.5 (TMS)  491292.9 (DYS) | Benzoic acid esters |
| dl-.alpha.-Tocopherol | 36.81±0.01 | 751748.6 (THS)  468309.9 (CSS)  1462594.5 (TMS)  1110970.1 (PIS) | Tocopherols |
| Phthalic acid, di(2-propyl pentyl) ester | 31.38±0.009 | 10436707.4 (THS)  40078693.5 (TMS)  1019470.3 (NPS)  12104314.6 (DYS) | Benzoic acid esters |
| Phthalic acid, cyclobutyl ethyl ester | 19.91±0.27 | 5640405.5 (TMS)  11155909.7 (DYS) | Benzoic acid esters |
| 2-Pentadecanone, 6,10,14-trimethyl | 24.2±0.02 | 376384.8 (THS)  447142.1 (CSS)  705178.6 (TMS)  888992.8 (PIS)  1162898.0 (DYS) | Sesquiterpenoids |
| Tetracosane | 29.5±1.06 | 1164198.8 (THS)  727500.1 (NPS)  500603.0 (PIS)  1076726.1 (DYS) | Alkanes |
| Pentacosane | 30.46±3.1 | 1248550.9 (THS)  895088.0 (TMS)  1455156.9 (NPS)  1191674.4 (PIS)  1992186.9 (DYS) | Alkanes |
| Triacontane | 33.56±2.39 | 619762.1(AGS)  712659.8 (THS)  4457464.8(PIS)  1271879.1(DYS) | Alkanes |
| Docosane | 28.1±0.005 | 354007.5 (AGS)  1469962.0 (THS)  485355.2 (TMS) | Alkanes |
| Hexadecane, 1-iodo- | 32.83±4.76 | 351423.3 (NPS)  333543.7 (TPS) | Organoiodides |
| Tetrachloroethylene | 3.48±0.16 | 6253454.0 (AGS)  4799962.5 (THS)  46622313.2 (NPS)  3821929.4 (PIS)  2681066.9 (DYS)  4280105.0 (TPS) | Vinyl chlorides |
| Eicosane | 27.35±2.14 | 357768.6 (NPS)  388384.6 (TPS) | Alkanes |
| Heptadecane | 24.15±3.3 | 1037429.7 (THS)  413554.4 (TMS)  469034.7 (NPS)  276086.4 (PIS) | Alkanes |
| Ammonium acetate | 3.36±0.22 | 7662441.5 (CSS)  27132195.7 (NPS) | Acetate |
| Diethyl Phthalate | 20.99±0.31 | 1582106.3 (TMS)  68283840.7 (NPS) | Benzoic acid esters |
| 1-Dodecanol | 21.47±5.07 | 144184.9 (AGS)  268185.8 (TMS)  62433.4 (NPS)  235700.6 (PIS)  299483.3 (TPS) | Fatty alcohols |
| Ethyl Acetate | 3.12±0.005 | 3580015.9 (AGS)  3744698.7 (PIS)  3657086.2 (DYS)  4696070.3 (TPS) | Carboxylic acid esters |
| 1-Octadecene | 23.6±0.008 | 1869715.0 (NPS)  778130.9 (PIS)  1245844.4 (DYS) | Unsaturated aliphatic hydrocarbons |
| 2,6,10,14-Tetramethylpentadecane | 24.23±0.64 | 70908.9 (CSS)  863951.5 (NPS) | Acyclic diterpenoids |
| Behenic alcohol | 25.78±0.01 | 1343239.1 (NPS)  1613155.7 (DYS) | Fatty alcohols |
| Octadecane | 27.2±4 | 398671.0 (NPS)  1406313.7 (DYS) | Alkanes |
| Heneicosane | 26.13±2.75 | 731919.2 (AGS)  776818.5 (TMS)  348613.7 (NPS)  395361.4 (PIS)  557927.1 (DYS) | Alkanes |

**Supplementary Table 3: List of major metabolites identified across the soil samples by GC–MS analysis and toxicity and adverse effects analysis by ProTox-II web server**

| **Metabolites** | **LD**  **50 (mg/kg)** | **Toxicity Class** | **Hepatotoxicity** | **Carcinogenicity** | **Immunotoxicity** | **Mutagenicity** | **Cytotoxicity** | **Sample** |
| --- | --- | --- | --- | --- | --- | --- | --- | --- |
| **Tetrachloroethylene** | 2400 | 5 | Inactive (0.92) | **Active**  **(0.97)** | Inactive (0.99) | Inactive  (0.91) | Inactive  (0.62) | AGS, THS,  NPS,  PIS,  DYS,  TPS |
| 2-Pentadecanone, 6,10,14-trimethyl | 5000 | 5 | Inactive  (0.72) | Inactive  (0.75) | Inactive  (0.99) | Inactive  (0.86) | Inactive  (0.77) | THS,  TMS,  CSS,  PIS,  DYS,  TPS |
| Heneicosane | 750 | 3 | Inactive  (0.74) | Inactive  (0.58) | Inactive  (0.98) | Inactive  (1.0) | Inactive  (0.78) | AGS,  TMS,  NPS,  PIS,  DYS,  TPS |
| Squalene | 5000 | 5 | Inactive  (0.79) | Inactive  (0.76) | Inactive  (0.99) | Inactive  (0.98) | Inactive  (0.81) | THS,  TMS,  NPS |
| Pentacosane | 750 | 3 | Inactive  (0.74) | Inactive  (0.58) | Inactive  (0.98) | Inactive  (1.0) | Inactive  (0.78) | THS,  TMS,  NPS,  DYS,  TPS |
| Neophytadiene | 5050 | 6 | Inactive  (0.79) | Inactive  (0.73) | Inactive  (0.99) | Inactive  (0.98) | Inactive  (0.81) | THS,  CSS,  PIS,  DYS |
| Undecane, 3,8-dimethyl | 750 | 3 | Inactive  (0.75) | Inactive  (0.62) | Inactive  (0.99) | Inactive  (0.97) | Inactive  (0.79) | AGS,  CSS,  TPS |
| Heptadecane | 750 | 3 | Inactive  (0.74) | Inactive  (0.58) | Inactive  (0.98) | Inactive  (1.0) | Inactive  (0.78) | THS,  NPS,  PIS |
| Decane, 1-iodo | 5000 | 5 | Inactive  (0.96) | Inactive  (0.74) | Inactive  (0.98) | Inactive  (0.55) | Inactive  (0.67) | TMS,  NPS |
| Diethyl Phthalate | 6172 | 6 | Inactive  (0.77) | Inactive  (0.65) | Inactive  (0.99) | Inactive  (0.87) | Inactive  (0.92) | TMS,  NPS |
| Phthalic acid, cyclobutyl ethyl ester | 3474 | 5 | Inactive  (0.65) | Inactive  (0.68) | Inactive  (0.98) | Inactive  (0.80) | Inactive  (0.82) | TMS,  DYS |
| **Mahanimbine** | 4000 | 5 | Inactive  (0.63) | Inactive  (0.68) | **Active**  **(0.97)** | Inactive  (0.68) | Inactive  (0.85) | TMS,  DYS |
| Cyclohexane, 1,3,5-triphenyl- | 6430 | 6 | Inactive  (0.80) | Inactive  (0.70) | Inactive  (0.99) | Inactive  (0.94) | Inactive  (0.91) | TMS,  DYS |
| **Hexadecane, 1-iodo-** | 1360 | 4 | Inactive  (0.84) | **Active**  **(0.50)** | Inactive  (0.97) | Inactive  (0.93) | Inactive  (0.79) | NPS,  TPS |
| Eicosane | 750 | 3 | Inactive  (0.74) | Inactive  (0.58) | Inactive  (0.98) | Inactive  (1.0) | Inactive  (0.78) | THS,  NPS,  TPS |
| Docosane | 750 | 3 | Inactive  (0.74) | Inactive  (0.58) | Inactive  (0.98) | Inactive  (1.0) | Inactive  (0.78) | AGS,  THS |
| 2,6,10,14-Tetramethylpentadecane | 750 | 3 | Inactive  (0.80) | Inactive  (0.74) | Inactive  (0.99) | Inactive  (0.90) | Inactive  (0.81) | NPS,  DYS |
| Octadecane | 750 | 3 | Inactive  (0.74) | Inactive  (0.58) | Inactive  (0.98) | Inactive  (1.0) | Inactive  (0.78) | NPS,  DYS |
| 1-Hexadecanol | 1000 | 4 | Inactive  (0.90) | Inactive  (0.5) | Inactive  (0.97) | Inactive  (1.0) | Inactive  (0.83) | THS,  TMS,  CSS,  NPS,  PIS,  DYS,  TPS |
| Hexadecanoic acid methyl ester | 5000 | 5 | Inactive  (0.58) | Inactive  (0.55) | Inactive  (0.99) | Inactive  (0.98) | Inactive  (0.73) | AGS,  THS,  TMS,  CSS,  NPS,  PIS,  DYS,  TPS |
| Methyl stearate | 5000 | 5 | Inactive  (0.58) | Inactive  (0.55) | Inactive  (0.99) | Inactive  (0.98) | Inactive  (0.73) | THS,  TMS,  CSS,  NPS,  PIS,  DYS,  TPS |
| Dodecyl acrylate | 5000 | 5 | Inactive  (0.75) | Active  (0.61) | Inactive  (0.84) | Inactive  (1.0) | Inactive  (0.75) | THS,  TMS,  CSS,  DYS,  TPS |
| **Hexadecanoic acid, ethyl ester** | 5000 | 5 | Inactive  (0.76) | **Active**  **(0.56)** | Inactive  (0.99) | Inactive  (0.99) | Inactive  (0.78) | THS,  TMS,  CSS,  PIS,  DYS,  TPS |
| **Phthalic acid, di(2-propylpentyl) ester** | 1340 | 4 | Inactive  (0.80) | **Active**  **(0.79)** | Inactive  (0.99) | Inactive  (0.95) | Inactive  (0.87) | AGS,  THS,  TMS,  CSS,  NPS,  DYS |
| **Ethyl Acetate** | 4100 | 5 | Inactive  (0.84) | **Active**  **(0.62)** | Inactive  (0.99) | Inactive  (0.93) | Inactive  (0.82) | AGS,  PIS,  DYS,  TPS |
| **gamma.-Sitosterol** | 890 | 4 | Inactive  (0.87) | Inactive  (0.60) | **Active**  **(0.99)** | Inactive  (0.98) | Inactive  (0.94) | TMS,  CSS,  TPS |
| dl-.alpha.-Tocopherol | 5000 | 5 | Inactive  (0.93) | Inactive  (0.79) | Inactive  (0.95) | Inactive  (0.95) | Inactive  (0.89) | THS,  TMS,  CSS |
| Tetracosane | 750 | 3 | Inactive  (0.74) | Inactive  (0.58) | Inactive  (0.98) | Inactive  (1.0) | Inactive  (0.78) | THS,  NPS,  DYS |
| **Phthalic acid, di(2-propyl pentyl) ester** | 1340 | 4 | Inactive  (0.82) | **Active**  **(0.86)** | Inactive  (0.97) | Inactive  (0.99) | Inactive  (0.87) | THS,  TMS,  NPS,  DYS |
| **(E)-9-Octadecenoic acid ethyl ester** | 5000 | 5 | Inactive  (0.76) | **Active**  **(0.53)** | Inactive  (0.97) | Inactive  (0.98) | Inactive  (0.76) | TMS,  DYS,  TPS |
| Hexadecane, 2-methyl- | 750 | 3 | Inactive  (0.77) | Inactive  (0.72) | Inactive  (0.98) | Inactive  (0.94) | Inactive  (0.77) | THS,  TMS,  NPS |
| Undecane, 3,8-dimethyl- | 750 | 3 | Inactive  (0.75) | Inactive  (0.62) | Inactive  (0.99) | Inactive  (0.97) | Inactive  (0.79) | AGS,  CSS,  TPS |
| Triacontane | 750 | 3 | Inactive  (0.74) | Inactive  (0.58) | Inactive  (0.98) | Inactive  (1.0) | Inactive  (0.78) | AGS,  THS,  DYS |
| 1-Octadecene | 5050 | 6 | Inactive  (0.75) | Inactive  (0.59) | Inactive  (0.95) | Inactive  (1.0) | Inactive  (0.77) | NPS,  PIS,  DYS |
| Didecan-2-yl phthalate | 1340 | 4 | Inactive  (0.65) | Active  (0.59) | Inactive  (0.65) | Inactive  (0.97) | Inactive  (0.76) | THS,  TMS |
| Acetic acid | 333 | 1 | Inactive  (0.80) | Inactive  (0.72) | Inactive  (0.99) | Inactive  (0.98) | Inactive  (0.77) | THS,  TMS |
| **1,3-Benzenedicarboxylic acid, bis(2-ethylhexyl)** | 12300 | 6 | Inactive  (0.81) | **Active**  **(0.83)** | Inactive  (0.98) | Inactive  (1.0) | Inactive  (0.90) | THS,  TMS |
| **Pentadecanoic acid, ethyl ester** | 5000 | 5 | Inactive  (0.76) | **Active**  **(0.56)** | Inactive  (0.99) | Inactive  (0.99) | Inactive  (0.78) | TMS,  PIS |
| **Phthalic acid, 6-ethyl-3-octyl butyl ester** | 1340 | 4 | Inactive  (0.74) | **Active**  **(0.68)** | Inactive  (0.87) | Inactive  (0.94) | Inactive  (0.84) | TMS,  PIS |
| Pentadecanal- | 5000 | 5 | Inactive  (0.71) | Inactive  (0.59) | Inactive  (0.95) | Inactive  (0.96) | Inactive  (0.73) | TMS,  DYS |
| Cyclic octaatomic sulfur | 190 | 3 | Inactive  (0.79) | Inactive  (0.53) | Inactive  (0.99) | Inactive  (0.68) | Inactive  (0.87) | TMS,  CSS |
| **gamma.-Sitostenone** | 2300 | 5 | Inactive  (0.80) | Inactive  (0.59) | **Active**  **(0.99)** | Inactive  (0.97) | Inactive  (0.74) | CSS,  TPS |
| 1-Dodecanol | 1000 | 4 | Inactive  (0.90) | Inactive  (0.5) | Inactive  (0.97) | Inactive  (1.0) | Inactive  (0.83) | AGS,  TPS |
| 1-Tricosene | 5050 | 6 | Inactive  (0.75) | Inactive  (0.59) | Inactive  (0.95) | Inactive  (1.0) | Inactive  (0.77) | THS,  DYS |
| **28-Nor-17.alpha.(H)-hopane** | 3660 | 5 | Inactive  (0.88) | Inactive  (0.71) | **Active**  **(0.89)** | Inactive  (0.76) | Inactive  (0.71) | THS,  DYS |
| l-Valine, n-pentafluoropropionyl-, isobutyl ester | 1600 | 4 | Inactive  (0.77) | Inactive  (0.54) | Inactive  (0.99) | Inactive  (0.72) | Inactive  (0.71) | THS,  CSS |
| 2,6,10,14-tetramethyl | 750 | 3 | Inactive  (0.80) | Inactive  (0.74) | Inactive  (0.99) | Inactive  (0.90) | Inactive  (0.81) | NPS,  DYS |
| Behenic alcohol | 1000 | 4 | Inactive  (0.90) | Inactive  (0.5) | Inactive  (0.97) | Inactive  (1.0) | Inactive  (0.83) | NPS,  DYS |
| Octadecane | 750 | 3 | Inactive  (0.74) | Inactive  (0.58) | Inactive  (0.98) | Inactive  (1.0) | Inactive  (0.78) | NPS,  DYS |
| Ammonium acetate | 333 | 1 | Inactive  (0.84) | Inactive  (0.61) | Inactive  (0.99) | Inactive  (0.91) | Inactive  (0.75) | CSS,  NPS |

**Supplementary Table 4: Description of key metal resistance genes reported in our study**

| Metal | Gene | Function* | Mechanism |
| --- | --- | --- | --- |
| Copper (Cu) | *actP* | Copper-transporting P-type ATPase | Efflux |
|  | *copB* | Mediates copper resistance by its sequestration in the outer membrane. | Sequestration |
|  | *ricR* | Copper repressor | Transcriptional repressor metal family |
|  | *cusA/ybdE* | Cation efflux system | Efflux |
|  | *ctpV* | Copper-exporting P-type ATPase V | Export |
|  | *copA* | Encode for copper uptake ATPases | Export |
| Arsenic (As) | *pstB* | Phosphate transporting ATPase | Import |
|  | *arsM* | Arsenite S-adenosylmethyltransferase | Arsenic detoxification |
| Triclosan | *fabI* | Catalyzes the reduction of a carbon-carbon double bond in an enoyl moiety | Reduction |
|  | *mexF* | Multidrug efflux RND transporter | Efflux |
| Sodium Deoxycholate | *bepE* | Efflux pump membrane transporter | Efflux |
| Hydrochloric acid (HCl) | *rpoS* | Optimal maintenance of pH homeostasis of stationary phase cells exposed to extreme acid. | Promote the attachment of RNA polymerase to specific initiation sites |
| Ethidium Bromide | *smrA* | ABC-type multidrug efflux pump protein SmrA | Efflux |
| Zinc (Zn) | *zraR/hydH* | Transcriptional regulation | Positive auto-regulation |
| Silver (Ag) | *silP* | Silver exporting P-type ATPase | Efflux |
| Chromium (Cr), Tellurium (Te), Selenium (Se) | *ruvB* | ATP-dependent DNA helicase | Repairing DNA damage caused by chromate or its derivatives. |
| Iron (Fe) | *furA* | Transcriptional regulator | Represses transcription of the catalase-peroxidase gene |
|  | *acn* | Aconitate hydratase | Aconitase |

^*^Information taken from BacMet database

**Supplementary Table 5:** Major virulence genes found across the samples and their functional significance were identified through the VFDB database

| **Identified Sample** | **Virulence genes detected across our soil samples** | **Organisms identified in the VFDB Database** | **Function** |
| --- | --- | --- | --- |
| NPS | *prrA* | *Mycobacterium* | Probable transcriptional regulatory protein *PrrA* |
|  | *eccC5* | *Mycobacterium* | ESX-5 type VII secretion system FtsK/SpoIIIE family protein *EccC5* |
|  | *groEL2* | *Mycobacterium* | Molecular chaperone *GroEL* |
|  | *narG* | *Mycobacterium* | Nitrate reductase subunit alpha |
|  | *aftD* | *Mycobacterium* | Alpha-(1->3)-arabinofuranosyltransferase |
|  | *eccA3* | *Mycobacterium* | Type VII secretion system protein *EccA3* |
|  | *eccB5* | *Mycobacterium* | ESX-5 type VII secretion system protein *EccB5* |
|  | [*sigA/rpoV*](https://www.mgc.ac.cn/cgi-bin/VFs/gene.cgi?GeneID=VFG022860) | *Mycobacterium* | Probable RNA polymerase sigma factor *RpoD* (Sigma-A) |
|  | *SugB* | *Mycobacterium* | Probable sugar ABC transporter, permease protein *SugB* |
| AGS | *regX3* | *Mycobacterium* | Sensory transduction protein *RegX3* |
|  | *tuf* | *Mycoplasma* | Elongation factor Tu |
|  | *tufA* | *Francisella* | Elongation factor Tu |
| THS | *whiB3* | *Mycobacterium* | Putative transcriptional regulator, *WhiB* family |
|  | *mmpL3* | *Mycobacterium* | Putative membrane protein, *MmpL* |
|  | *chpA* | *Pseudomonas* | Still frameshift probable component of the chemotactic signal transduction system |
|  | *pks* | *Mycobacterium* | Probable polyketide synthase |
|  | *fliA* | *Aeromonas* | Flagellar biosynthesis sigma factor |
|  | *fadD26* | *Mycobacterium* | Long-chain-fatty-acid--AMP ligase *FAAL26/FadD26* |
|  | *clpC* | *Listeria* | Endopeptidase *Clp* ATP-binding chain C |
|  | *algC* | *Pseudomonas* | Phosphomannomutase *AlgC* |
|  | [*sigA/rpoV*](https://www.mgc.ac.cn/cgi-bin/VFs/gene.cgi?GeneID=VFG022860) | *Mycobacterium* | Probable RNA polymerase sigma factor *RpoD* (Sigma-A) |
|  | *SugB* | *Mycobacterium* | Probable sugar ABC transporter, permease protein *SugB* |
| CSS | *entB* | *Klebsiella* | 2,3-dihydro-2,3-dihydroxybenzoate synthetase, isochroismatase |
|  | *entE* | *Acinetobacter* | Non-ribosomal peptide synthetase adenylate-forming enzyme of acinetobactin synthesis |
|  | *ybtP* | *Klebsiella* | *Yersiniabactin* ABC transporter ATP-binding/permease protein *YbtP* |
|  | *acpXL* | *Brucella* | Acyl carrier protein |
|  | *gspL* | *Acinetobacter* | General secretion pathway protein L |
|  | *cheY* | *Burkholderia* | Chemotaxis protein *CheY* |
|  | *entF* | *Klebsiella* | Enterobactin synthase subunit F |
|  | *fimF* | *Aeromonas* | Fimbrial protein |
| DYS | *bap* | *Acinetobacter* | Biofilm-associated protein |
|  | *PA3349* | *Pseudomonas* | Chemotaxis protein |
|  | *regX3* | *Mycobacterium* | Sensory transduction protein *RegX3* |
|  | *icl* | *Mycobacterium* | Isocitrate lyase *Icl* (isocitrase) (isocitratase) |
|  | *fbpC* | *Mycobacterium* | Secreted antigen 85-C *FbpC* (85C) (antigen 85 complex C) (AG58C) (mycolyl transferase 85C) (fibronectin-binding protein C) |
| TMS | *irp1* | *Klebsiella* | Yersiniabactin polyketide synthase HMWP1 |
|  | *wbdA* | *Brucella* | Mannosyltransferase |
|  | *bpsC* | *Bacillus* | UTP--glucose-1-phosphate uridylyltransferase |
|  | *entC* | *Klebsiella* | Isochorismate synthase |
|  | *espR* | *Mycobacterium* | ESX-1 transcriptional regulator *EspR* |
|  | *fepA* | *Klebsiella* | Outer membrane receptor *FepA* |
|  | *fimB* | *Bordetella* | Chaperone protein |
|  | *tufA* | *Francisella* | Elongation factor Tu |
| TPS | *cap8E* | *Staphylococcus* | Type 8 capsular polysaccharide synthesis protein *Cap8E* |
|  | *cap8D* | *Staphylococcus* | Type 8 capsular polysaccharide synthesis protein *Cap8D* |
|  | [*sigA/rpoV*](https://www.mgc.ac.cn/cgi-bin/VFs/gene.cgi?GeneID=VFG022860) | *Mycobacterium* | Probable RNA polymerase sigma factor *RpoD* (Sigma-A) |
| PIS | [*sigA/rpoV*](https://www.mgc.ac.cn/cgi-bin/VFs/gene.cgi?GeneID=VFG022860) | *Mycobacterium* | Probable RNA polymerase sigma factor *RpoD* (Sigma-A) |

A


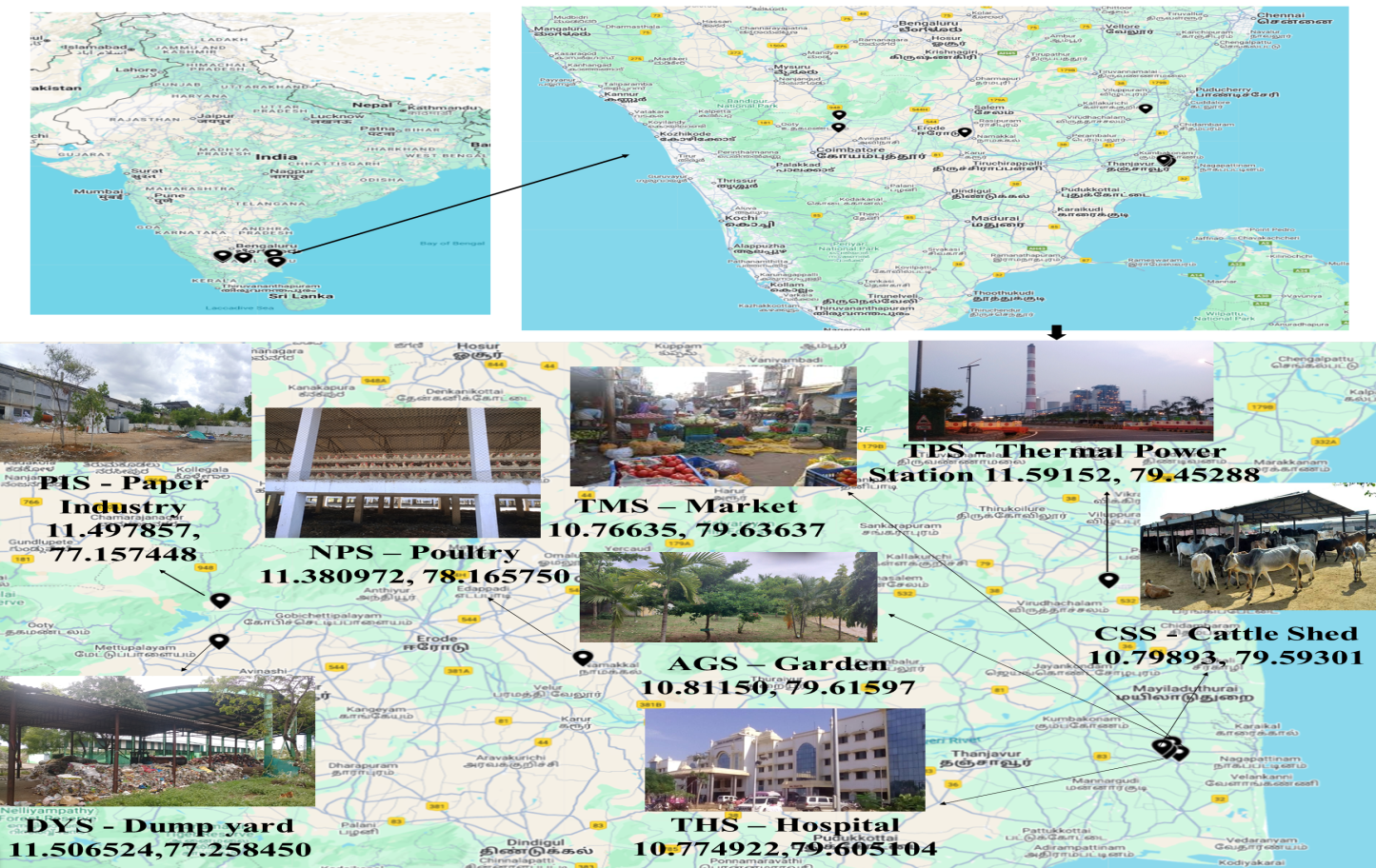


**B**


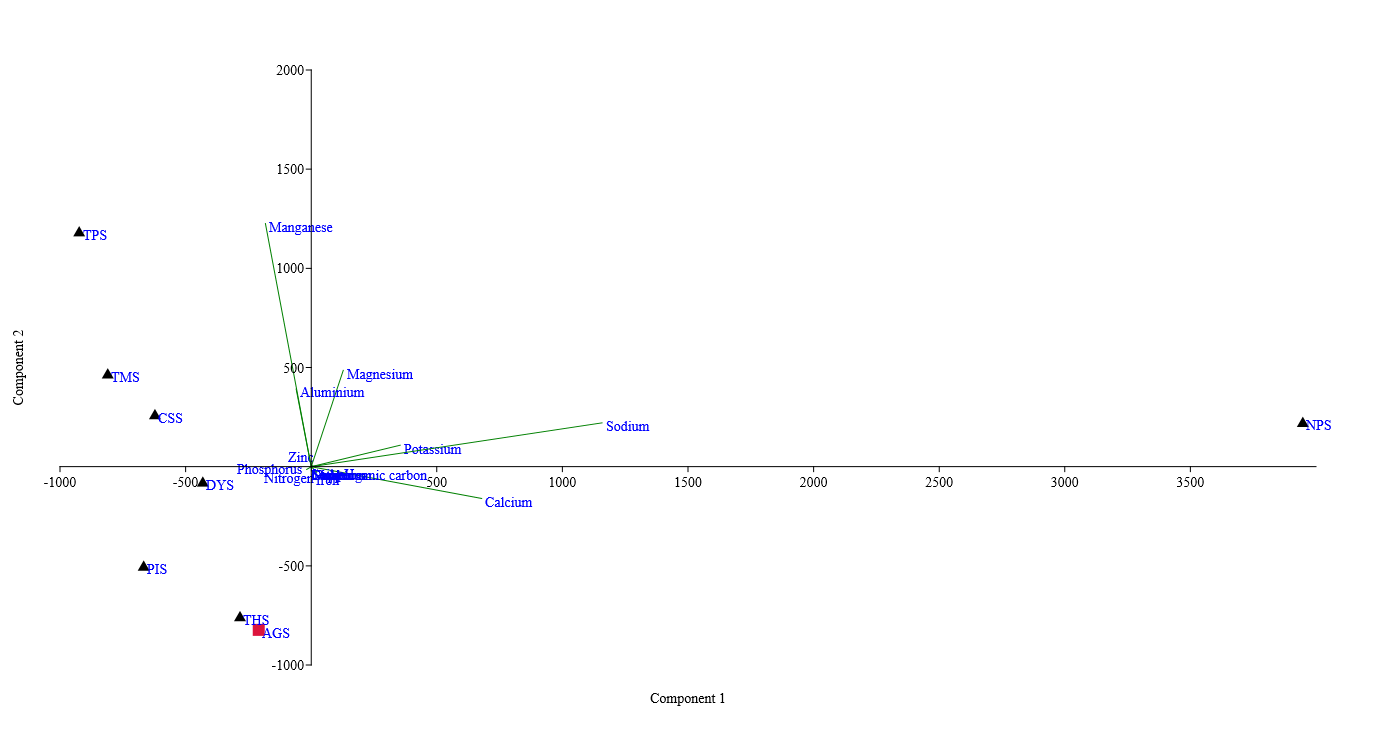


**C**


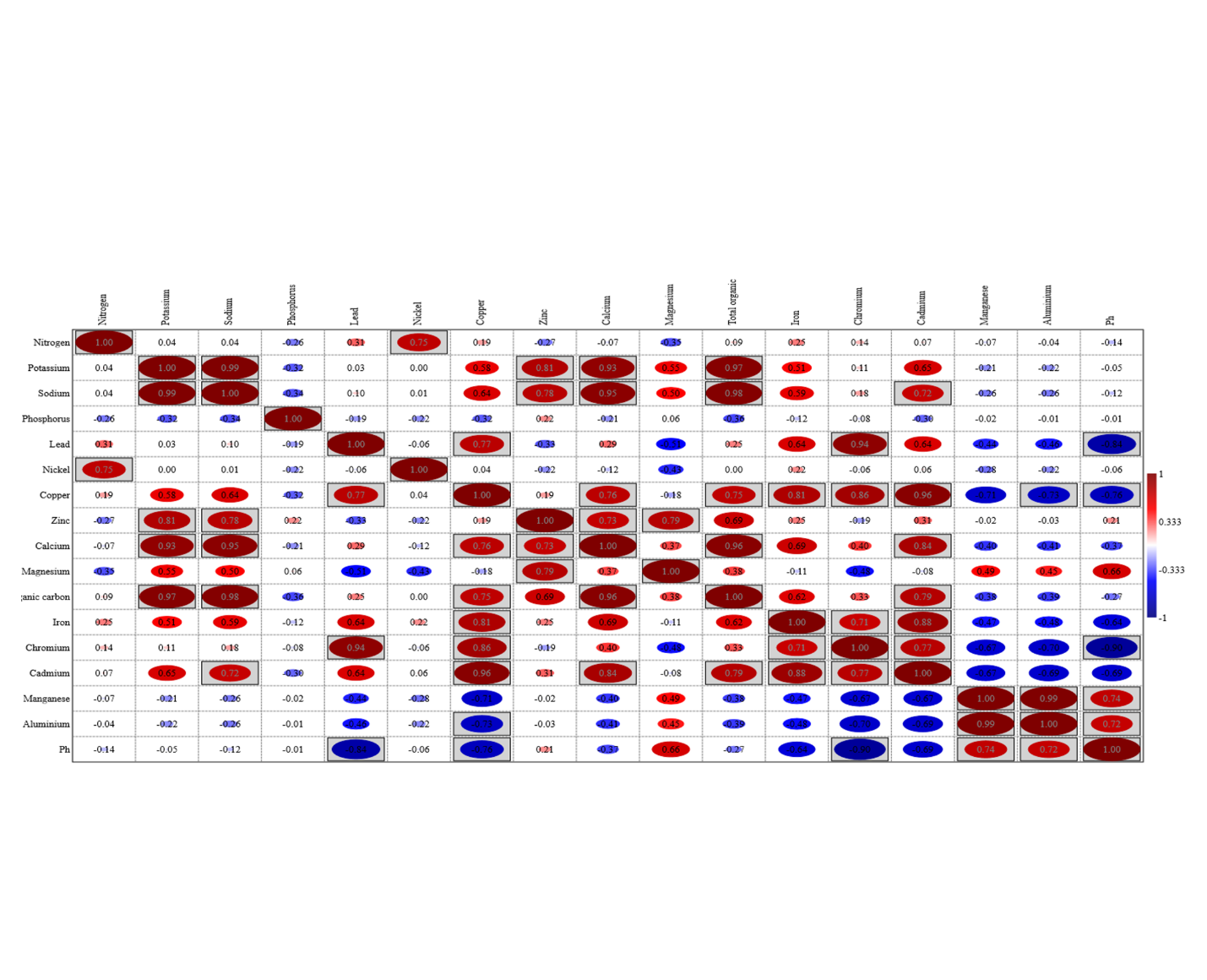


**Supplementary Fig.1: (A)** Sample collection site **(B)** Scatter plot for Principal Component Analysis (PCA) of soil physiochemical properties and heavy metal contents for soil samples using PAST 4.03 software. (**C**) Correlation plot depicting positive and negative correlation between soil physicochemical characteristics. The colour and size are mapped to the Pearson correlation coefficient values. The stronger the correlations are, the larger the circle. Positive correlations are displayed in red and negative ones in blue. All the statistically significant correlations (P-values <0.05) were highlighted in square boxes.


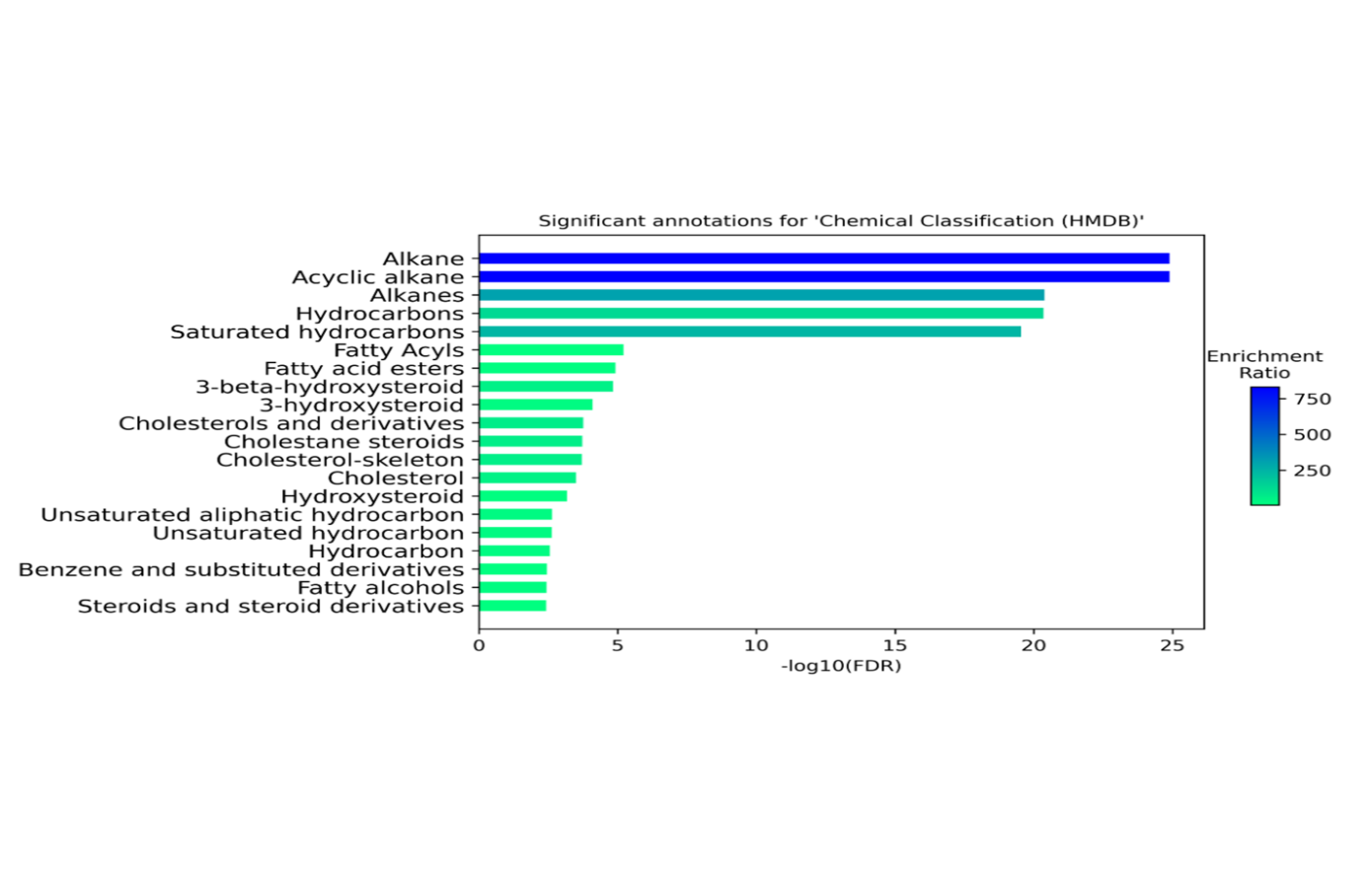


**Supplementary Fig.2**: Illustrations of the enrichment analysis based on the chemical classification based on Human Metabolome Database (HMDB) performed using MBROLE3. Bar plotPlots with FDR <0.05 and a maximum of 20 annotations, along with the enrichment ratio, are displayed.


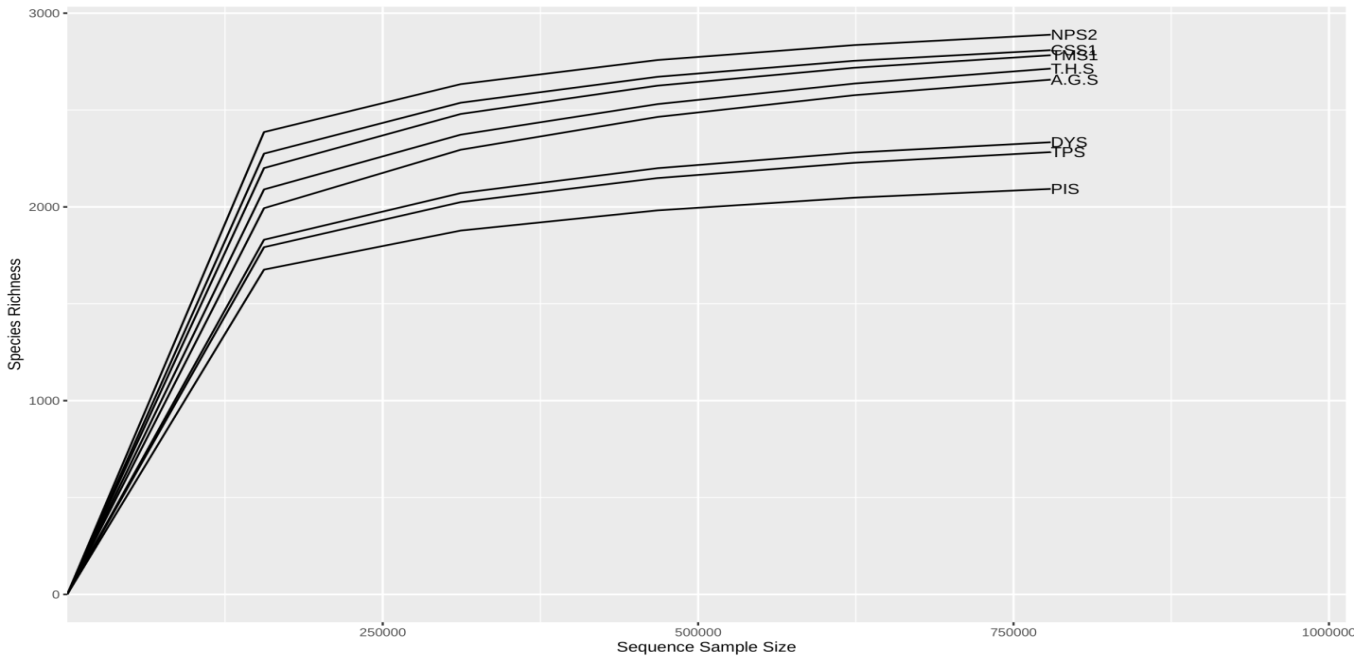


**Supplementary Fig.3:** Rarefaction curves analysis of eight samples. X-axis indicates sequence size of samples and Y axis indicates species richness.


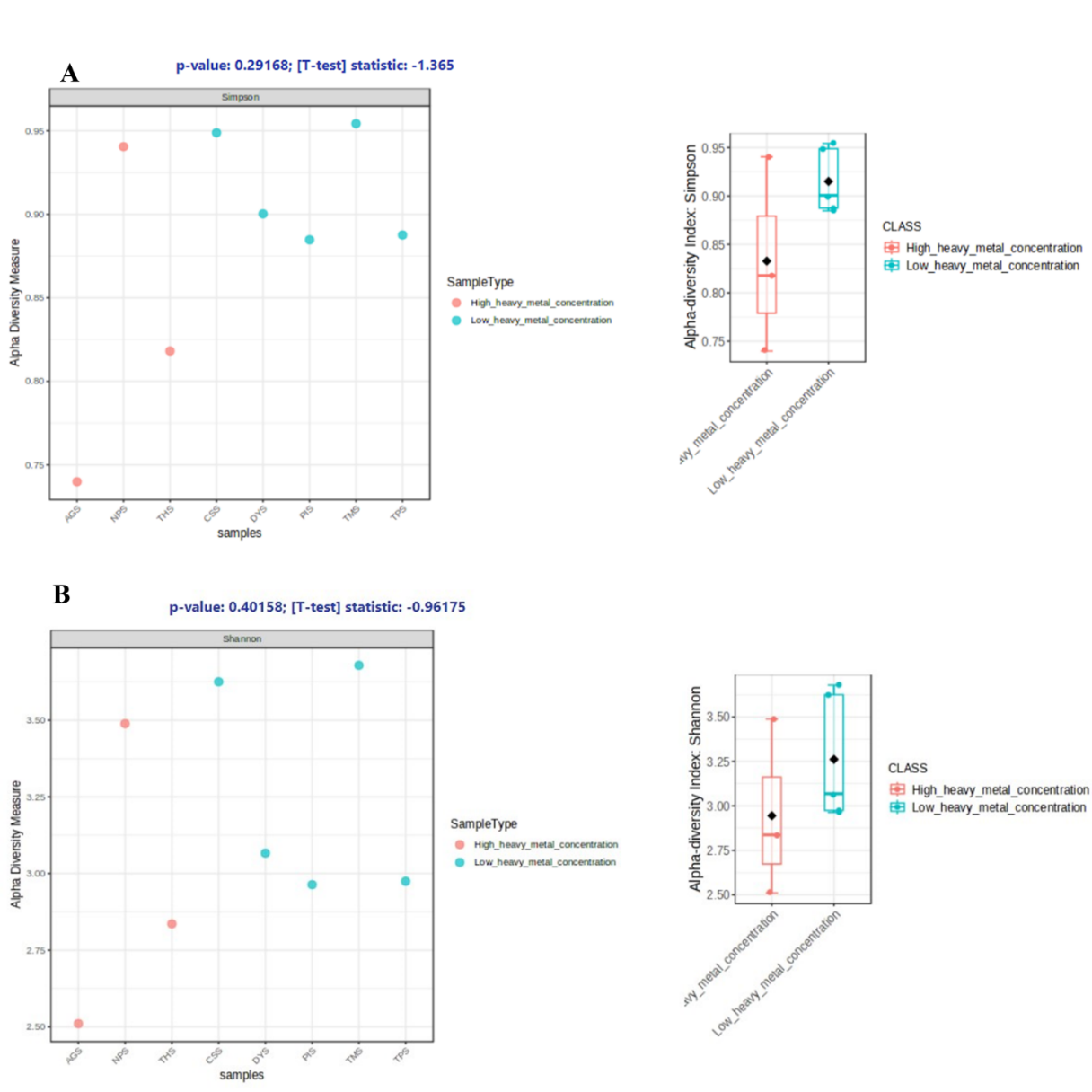


**Supplementary Fig.4:** Alpha diversity indices showing bacterial diversity across the samples: (A) Simpson index B) Shannon index. Welch T-test was applied as the statistical method.

**A**


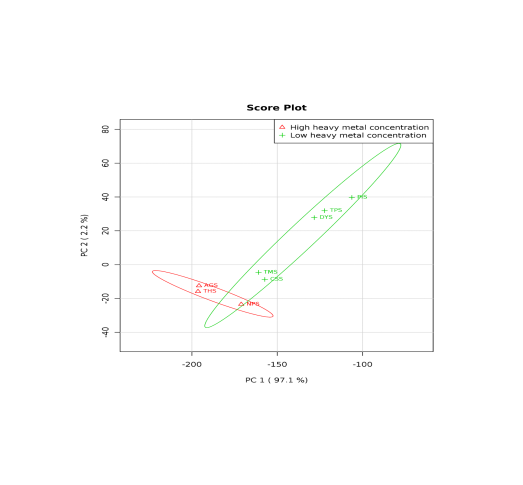


**B**


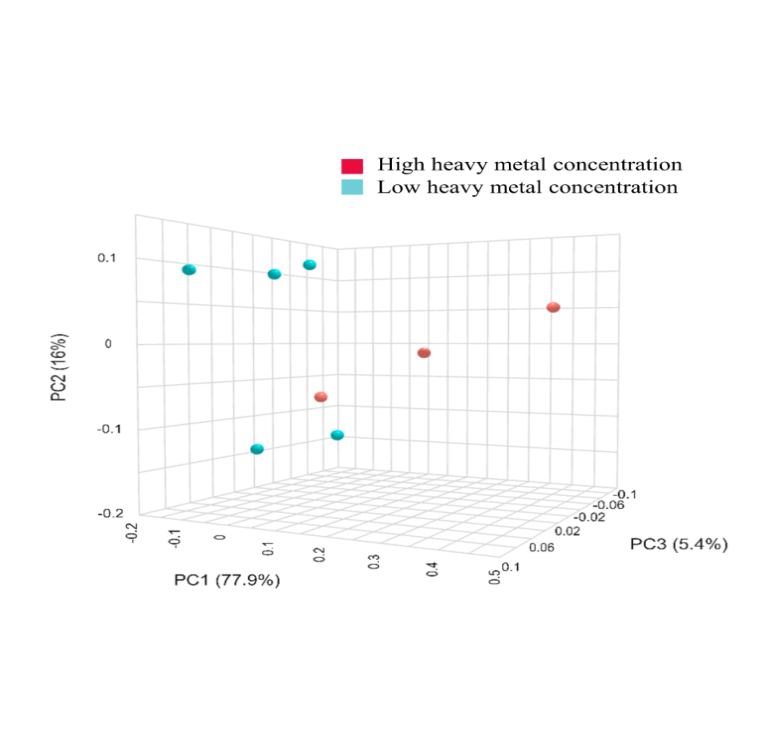


**C**


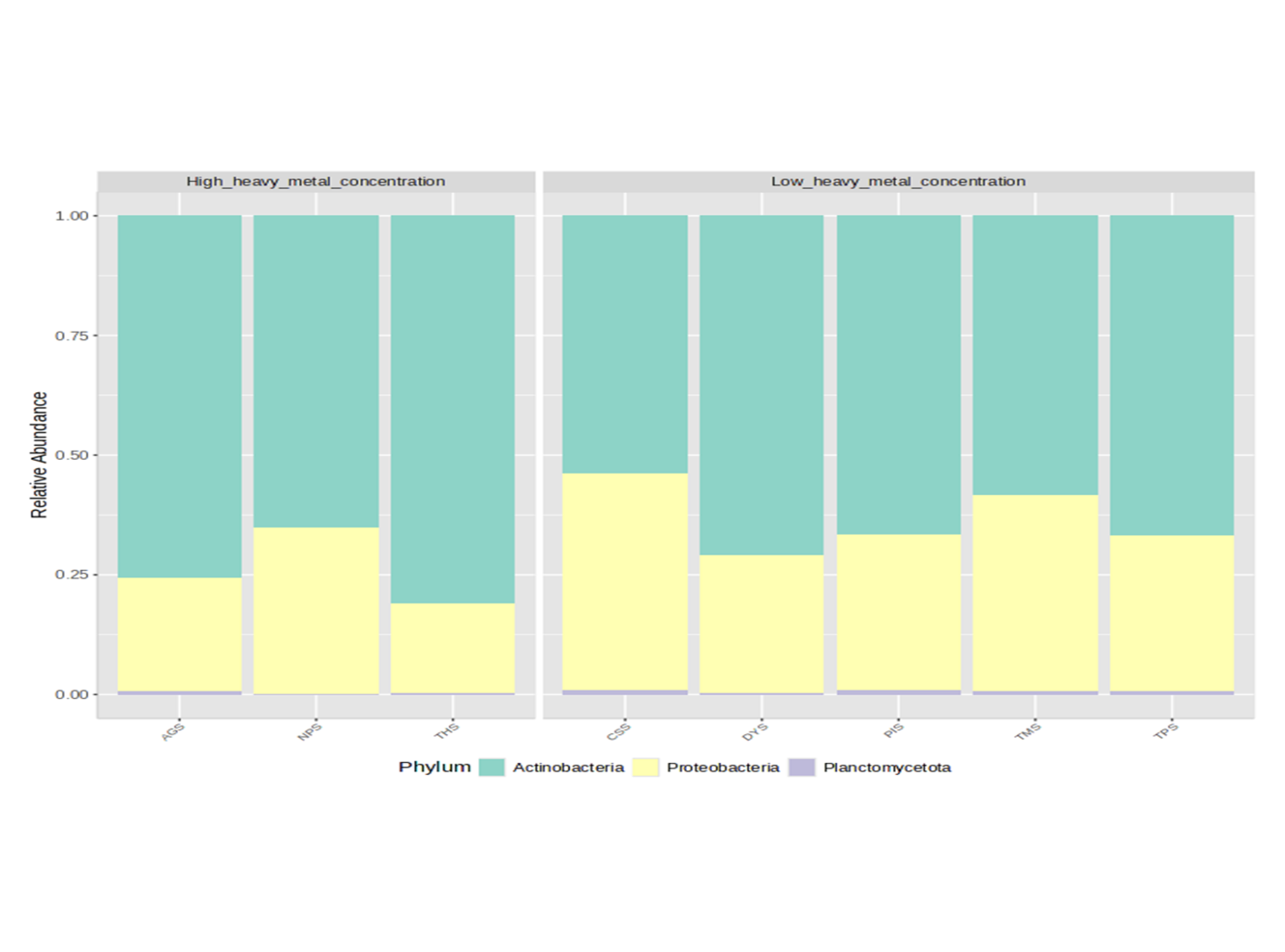


**D**

**
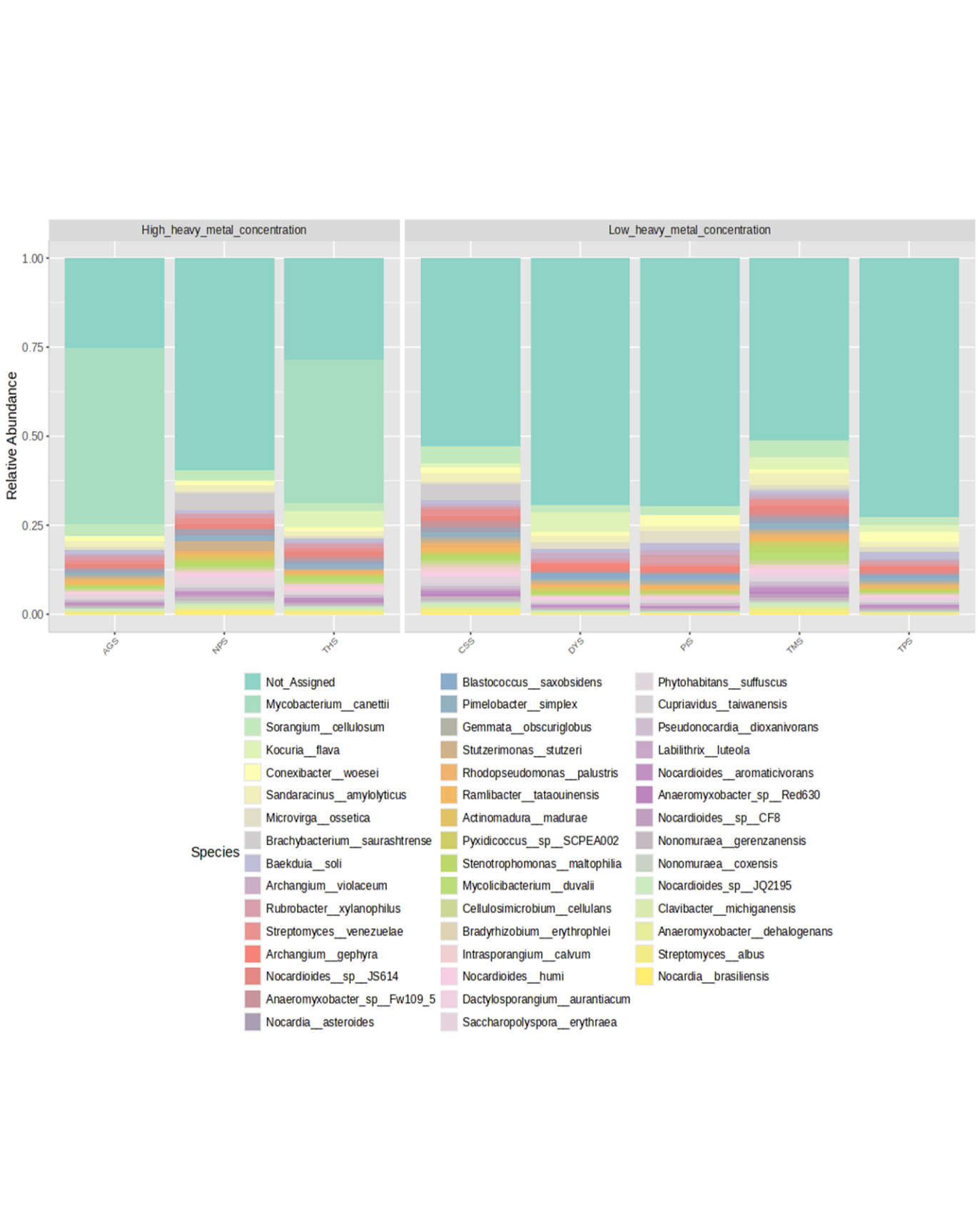
**

**Supplementary Fig.5:** Principal Component Analysis (PCA) by PCA score plot (A) and PCA 3D plot (B). (C) The stacked bar plot displays the Bacterial abundance at the phylum level across samples collected from eight sites. AGS, NPS, and THS correspond to samples with high heavy metal concentrations, whereas CSS, DYS, PIS, TMS, and TPS represent samples with low heavy metal concentrations. (D) A stacked bar plot depicting the abundance of bacterial species across the samples. The sites are categorized based on heavy metal concentrations.


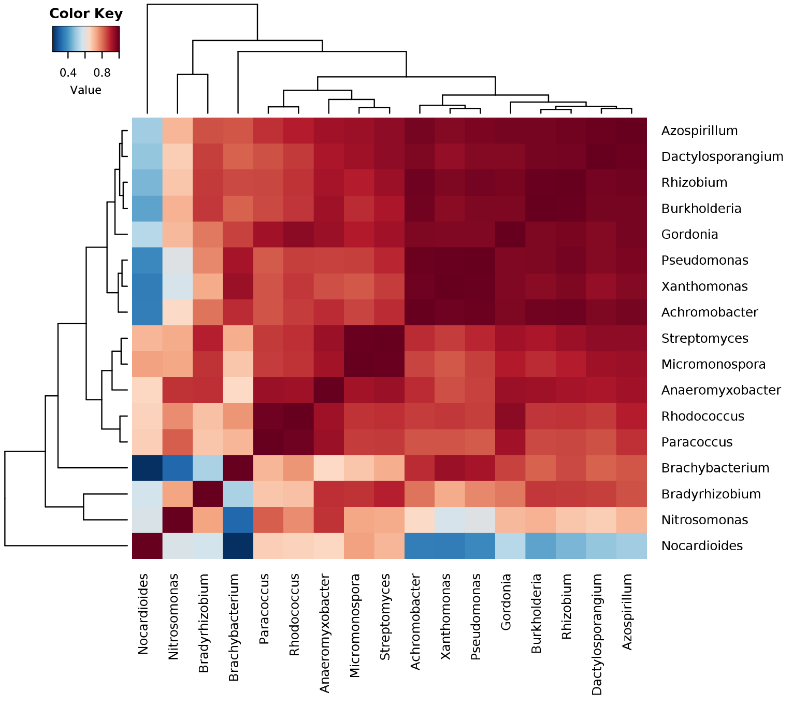


**Supplementary Fig.6**: Heat map depicting the Pearson correlation between Bacterial - genus level.

**A**


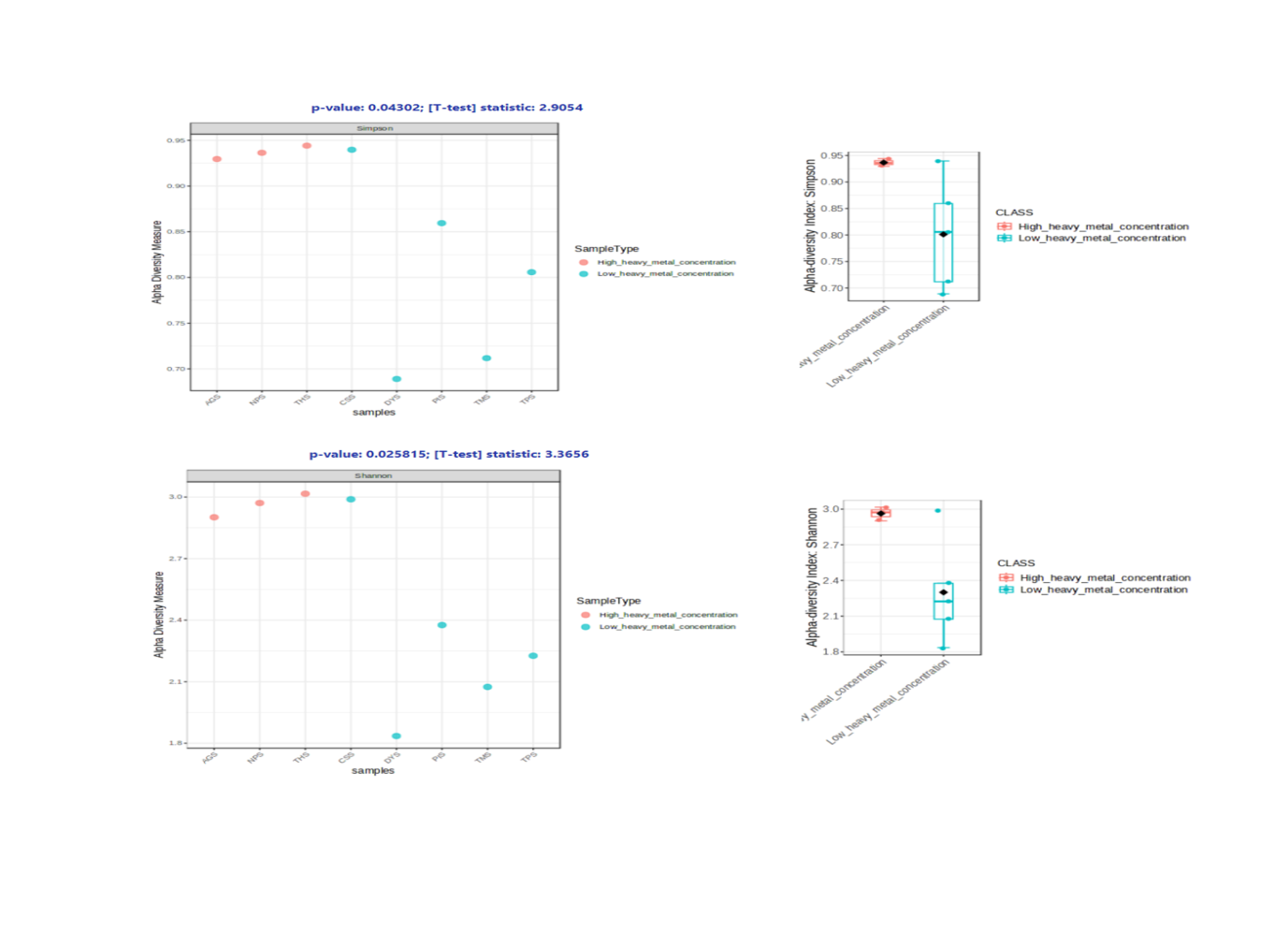


**B**


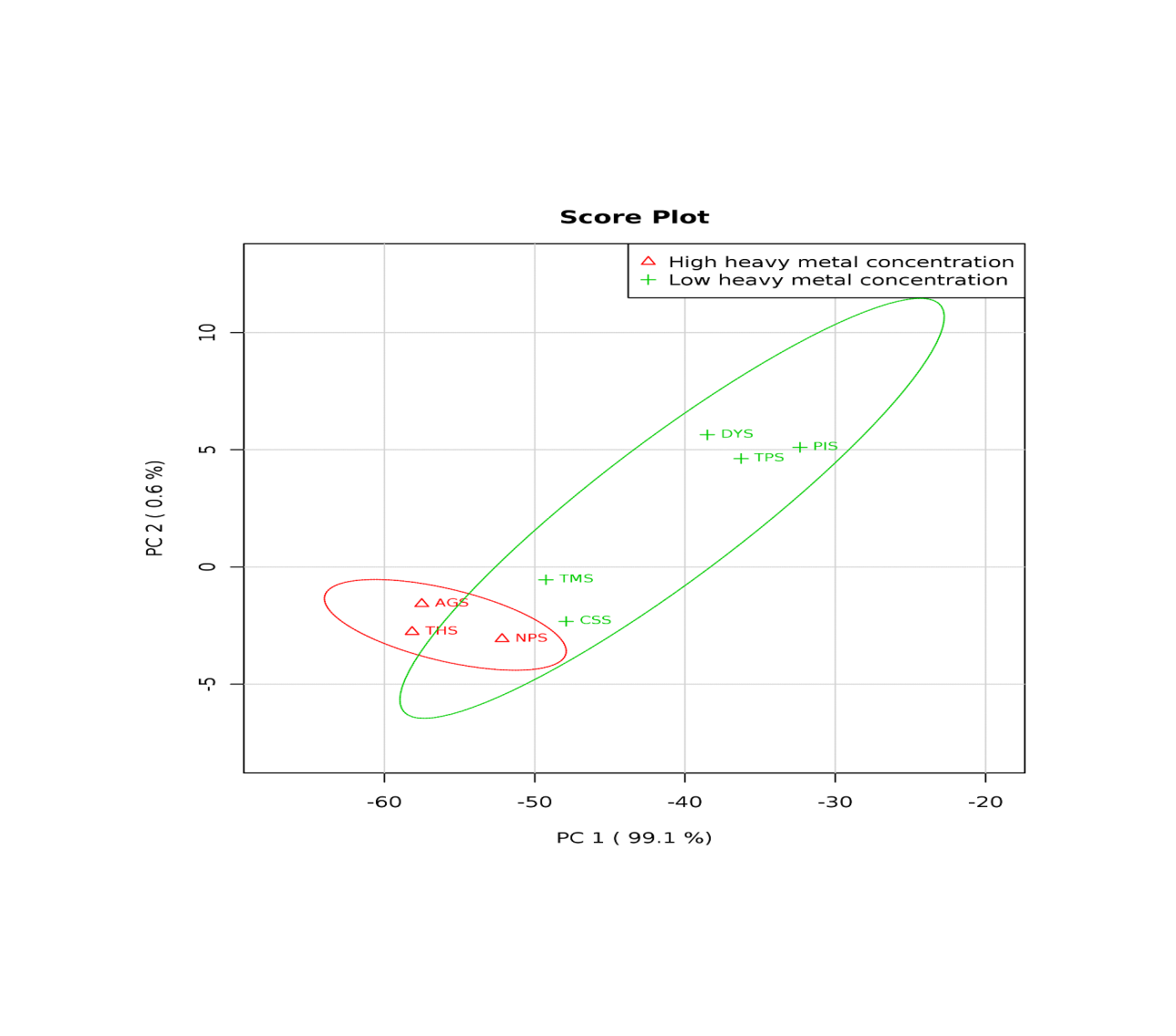


**C**


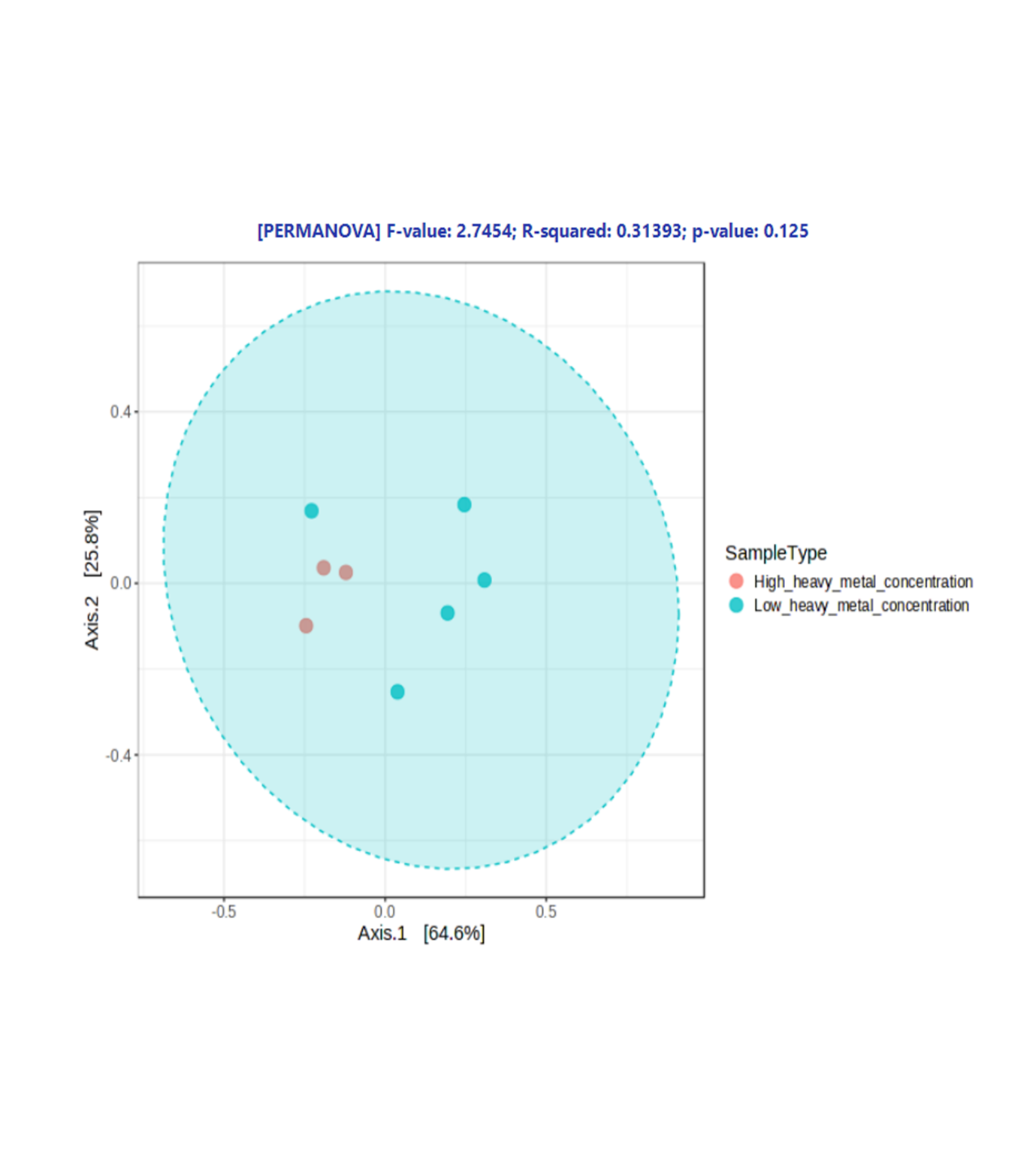


**Supplementary Fig.7**: (A) Alpha diversity indices of archaea across the samples; (B) Principal Component Analysis (PCA) by score plot; (C) Beta diversity of archaea by using Microbiome Analyst 2.0 (Ordination method: PCoA, distance method: Bray-Curtis index and statistical method, PERMANOVA, were selected).

**A**


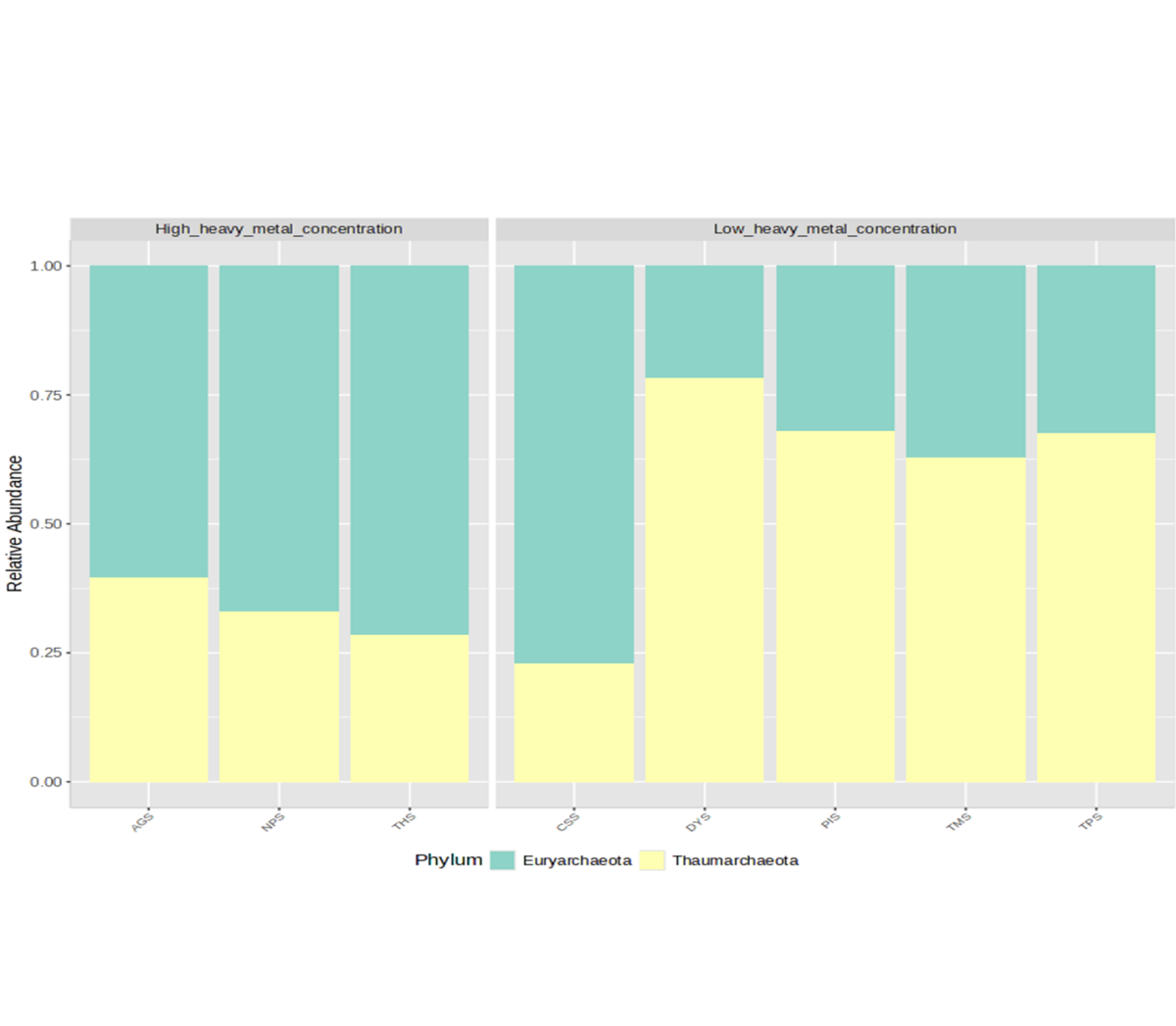


**B**


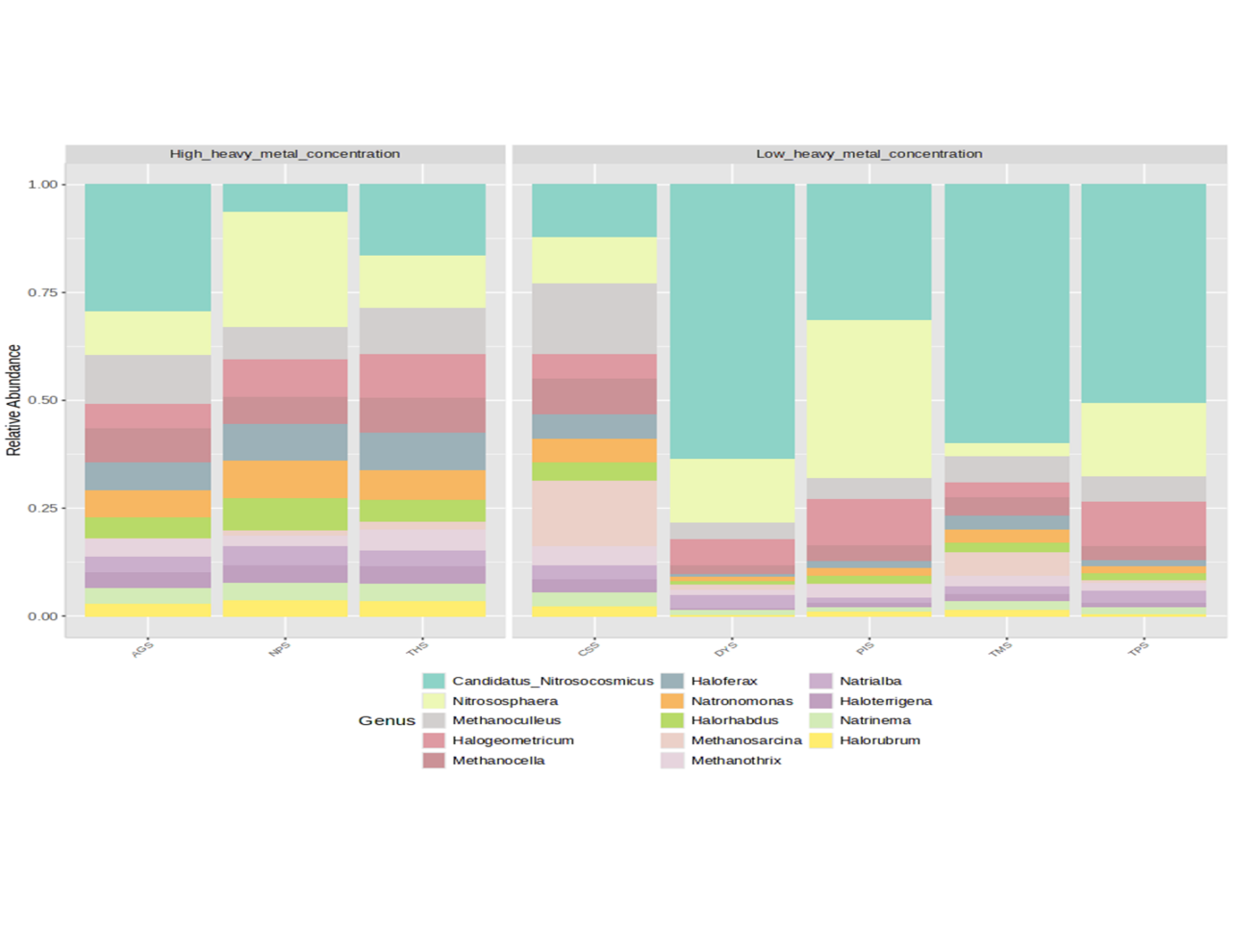


**C**


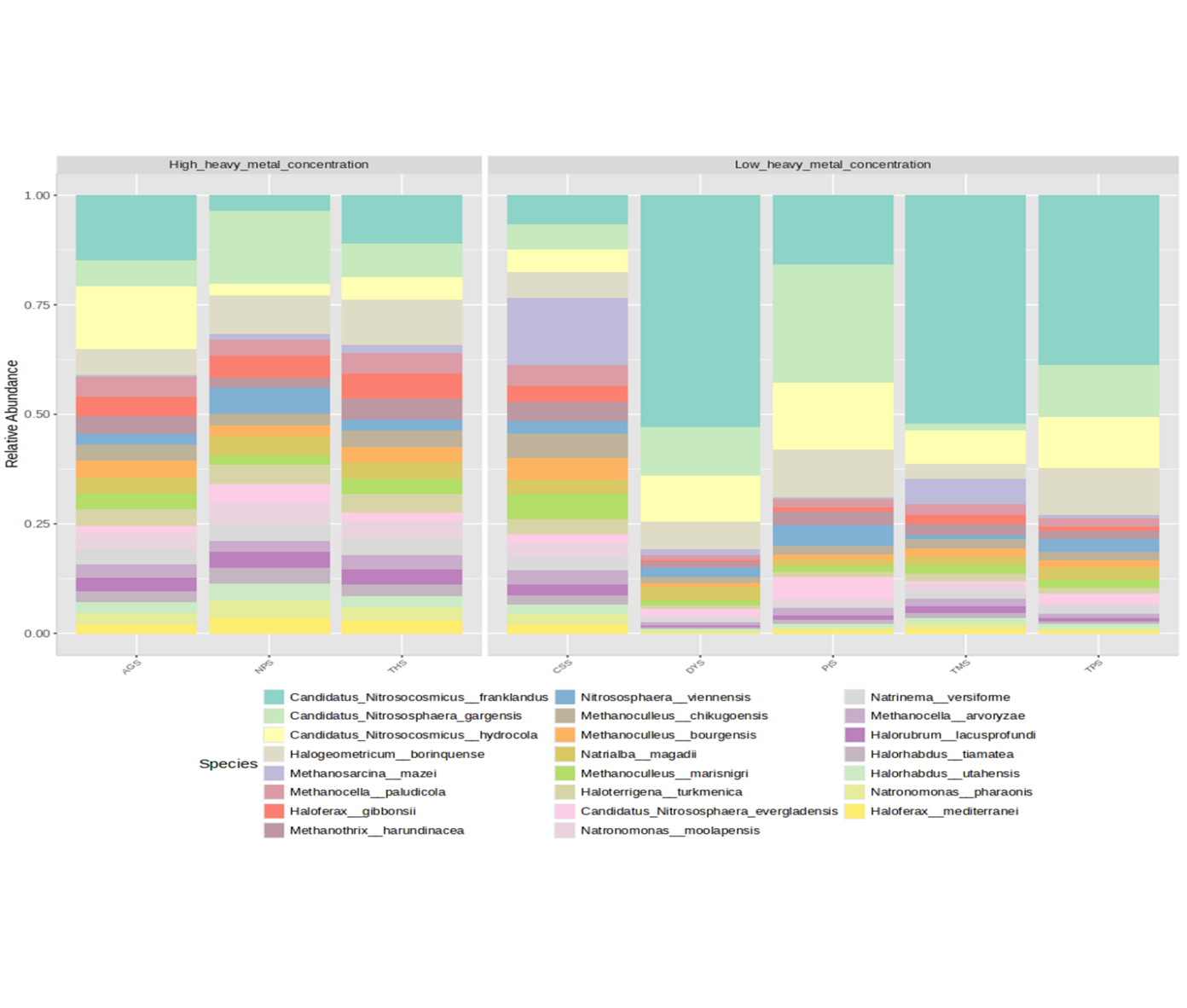


**Supplementary Fig.8**: (A) The stacked bar plot displays the archaeal abundance across samples collected from eight sites. AGS, NPS, and THS correspond to samples with high heavy metal concentration, while CSS, DYS, PIS, TMS, and TPS represent samples with low heavy metal concentrations. Stacked bar plot showing the archaeal abundance at the genus (B) and species (C) level across all the samples;


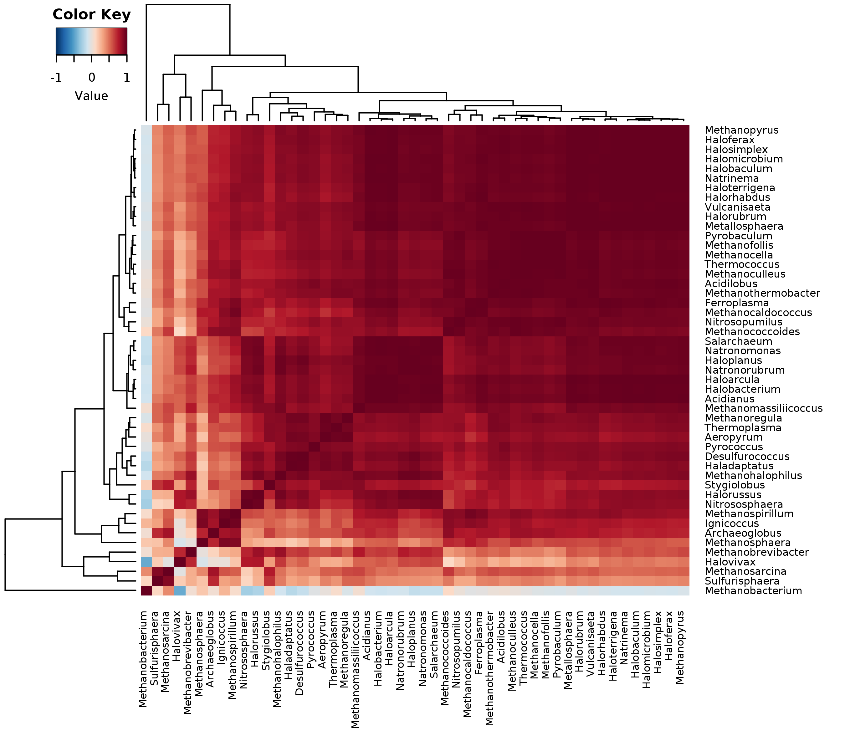


**Supplementary Fig.9**: Heat map depicting the Pearson correlation between archaeal - genus level. Archaeal genera were positively correlated with each other.

**A**


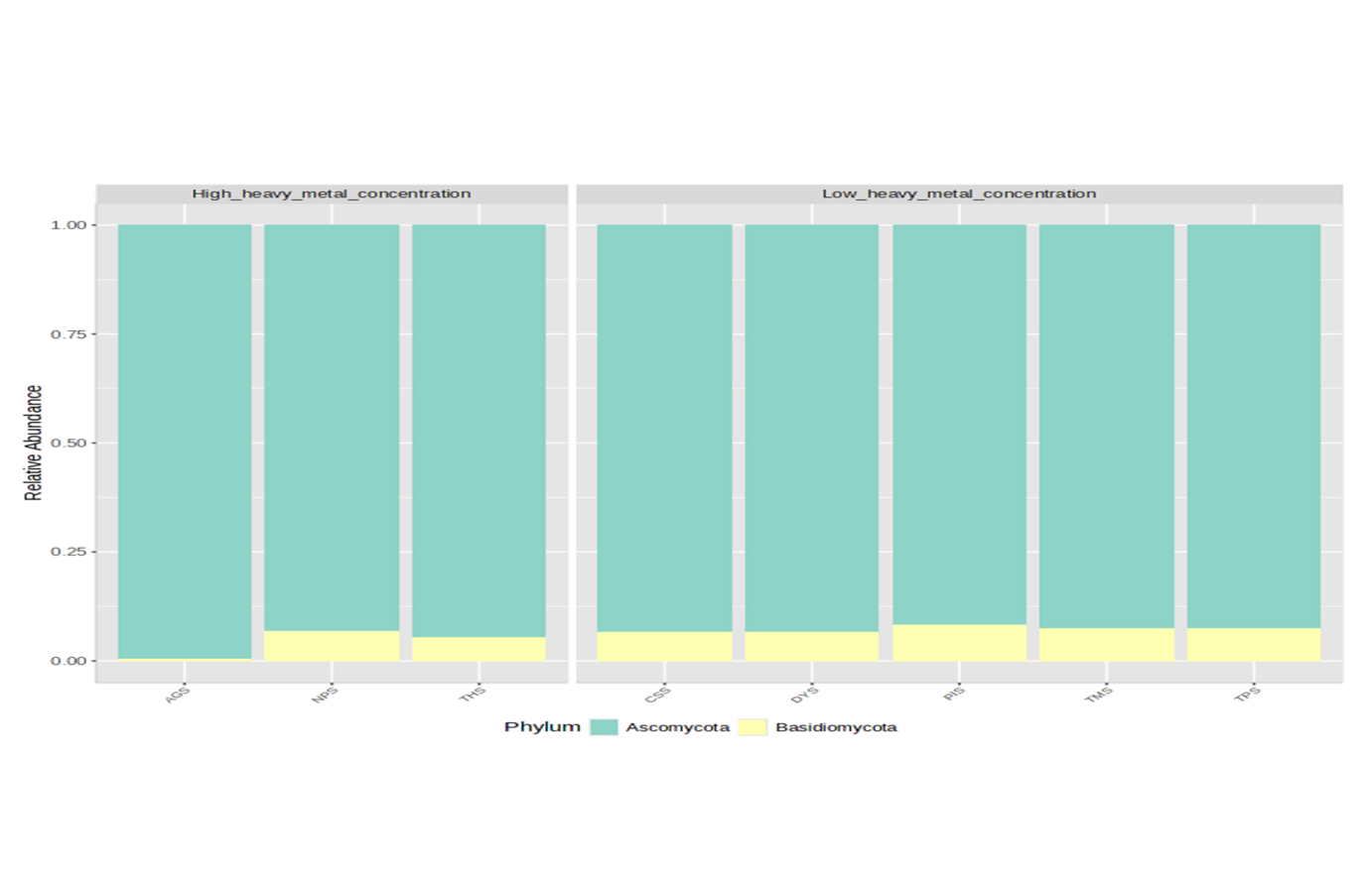


**B**

**
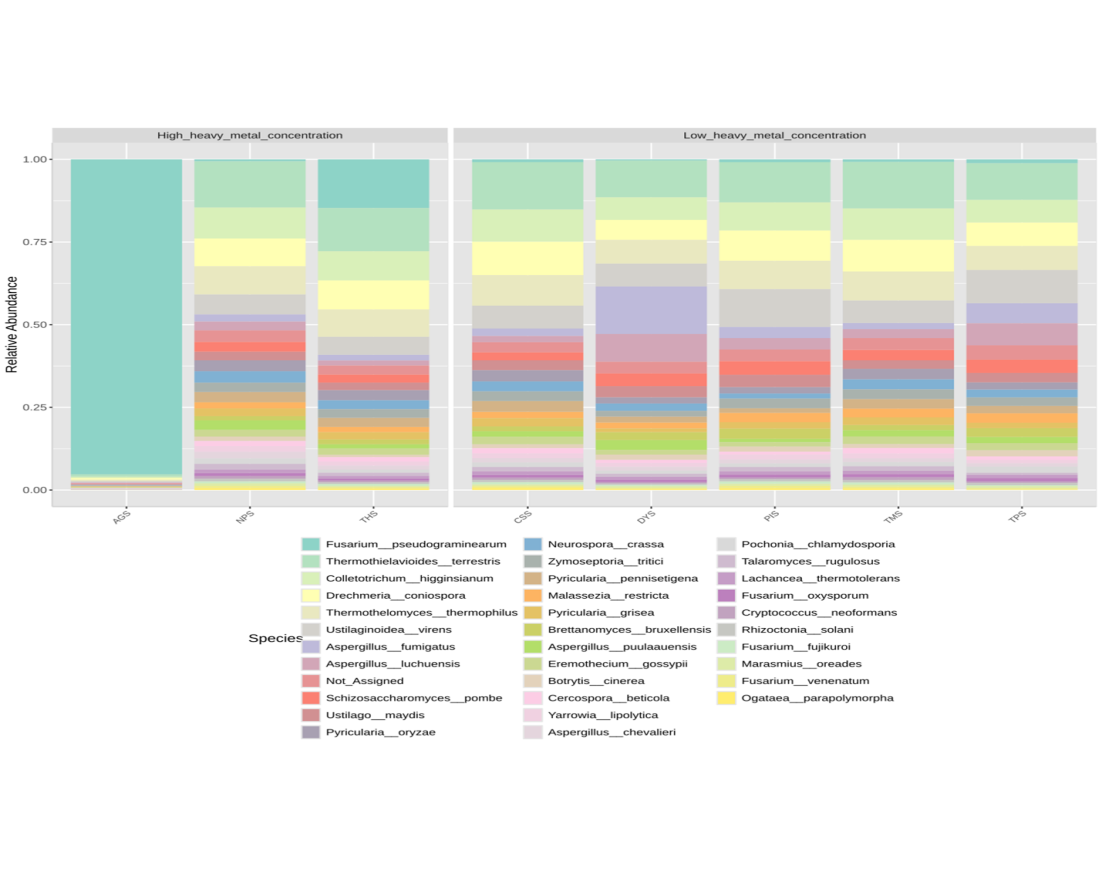
**

**C**

**
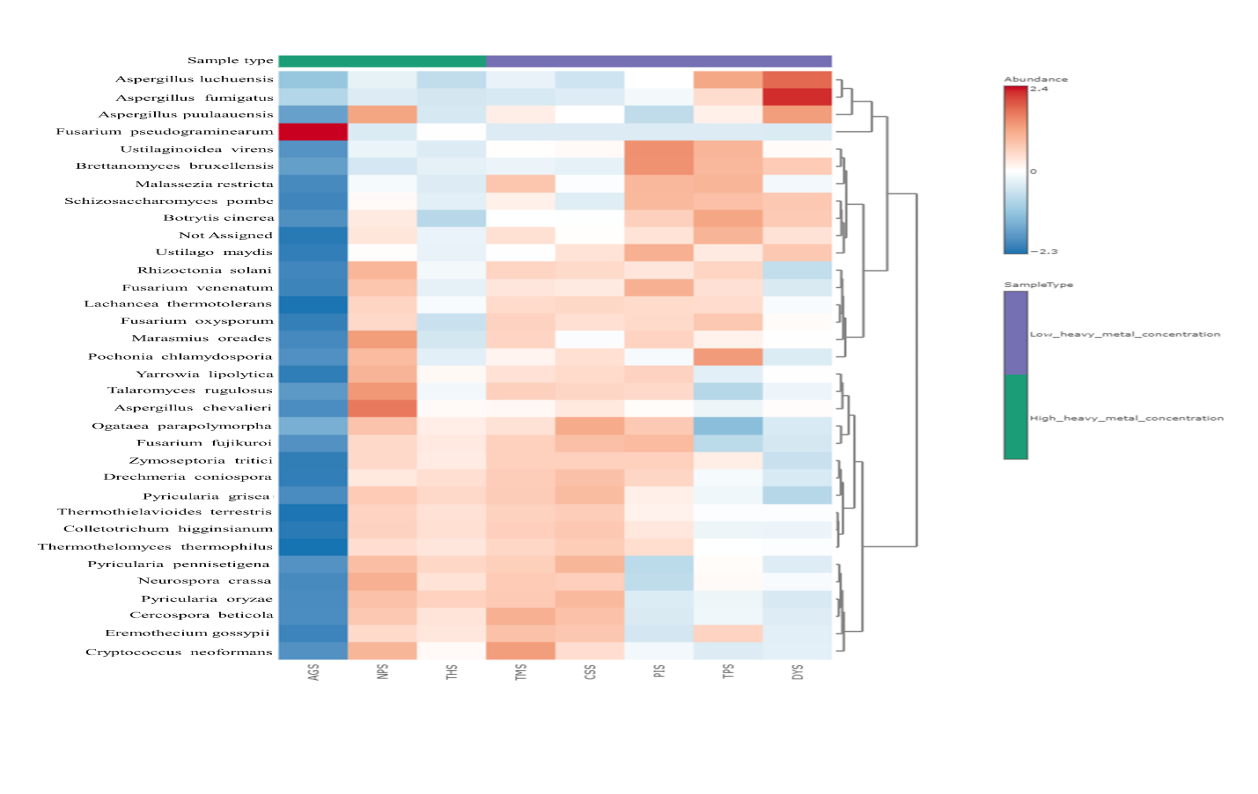
**

**Supplementary Fig.10**: (A) The stacked bar plot displays the fungal abundance across samples collected from eight sites. AGS, NPS, and THS correspond to samples with high heavy metal concentrations, while CSS, DYS, PIS, TMS, and TPS represent samples with low heavy metal concentrations at the Phylum level. (B) Stacked bar plot showing species-level fungal abundance across all the HHMC and LHMC samples; (C) Heat map illustrating the fungal abundance across all the samples.

A


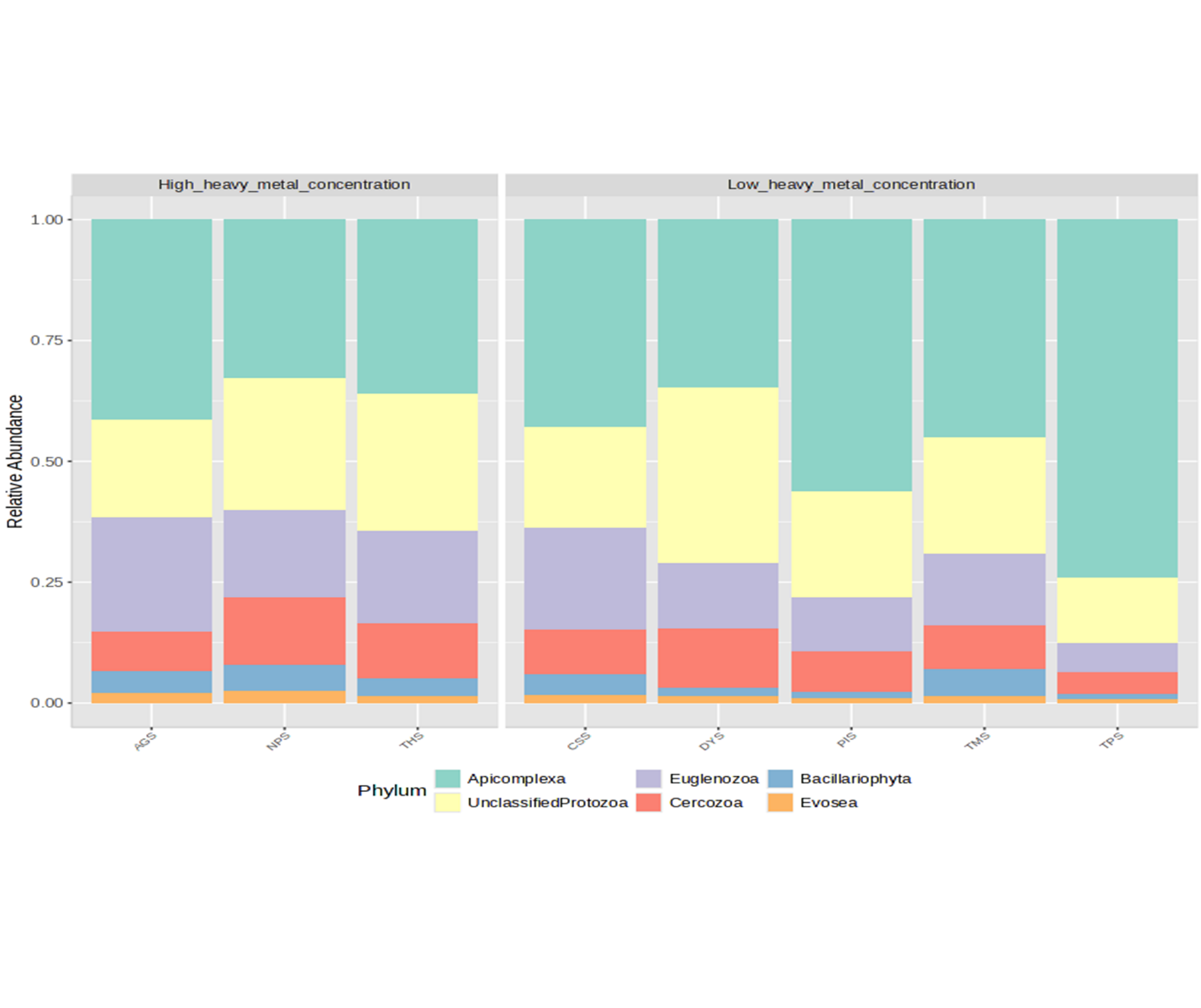


**B**


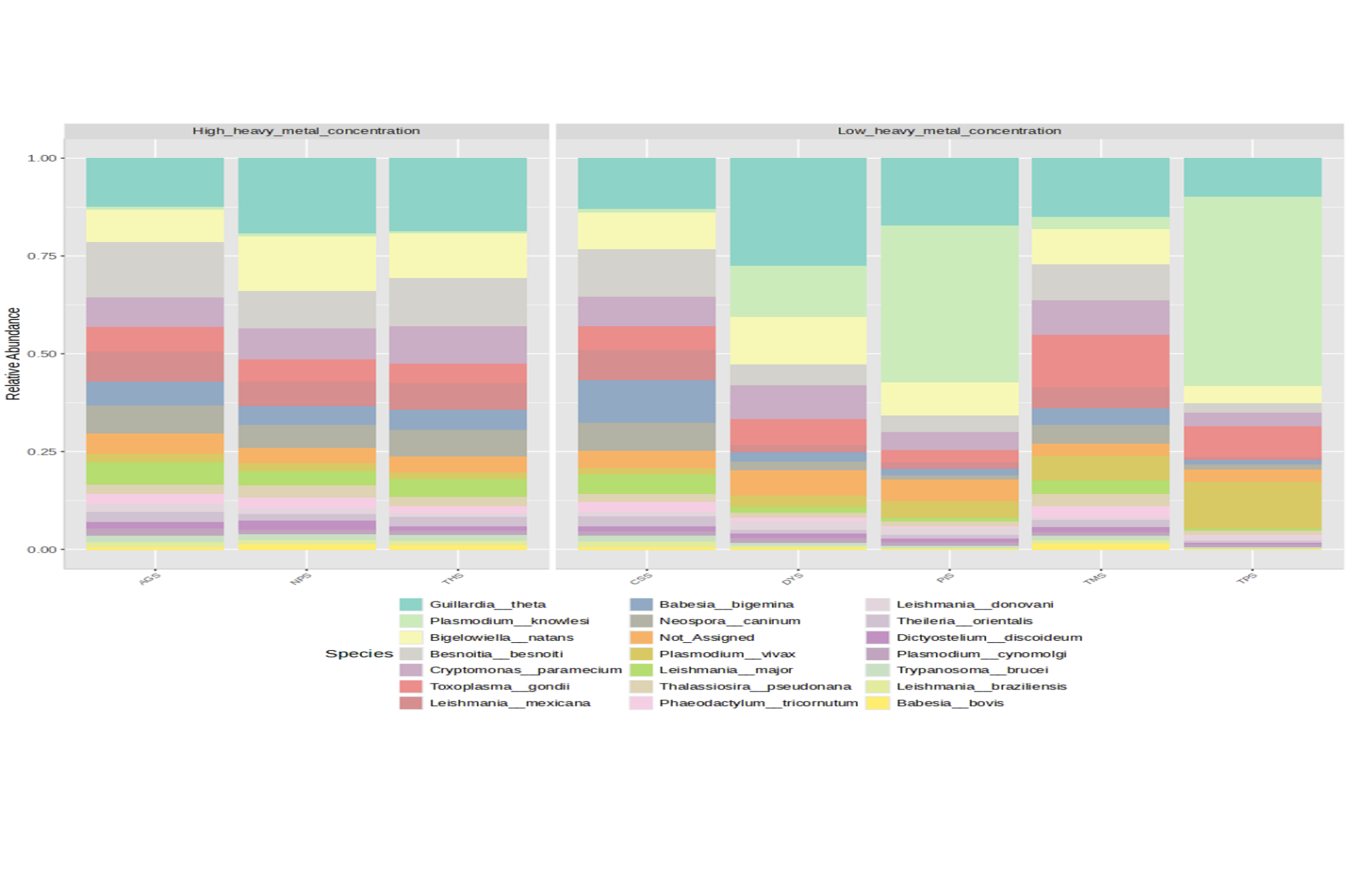


**Supplementary Fig.11**: (A) Stacked bar plot depicting the protozoan abundance across the HHMC and LHMC samples. (B) A stacked bar plot depicting the protozoan abundance at the species level across all the samples.


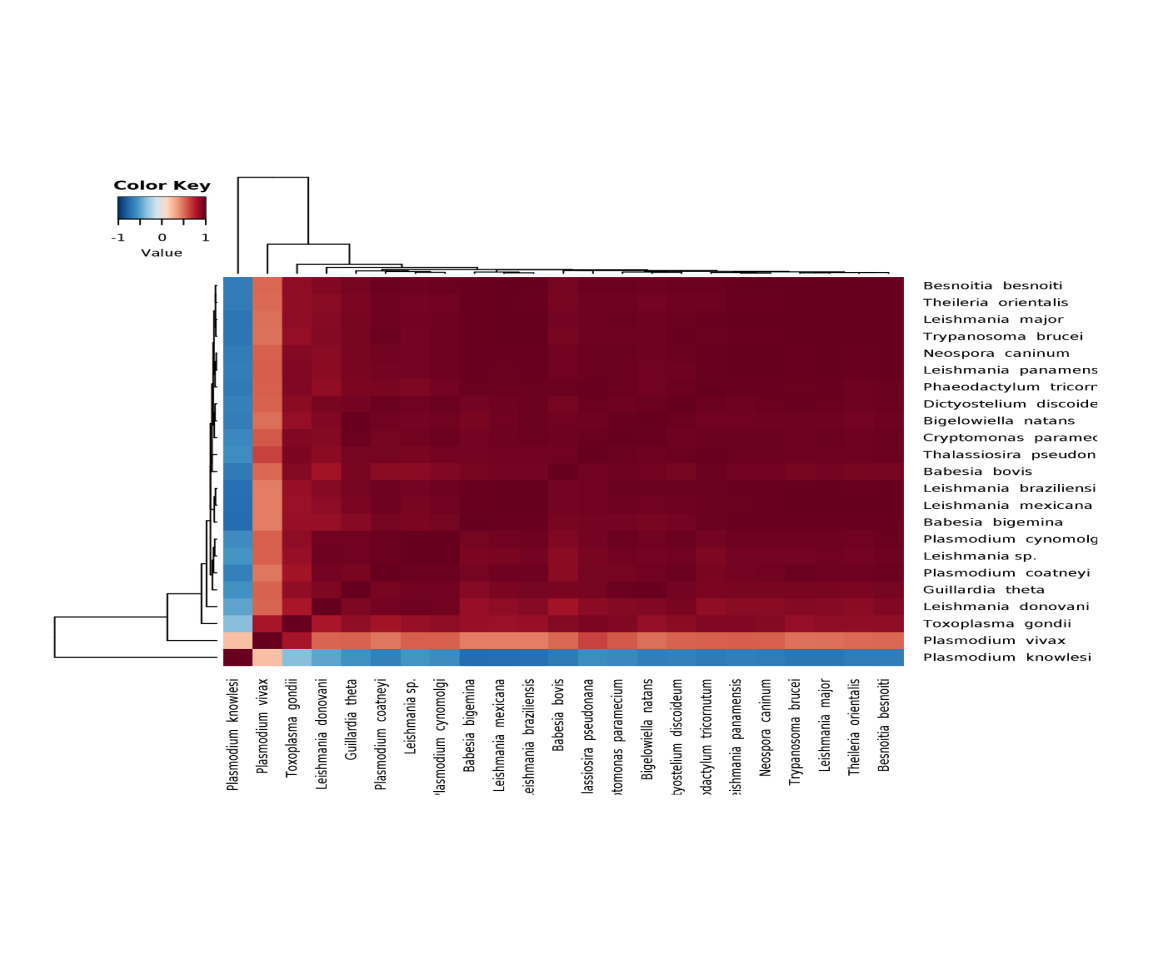


**Supplementary Fig.12:** Heat map depicting the Pearson correlation between protozoan species level.


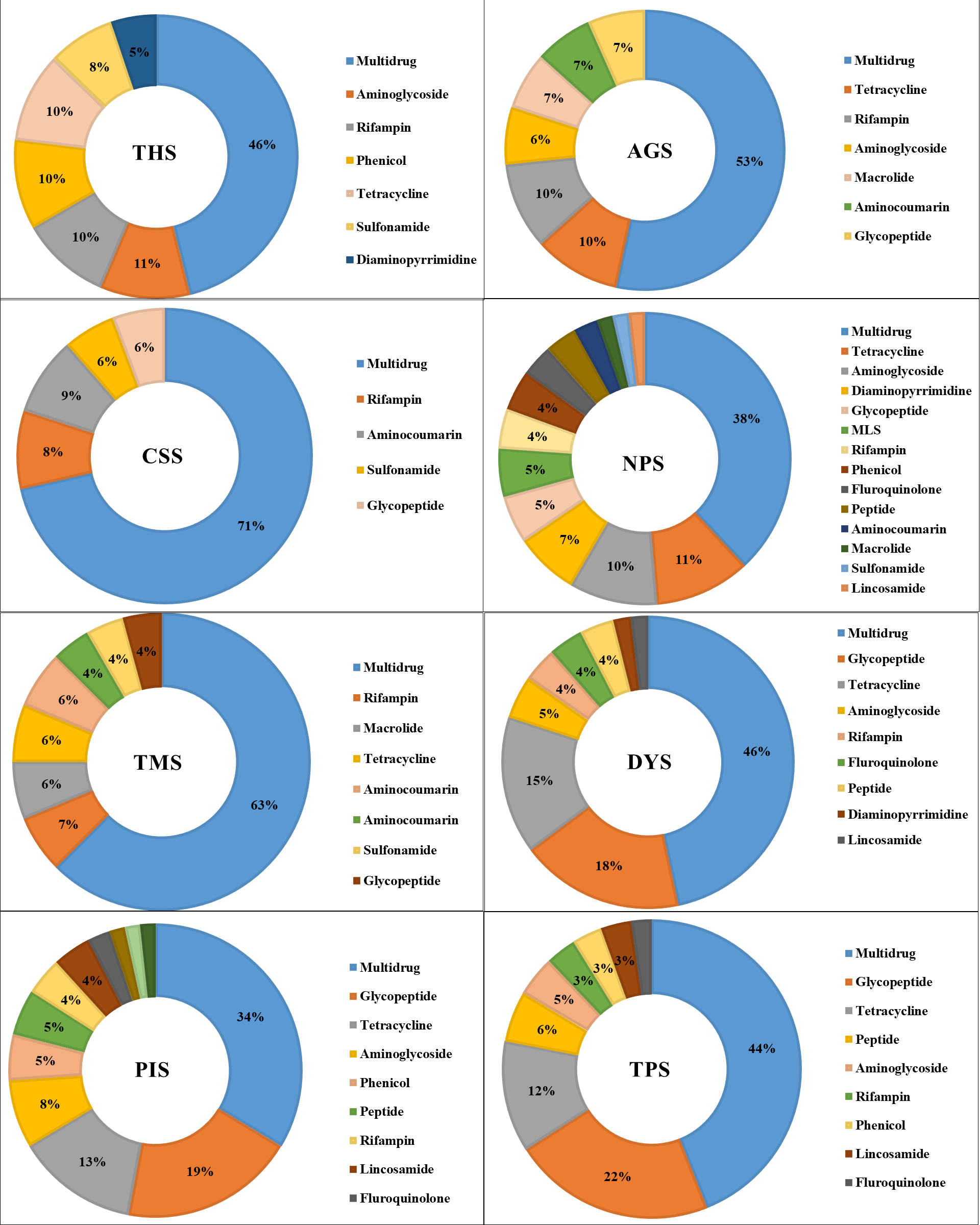
**Supplementary Fig.13:** The abundances and the counts of ARGs for each antimicrobial across eight soil samples sites. All ARGs were categorized according to the antibiotic against which they demonstrated resistance.


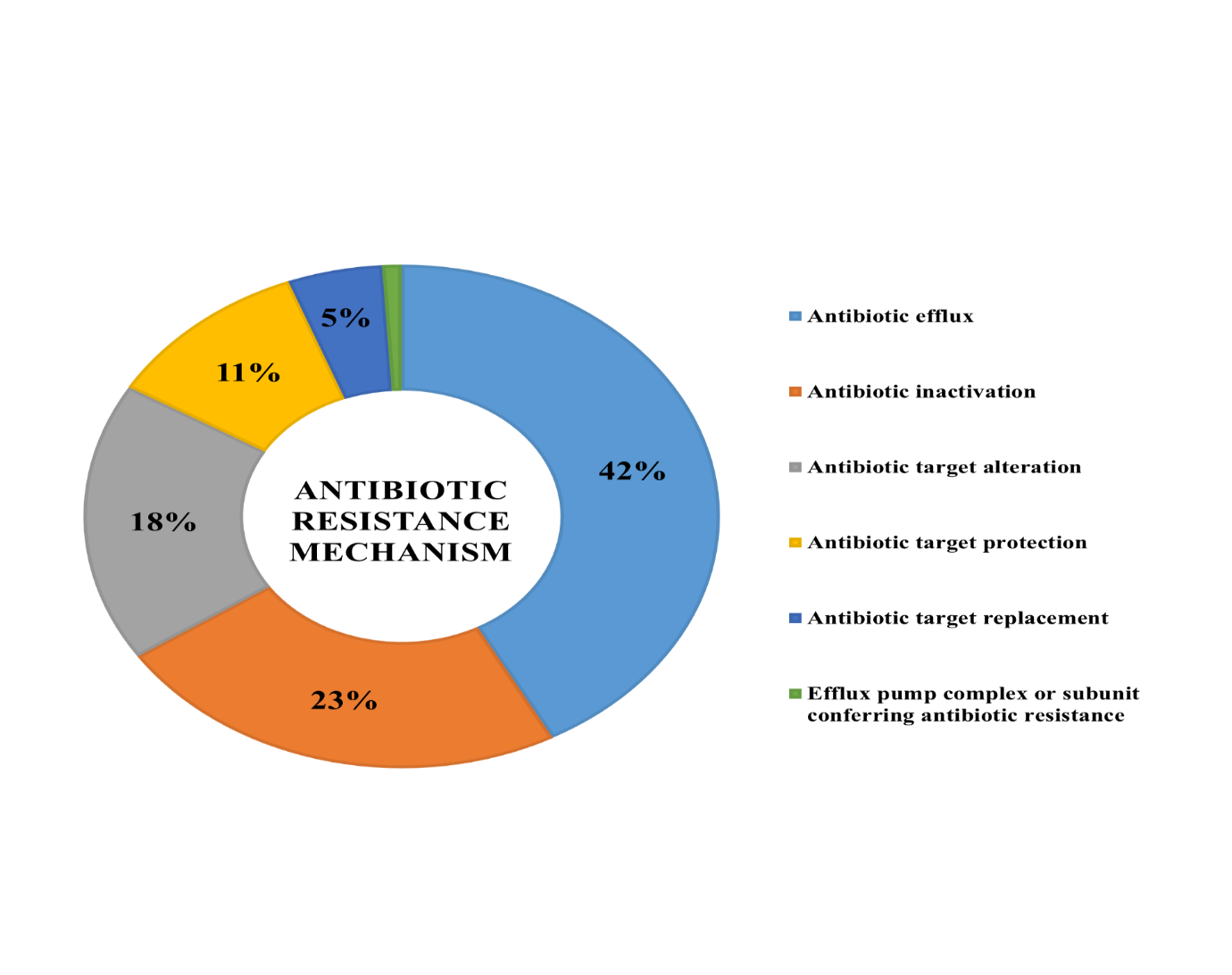


**Supplementary Fig.14**: The pie chart illustrating the percentage of AMR mechanisms exhibited by the identified ARGs in the polluted soil samples.


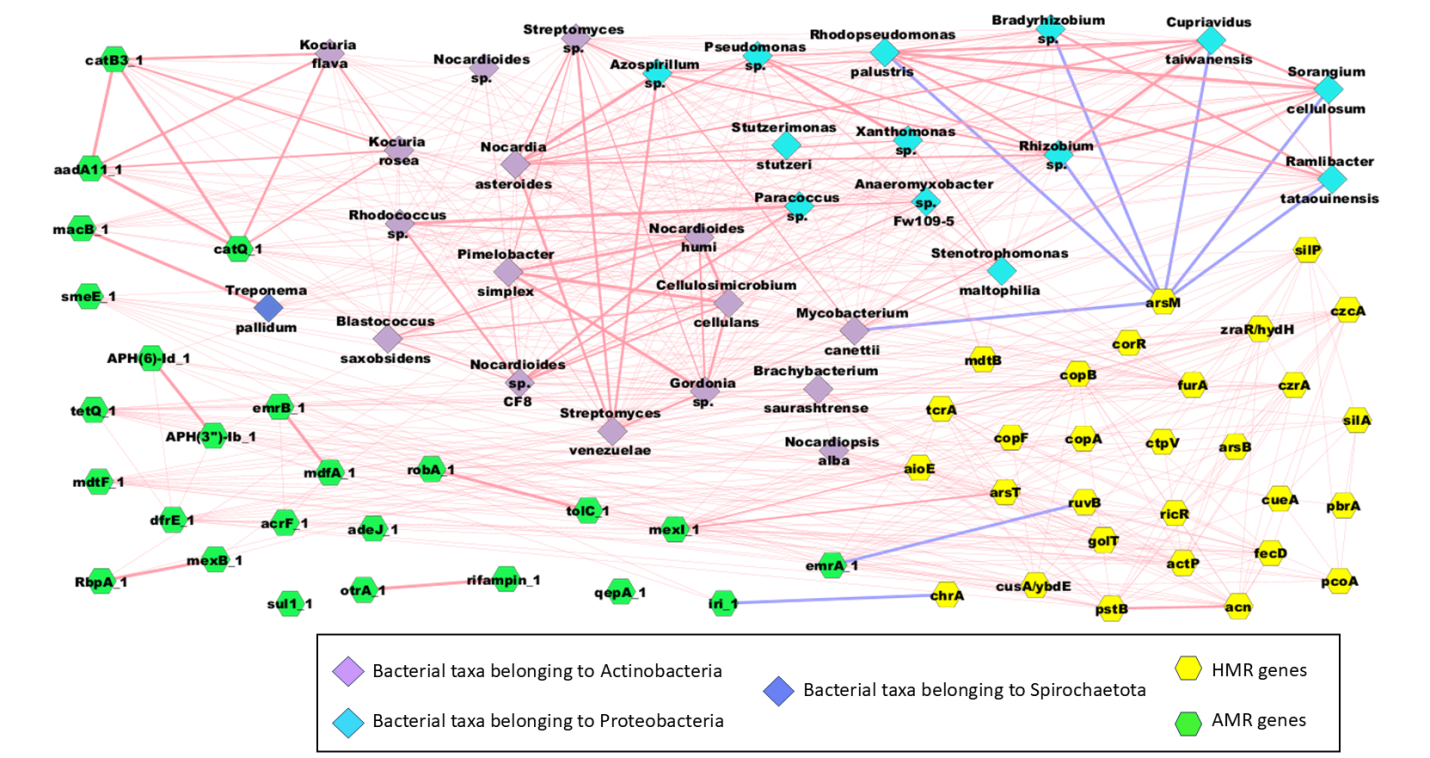


**Supplementary Fig.15**: Network analysis depicting the Pearson correlations between the bacterial taxa, AMR- and HMR- genes.  All the blue edges represent negative correlations, and pink lines represent positive correlations (Pearson correlation coefficient values higher than 0.75 and lower than -0.75 were selected to generate the correlation network in Cytoscape v3.10.2 with the Metscape plugin). The correlation network depicts the negative and positive correlations existing between the bacterial taxa, AMR and HMR genes.


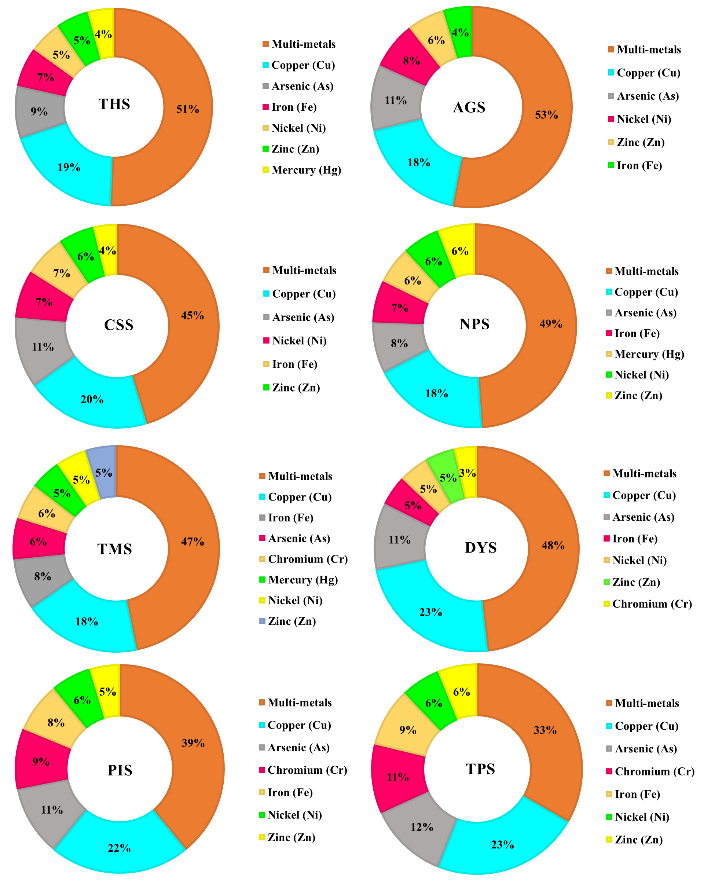


**Supplementary Fig.16**: The abundances and the counts of HRGs (Heavy metal resistance genes) across the soil samples. All HRGs were classified based on the Heavy metal for which they showed resistance.


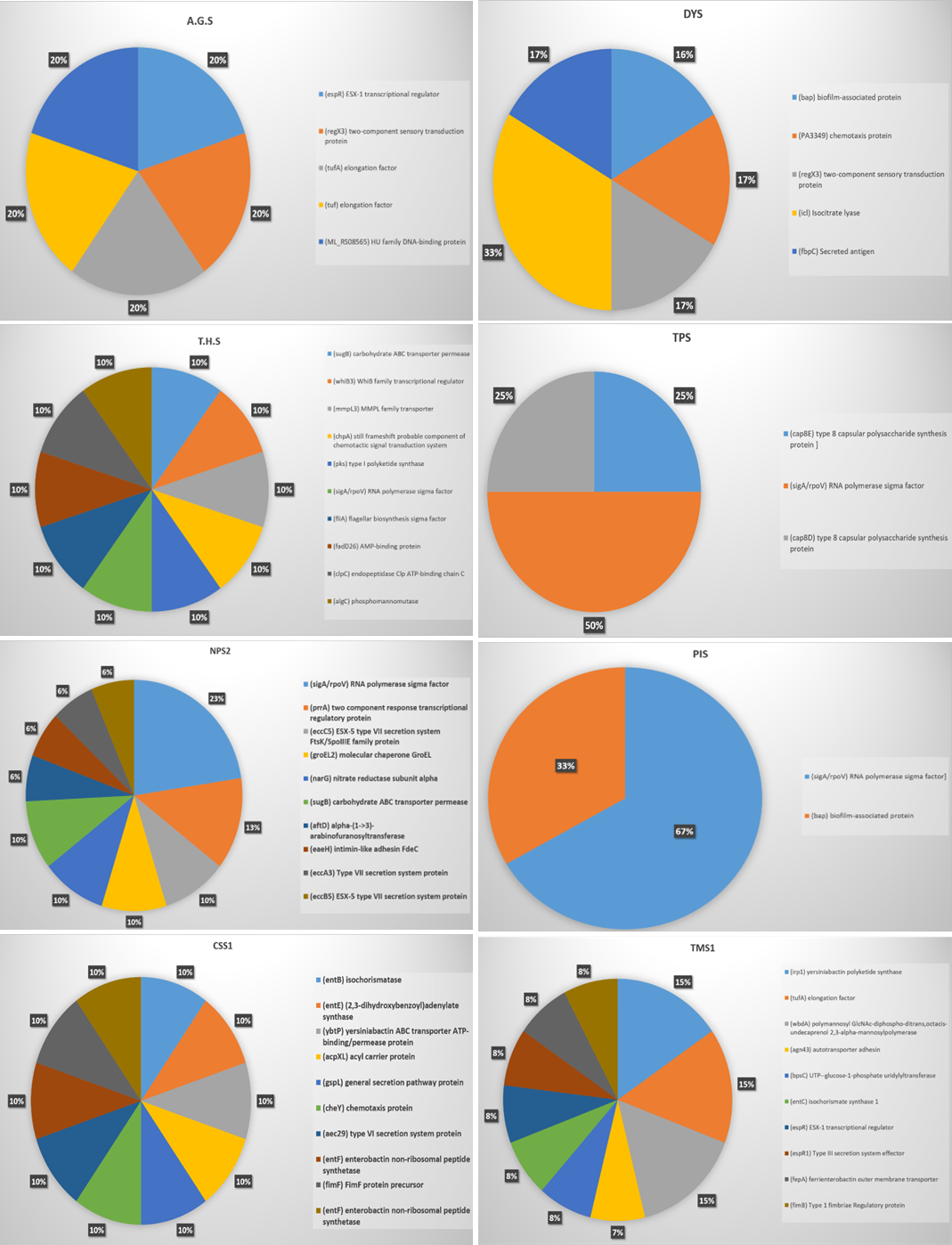


**Fig.17:** Pie chart represents common virulence genes detected across the samples


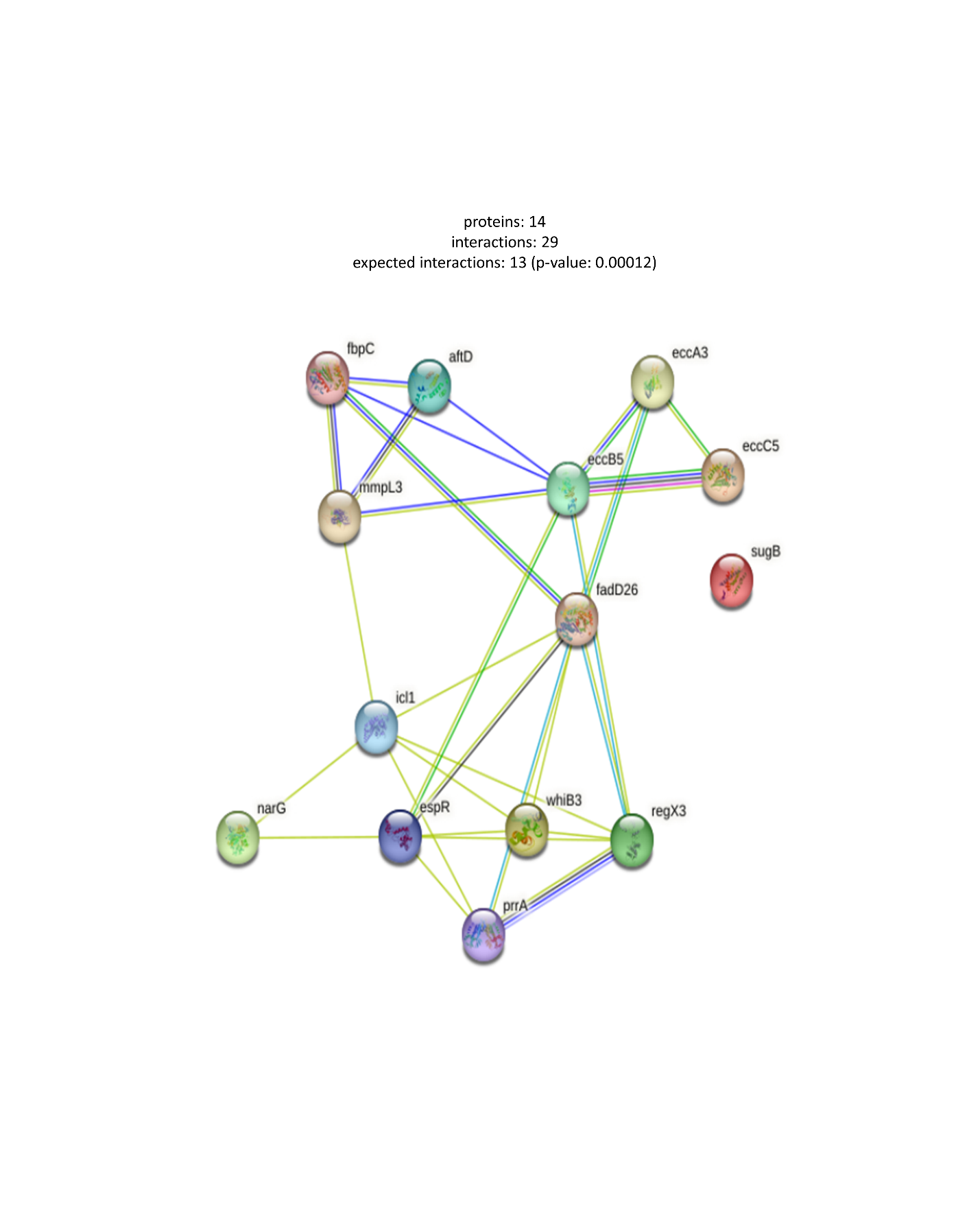


**Supplementary Fig.18**: Shiny GO 8.0 on the basis STRING database was utilized to identify a network of proteins (PPI-Protein Protein Interactions) interacting based on genes whose expression levels significantly change across the sample.
